# Autophagic digestion *of Leishmania major* by host macrophages is associated with differential expression of BNIP3, CTSE, and the miRNAs miR-101c, miR-129, and miR-210

**DOI:** 10.1186/s13071-015-0974-3

**Published:** 2015-07-31

**Authors:** Benjamin Frank, Ana Marcu, Antonio Luis de Oliveira Almeida Petersen, Heike Weber, Christian Stigloher, Jeremy C. Mottram, Claus Juergen Scholz, Uta Schurigt

**Affiliations:** Institute for Molecular Infection Biology, University of Wuerzburg, Josef-Schneider-Str. 2/D15, 97080 Wuerzburg, Germany; Wellcome Trust Centre for Molecular Parasitology, Institute of Infection, Immunity and Inflammation, University of Glasgow, Glasgow, G12 8TA UK; Laboratório de Patologia e Biointervenção, Fundação Oswaldo Cruz-BA, Salvador, Bahia Brazil; Interdisciplinary Center for Clinical Research (IZKF), University of Wuerzburg, Wuerzburg, Germany; Division of Electron Microscopy, Biocenter of the University of Wuerzburg, Wuerzburg, Germany

**Keywords:** Autophagy, BNIP3, CTSE, Electron tomography, *Leishmania major*, Macrophages, miRNAs, MTOR, siRNAs, Transmission electron microscopy

## Abstract

**Background:**

Autophagy participates in innate immunity by eliminating intracellular pathogens. Consequently, numerous microorganisms have developed strategies to impair the autophagic machinery in phagocytes. In the current study, interactions between *Leishmania major* (*L. m.*) and the autophagic machinery of bone marrow-derived macrophages (BMDM) were analyzed.

**Methods:**

BMDM were generated from BALB/c mice, and the cells were infected with *L. m.* promastigotes. Transmission electron microscopy (TEM) and electron tomography were used to investigate the ultrastructure of BMDM and the intracellular parasites. Affymetrix® chip analyses were conducted to identify autophagy-related messenger RNAs (mRNAs) and microRNAs (miRNAs). The protein expression levels of autophagy related 5 (ATG5), BCL2/adenovirus E1B 19 kDa protein-interacting protein 3 (BNIP3), cathepsin E (CTSE), mechanistic target of rapamycin (MTOR), microtubule-associated proteins 1A/1B light chain 3B (LC3B), and ubiquitin (UB) were investigated through western blot analyses. BMDM were transfected with specific small interfering RNAs (siRNAs) against autophagy-related genes and with mimics or inhibitors of autophagy-associated miRNAs. The infection rates of BMDM were determined by light microscopy after a parasite-specific staining.

**Results:**

The experiments demonstrated autophagy induction in BMDM after *in vitro* infection with *L. m.*. The results suggested a putative MTOR phosphorylation-dependent counteracting mechanism in the early infection phase and indicated that intracellular amastigotes were cleared by autophagy in BMDM in the late infection phase. Transcriptomic analyses and specific downregulation of protein expression with siRNAs suggested there is an association between the infection-specific over expression of BNIP3, as well as CTSE, and the autophagic activity of BMDM. Transfection with mimics of mmu-miR-101c and mmu-miR-129-5p, as well as with an inhibitor of mmu-miR-210-5p, demonstrated direct effects of the respective miRNAs on parasite clearance in *L. m.*-infected BMDM. Furthermore, Affymetrix® chip analyses revealed a complex autophagy-related RNA network consisting of differentially expressed mRNAs and miRNAs in BMDM, which indicates high glycolytic and inflammatory activity in the host macrophages.

**Conclusions:**

Autophagy in *L. m.*-infected host macrophages is a highly regulated cellular process at both the RNA level and the protein level. Autophagy has the potential to clear parasites from the host. The results obtained from experiments with murine host macrophages could be translated in the future to develop innovative and therapeutic antileishmanial strategies for human patients.

**Electronic supplementary material:**

The online version of this article (doi:10.1186/s13071-015-0974-3) contains supplementary material, which is available to authorized users.

## Background

Leishmaniasis is one of the 13 most important tropical diseases according to the World Health Organization (WHO) (http://www.who.int/en/). This disease causes serious public health issues worldwide [1–3]. Leishmaniasis is a protozoan disease caused by eukaryotic pathogens from the genus *Leishmania*. The infecting species and the host immune response determine the severity of leishmaniasis as well as the clinical symptoms, which are classified into the cutaneous form, the mucocutaneous form, and the visceral form (http://www.who.int/en/).

The life cycle of the pathogen comprises two morphological stages: the promastigote stage and the amastigote stage. The lancet-shaped, motile promastigotes with external flagella live and multiply in the midgut of female sand flies. They are transmitted when the sand fly consumes a blood meal from a vertebrate host (e.g., humans), and the promastigotes are ingested by phagocytes. Among phagocytic cells, macrophages and dendritic cells (DCs) are the most important interaction partners of *Leishmania* parasites, and these cells regulate the outcome of the early infection phase [4]. The internalized parasite can be located in the cytoplasm or in the parasitophorous vacuoles in the phagocytes [5]. In macrophages, which are the primary host cells for *Leishmania* replication and survival, promastigotes differentiate into roundish, internally flagellated, immotile amastigotes. Both life stages use multiple strategies to manipulate the microbicidal host cell functions and to escape from the host immune system [6]. Understanding the interactions between the parasites and host cells during uptake, differentiation, intracellular replication, and release might be the key for developing new drugs through target-directed approaches.

Autophagy is a catabolic process characterized by degradation of cellular components through the lysosomal machinery. This mechanism is used by eukaryotic cells to ensure that energy is produced during starvation conditions. Additionally, autophagy in mammalian cells, including macrophages, is frequently involved in the degradation of intracellular bacteria, viruses, and parasites [7]. Pathogens in the host cell cytoplasm of infected cells that escaped phagolysosomal degradation typically lead to the induction of autophagy and are consumed through autophagolysosomal digestion. However, numerous microbes have developed strategies to avoid degradation. Some intracellular microorganisms even take advantage of this cellular process to support the infection [8].

To date, autophagy induction in promastigotes and amastigotes of *Leishmania amazonensis, L. m.*, or *Leishmania mexicana* has been repeatedly observed [9–14], and it has been confirmed that autophagy plays a role in parasite nutrition, differentiation, and virulence during the infection of host cells [9–14]. However, the induction of autophagic vacuoles in host macrophages after parasite infection has been reported only for infections with *Leishmania amazonensis* [15, 16]. Similarly, a clinical study reported induced autophagy in *Leishmania donovani*-infected bone marrow cells, which is a phenotype that ceased after the patient was treated with the anti-leishmanial drug amphotericin B [17].

**Fig. 1:**
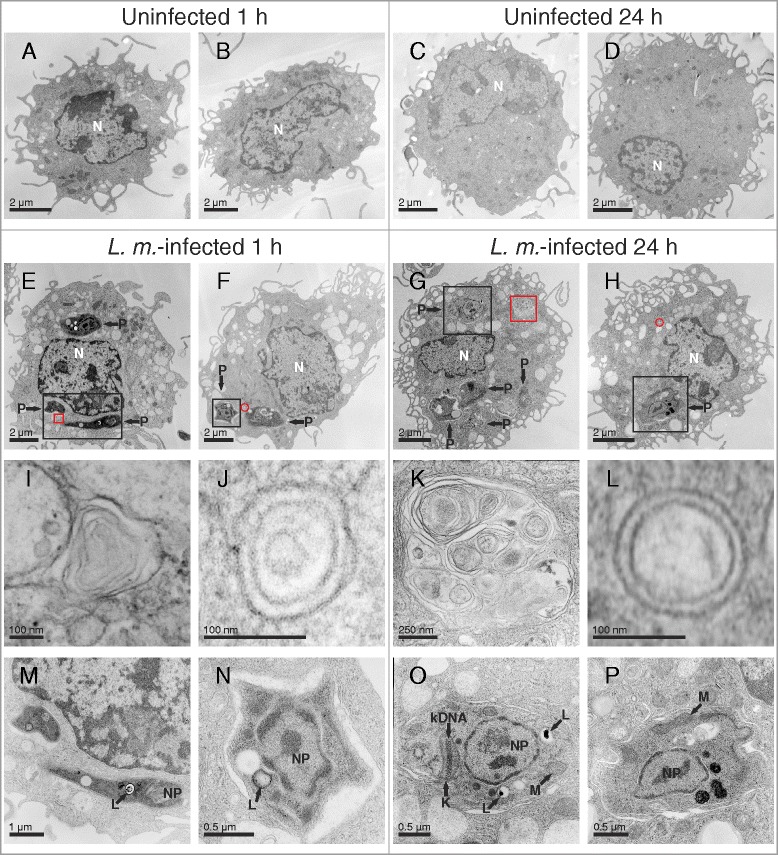
Ultrastructural investigation of autophagy induction in *L. m.*-infected BMDM with TEM. Methods: BMDM from BALB/c mice were infected with *L. m.* promastigotes for (**e**, **f**, **i**, **j**, **m**, **n**) 1 h and (**g**, **h**, **k**, **l**, **o**, **p**) 24 h. **a**–**d** Uninfected BMDM were incubated for the same amount of time in RPMI medium. All BMDM were subjected to TEM analyses. Results: Autophagic phenotypes characterized by (**e**–**h**) a strong vacuolization, (**i**, **k**) presence of MLS and (**j**, **l**) autophagosomes detected in *L. m*.-infected BMDM 1 h p.i. and 24 h p.i. compared to uninfected control BMDM. Details in images (**i**–**p**) were magnified from images (**e**–**h**) from sections of *L. m.*-infected BMDM (red squares = MLS in **i** and **k**, red circles = autophagosomes in **j** and **l**, black squares = intracellular parasites in **m**–**p**). K = kinetoplast, kDNA = kinetoplastid DNA, L = lysosome-like vacuole, M = mitochondrion, N = nucleus of macrophage, NP = nucleus of parasite, P = parasite

In the present study, the observation of an autophagic phenotype of BMDM after infection with *L. m.* promastigotes (Additional file 1: Figure S1) was reported for the first time. This phenotype was characterized by the increased presence of autophagosomes, vacuoles, and myelin-like structures (MLS) [15, 16, 18–22]. These typical morphological features for autophagy were primarily observed in the early (1 h post infection [p.i.]) and the late infection phases (24 h p.i.) in *L. m.*-infected BMDM. The first time point (1 h p.i.) was characterized by an incomplete differentiation of promastigotes to amastigotes (Fig. 1m and n), and this process was completed after 24 h p.i. (Fig. 1o and p).

It has been assumed that amastigotes completely adapt the harsh intracellular conditions in their host macrophages. Therefore, high resistance against intracellular digestion would be expected for amastigotes in BMDM [6, 23]. However, the present study contradicts this assumption and definitively demonstrates that the autophagic machinery can clear an *L. m.* infection from BMDM *in vitro*. Additionally, there is evidence that a complex autophagy-related RNA network and differentially expressed proteins participate in this degradation process.

## Methods

### Nomenclature of genes and proteins

The murine genes and proteins were named using the “Guidelines for Nomenclature of Genes, Genetic Markers, Alleles, and Mutations in Mouse and Rat” provided by the Mouse Genome Informatics (MGI) (http://www.informatics.jax.org/mgihome/nomen/index.shtml). According to these guidelines, murine gene symbols are written in italics, beginning with an uppercase letter, followed by all lowercase letters. In contrast, murine protein symbols are not italicized and use all uppercase letters.

For human genes and proteins, the “Guidelines for Human Gene Nomenclature” of the Human Genome Organization (HUGO) Genome Nomenclature Committee (HGNC) were used (http://www.genenames.org/about/guidelines). As reported by these guidelines, human gene symbols are written in italics and use all uppercase letters, though, human protein symbols are not italicized and use all uppercase letters.

### Strains and maintenance of Wild-Type (WT) parasites

The cloned virulent *L. m.* isolate (strain: MHOM/IL/81/FE/BNI), which was used for infecting BMDM, was maintained by passages in female BALB/c mice. The promastigotes were grown *in vitro* in blood agar cultures at 27 °C and 5 % CO_2_.

The *L. m.* isolate (strain: MHOM/JL/80/Friedlin), which was used for infection of the RAW 264.7 macrophages, was cultivated in modified minimal Eagle’s medium (designated HOMEM, Life Technologies, 11095–080) supplemented with 10 % heat-inactivated fetal calf serum (FCS, Life Technologies, 10108–157) and 1 % penicillin streptomycin solution (Sigma-Aldrich, P4333) at 25 °C and 5 % CO_2_.

### Ethical approval

The *in vivo* passages of *L. m.* parasites (strain: MHOM/IL/81/FE/BNI) in BALB/c mice were approved by the local government commission for animal protection (responsible authority: “Regierung von Unterfranken”; reference number: 55.2-2531.01-26/12).

### Infection of macrophages with *L. m.* promastigotes

BMDM from female BALB/c mice (aged 7–10 weeks) were generated as previously described [24]. After the cells were cultured, BMDM were harvested and seeded in suspension culture plates with a cell concentration of 2 × 10^5^ × ml^−1^ in Roswell Park Memorial Institute medium 1640 (RPMI, Life Technologies, 31870–025) with 10 % FCS (PAA Laboratories, A15-102), 2 mM L-glutamine (Biochrom, K0282), 10 mM 4-(2-hydroxyethyl)-1-piperazineethanesulfonic acid (Hepes, Life Technologies, 15630–056), 0.05 mM 2-mercaptoethanol (Sigma-Aldrich, M7154), 100 U × ml^−1^ penicillin (Sigma-Aldrich, P3032), and 50 μg × ml^−1^ gentamycin (Sigma-Aldrich, G1272). The cells were incubated for 4 h at 37 °C. During this time, the macrophages attached to the plastic surface of the culture dishes. Stationary-phase *L. m.* promastigotes (strain: MHOM/IL/81/FE/BNI) were directly harvested from the blood agar plates, washed twice with phosphate-buffered saline (PBS, Life Technologies, 14190–094) and resuspended in RPMI medium. Finally, the BMDM were infected at a ratio of 1:15 by exchanging the old culture medium with the *L. m.* promastigote cell suspension (3 × 10^6^ × ml^−1^). Cocultures of BMDM with parasites were incubated for 1 and 24 h at 37 °C and 5 % CO_2_. For the time course analyses, BMDM were infected with *L. m.* promastigotes and incubated for 0.5, 1, 2, 4, 10, 24, 27, 30, and 48 h. To isolate proteins for the LC3B western blots, control and *L. m.*-infected macrophages were treated for 1 h with 100 nM bafilomycin A1 (Baf A1, Sigma-Aldrich, B1793) dissolved in dimethyl sulfoxide (DMSO, AppliChem, A3006) before lysis. Control cocultures for the Baf A1 experiments contained 0.5 % DMSO.

RAW 264.7 macrophages were infected with *L. m.* promastigotes (strain: MHOM/JL/80/Friedlin) at a ratio of 1:15. Cocultures of RAW 264.7 macrophages with *L. m.* were incubated in RPMI medium supplemented with 10 % FCS, 2 mM L-glutamine and 50 μg × ml^−1^ gentamycin. The cocultures were incubated for 0.5 h or 24 h at 37 °C and 5 % CO_2_.

### Induction of autophagy in BMDM with Hank’s Balanced Salt Solution (HBSS) or rapamycin treatment

BMDM were harvested and seeded in culture dishes followed by a 4 h incubation to facilitate attachment of the macrophages to the plastic surface. Finally, the medium was replaced by fresh RPMI medium containing 500 nM rapamycin (Calbiochem, 553210), or by HBSS (Life Technologies, 14175–046), to induce autophagy in BMDM. Under these conditions, BMDM were incubated for 1 h.

### TEM

BMDM and RAW 264.7 macrophages were harvested from Petri dishes with a cell scraper and centrifuged to form a pellet (4 °C, 300 × g, 10 min). No washing steps with PBS were performed to avoid inducing autophagy in the macrophages through cell starvation. The macrophages were immediately fixed with 2.5 % glutaraldehyde solution (Sigma-Aldrich, G4004) after they were harvested. The embedding and cutting for TEM analyses was performed as recently described [11]. The contrast agents osmium tetroxide and uranyl acetate were used for TEM. The samples were imaged with an EM900 transmission electron microscope (Zeiss).

### Electron tomography

Samples of *L. m.*-infected BMDM 24 h p.i. were processed as described in the TEM passage with the following modifications. Embedded samples were cut into approximately 250 nm thick slices. Afterwards, the sections were treated with 2.5 % uranyl acetate in ethanol for 15 min and lead citrate for 10 min before the sections were coated with carbon. Then, the sections were treated with 12 nm ProtA-Au-beads to provide fiducials for automated image alignment. A tilt image series was conducted from +70° to −70° with 1° increments at 200 kV with a JEM-2100 TEM (JEOL) and a TemCam-F416 camera (TVIPS) using the SerialEM software (Boulder Laboratory; (http://bio3d.colorado.edu/SerialEM/)) for automation [25]. The ETomo/IMOD software package (Boulder Laboratory; (http://bio3d.colorado.edu/imod/)) was used for the tilt image series alignment and tomographic reconstruction [26]. The reconstructed tomograms were exported as mp4-files using ImageJ version 1.49 g (National Institutes of Health [NIH]).

### Assessment of autophagy in BMDM by TEM

The formation of vacuoles and the development of MLS are hallmarks of autophagy [15, 16, 18–22]. A semiquantitative score was applied to assess the autophagic phenotypes in *L. m.*-infected BMDM and control macrophages. First, the macrophages were imaged with 1600 × magnification by TEM. The area occupied by vacuoles and the presence of MLS were analyzed in sections from 50 individual macrophages. Scores between 0 and 3 were used to distinguish between different rates of vacuolization: 0 (light vacuolization) = 0–25 % of the cytoplasm area contained vacuoles, 1 (medium vacuolization) = 26–50 % of the cytoplasm area contained vacuoles, 2 (strong vacuolization) = 51–75 % of the cytoplasm area contained vacuoles, and 3 (heavy vacuolization) = more than 75 % of the cytoplasm area contained vacuoles. The presence of MLS was also assessed with a scoring system: 0 = no MLS detected in the investigated cell section, and 1 = detection of MLS in the cell section. The total autophagy score was calculated as the sum of vacuolization and presence of MLS scores. Therefore, the highest possible total autophagy score was 4. For each sample, the average of the total autophagy score for 50 analyzed BMDM samples was calculated. Additionally, the frequency of MLS in *L. m.*-infected and uninfected control BMDM was calculated by dividing the numbers of BMDM samples with MLS by the total number of MLS observed.

Statistical analyses to compare total autophagy scores, or the frequency of MLS of analyzed samples, were performed by using the Mann–Whitney *U* test in SPSS software version 20.0.0 (IBM).

### Determining infection rates and nucleus-kinetoplast distances

BMDM were infected as described above. The *L. m.*-infected BMDM and control BMDM were incubated for time points ranging from 0.5 to 48 h. After incubation, 2 × 10^5^ BMDM were transferred to Cytospin tubes (Thermo Scientific). BMDM were attached to object slides by centrifugation at 1500 rpm for 5 min with a Shandon Cytospin3 (Thermo Scientific). Afterwards, the slides were fixed and stained with a Diff-Quik kit (Medion Diagnostics, 130832) according to the manufacturer’s protocol. The slides were analyzed with an Eclipse 50i light microscope (Nikon) using NIS Elements software version 3.22.11 (Nikon). To calculate the average infection rates, the number of intracellular parasites per individual macrophage for each analyzed sample was determined. For each sample, 50 individual macrophages were analyzed.

During differentiation of *L. m.* promastigotes (0 h p.i.) to amastigotes (24 h p.i.), the nucleus-kinetoplast distance shortened significantly from approximately 4 μm to 1.8 μm. The average nucleus-kinetoplast distances were determined by measuring the distances of 50 individual intracellular parasites with an Eclipse 50i light microscope (Nikon) and NIS Elements software version 3.22.11 (Nikon). Statistical significance for the average infection rates, or the average nucleus-kinetoplast distances, was tested with a *t*-test in SPSS software version 20.0.0 (IBM).

### RNA isolation, Affymetrix® chip hybridization, and analyses of expression data

Total RNA from *L. m.*-infected BMDM and uninfected control BMDM was isolated with the RNeasy Mini kit (Qiagen, 74104) and homogenized with QIAshredder (Qiagen, 79656) for the analyses of differential mRNA expression. Isolation of total RNA was performed according to the manufacturer’s protocol. On-column DNase digestion was performed with the RNase-Free DNase Set (Qiagen, 79254). The samples for miRNA transcriptomic analysis were isolated with Trizol (Life Technologies, 15596–026) according to the manufacturer’s instructions. The concentrations of the RNA isolates were measured with NanoDrop spectrophotometry (NanoDrop 1000, Thermo Scientific). Samples were aliquoted and stored at −80 °C until they were used.

RNA degradation was assessed with a Bioanalyzer 2100 (Agilent), and the observed RNA integrity numbers (RINs) ranged between 8.8 and 9.5. 10 is the highest possible RIN. For mRNA analyses, 100 ng total RNA was transcribed *in vitro*, biotin-labeled (IVT-Express kit, Affymetrix, 901229) and hybridized to GeneChip Mouse Genome 430 2.0 microarrays (Affymetrix, 900497). The samples for hybridization with GeneChip miRNA 3.0 (Affymetrix, 902018) were processed with the FlashTag biotin HSR RNA Labeling kit (Affymetrix, 901910) using 1 μg total RNA as starting material. The signals from streptavidin-phycoerythrin stains were detected with a GeneChip Scanner 3000 7G (Affymetrix). The microarray readout from the mRNA probes with a sequence match in the *L. m.* genome were excluded from the analysis to avoid confounding the host expression values due to cross-hybridization with parasite transcripts [27]. The raw microarray signals were normalized with variance stabilization (mRNA) or quantiles normalization (miRNA) and summarized to probe set expression values using the Robust Multi-array Average (RMA) algorithm [28, 29]. Prior to comparing the infected versus control BMDM samples, the probe sets exclusively displaying expression changes between the uninfected control samples taken at 1 and 24 h p.i. were excluded from the analysis to minimize the detection of effects from culture conditions. Statistical significance was tested using the false discovery rate (FDR) according to the Benjamini and Hochberg method. The genes displaying globally significant expression changes (FDR < 0.05) after *L. m.* infection of BMDM were subjected to category enrichment analyses. For these analyses the Kyoto Encyclopedia of Genes and Genomes (KEGG) and the Gene Ontology (GO) databases were used (http://www.genome.jp/kegg/, http://geneontology.org/). The KEGG database provides information about genes and biological pathway maps for transcriptomic analyses. Moreover, the GO database can be used to classify genes into different categories. Therefore, differentially expressed genes of *L. m.*-infected BMDM 24 h p.i. could be compared to KEGG pathway maps to identify significantly regulated pathways (FDR < 0.05). Additionally, differentially expressed genes of *L. m.*-infected BMDM 24 h p.i. were classified into GO categories to identify their roles in biological processes and their molecular function. The data preprocessing, visualization and detection of differentially expressed genes were performed with the Expression Console v1.2.1.20 (Affymetrix), and in the R environment (http://www.r-project.org) using the Bioconductor packages “affy”, “limma”, “made4”, and “vsn” (available at (http://www.bioconductor.org)). The over-representation of gene expression changes in the KEGG pathways and GO categories (“category enrichment analyses”) were detected with the Gene Set Enrichment Analysis (GSEA) [30]. The raw and preprocessed microarray data were deposited in MIAME compliant form at the Gene Expression Omnibus (GEO; (http://www.ncbi.nlm.nih.gov/geo)) in entries GSE52624 (mRNA) and GSE58369 (miRNA). The autophagy-related protein-protein interaction data were recently published by Behrends and colleagues and were retrieved from the original publication as well as the cited database ([31], http://besra.hms.harvard.edu/ipmsmsdbs/comppass.html). The miRNA target interactions were examined with the TargetScan v6.2 database [32]. Cytoscape v3.1.0 was used for network visualization [33].

### Western blot analyses

Proteins from *L. m.*-infected BMDM and control macrophages were isolated with RIPA buffer (Cell Signaling Technology, 9806) for western blot analyses of ATG5, BNIP3, CTSE, β-Actin (ACTB), macrophage migration inhibitory factor (MIF), MTOR, phosphorylated MTOR (p-MTOR), ribosomal protein S6 (RPS6), phosphorylated RPS6 (p-RPS6), and UB. The samples isolated with RIPA buffer were processed according to the manufacturer’s protocol. For LC3B western blot analyses, proteins were isolated with Laemmli buffer according to a protocol developed to investigate LC3B lipidation [34]. Finally, all samples were aliquoted and stored at −20 °C until they were used. The proteins were size-separated by sodium dodecyl sulfate polyacrylamide gel electrophoresis (SDS-PAGE). Western blots were performed according to the datasheets of the individual primary antibodies. Antibodies, including ACTB (#4970), ATG5 (#12994), BNIP3 (#3769), LC3B (#3868), MTOR (#2972), p-MTOR (#2971), p-RPS6 (#2211), RPS6 (#2217), and UB (#3936), were purchased from Cell Signaling Technology. The antibodies against CTSE (sc-30055) and MIF (sc-20121) were purchased from Santa Cruz Biotechnology. The secondary antibodies for all primary antibodies were horse radish peroxidase (HRP)-conjugated (Cell Signaling Technology [#7074] for the primary antibodies from Cell Signaling Technology, and secondary antibodies from Santa Cruz Biotechnology [sc-2030] were used for the primary antibodies from Santa Cruz Biotechnology. Subsequently, the binding of secondary antibodies was detected with HRP luminal substrate (Merck Millipore Corporation, WBKLS0100). The luminescence was monitored with Luminescent Image Analyzer ImageQuant LAS 4000 (GE Healthcare Life Sciences). The signal intensities were analyzed with ImageJ version 1.45 s software (NIH). ACTB was the internal loading control for all western blot experiments. Statistical significance was tested using a one-tailed *t*-test in Excel 2013 software (Microsoft).

### Transfection of *L. m.*-infected BMDM with siRNAs, or miRNA mimics or inhibitors

BMDM were transfected using the Amaxa Mouse Macrophage Nucleofector Transfection kit (Lonza, VPA-1009) and the Nucleofector 2b Device (Lonza) according to the manufacturer’s protocol. To analyze infection rates after specific downregulation of ATG5, MTOR, and UB by RNA interference, BMDM were transfected 4 h prior to infection directly after the cells were harvested from suspension plates. Specific siRNAs were purchased from Santa Cruz Biotechnology (*Atg5* siRNA [sc-41446], *Mtor* siRNA [sc-35410], *Ub* siRNA [sc-36770] as well as a negative control siRNA [sc-37007]). To confirm downregulation of corresponding proteins, duplicate samples were isolated with RIPA buffer (Cell Signaling) 2, 8, and 20 h p.i. (6, 12, and 24 h after transfection). Western blots of the respective proteins were performed as described above. Transfections of *L. m.*-infected BMDM with specific siRNAs to downregulate *Bnip3* (sc-37452, Santa Cruz) or *Ctse* (sc-41474, Santa Cruz) were performed 20 h p.i.. Downregulation of BNIP3 and CTSE was confirmed with western blot analyses (see above) at 26, 32, and 44 h p.i. (6, 12, and 24 h after transfection).

To investigate the role of differentially expressed miRNAs identified by Affymetrix**®** chip analyses, *L. m.*-infected BMDM were transfected 20 h p.i.. For upregulated miRNAs, *L. m.*-infected BMDM were transfected with miRNA inhibitors (mmu-miR-155-5p: MIN0000165, Qiagen; mmu-miR-210-5p: MIN0017052, Qiagen). For downregulated miRNAs, *L. m.*-infected BMDM were transfected with miRNA mimics (mmu-miR-101c: MSY0019349, Qiagen; mmu-miR-129-5p: MSY0000209, Qiagen). The negative control for the miRNA mimics or inhibitors was purchased from Qiagen (1027271).

The infection rates of all transfected *L. m.*-infected BMDM were determined 48 h p.i. as described above.

The cytotoxicity of siRNAs and miRNAs against BMDM were tested by alamarBlue® cytotoxicity assay as described previously [24]. Transfection of BMDM with the siRNAs and miRNA mimics or inhibitors had no cytotoxic effects on BMDM (Additional file 2: Figure S2).

### Determination of half maximal Inhibitory Concentration (IC_50_) values

IC_50_s for Baf A1 and rapamycin against *L. m.* amastigotes and BMDM were determined using the amastigote drug screening assay as previously described [24]. Statistical significance was tested using a one-tailed *t*-test in Excel 2013 software (Microsoft).

## Results

### *L. m.* infection induced autophagy in macrophages

BMDM were infected with *L. m.* promastigotes, which were harvested at an early (1 h p.i.) and at a late (24 h p.i.) time point in the infection course and subjected to TEM analyses. Within 24 h, *L. m.* completed the differentiation from the promastigote (Additional file 1: Figure S1) to the amastigote stage inside the macrophages (Fig. 1o and p).

Noteworthy hallmarks of autophagy [15, 16, 18–22], vacuolization of cells (Fig. 1e–h), formation of MLS (Fig. 1i and k), and the presence of double-membraned autophagosomes (Fig. 1j and l) were observed in *L. m.*-infected BMDM 1 h p.i. (Fig. 1e and f) and 24 h p.i. (Fig. 1g and h). Therefore, *L. m.*-infected BMDM phenotypically resembled BMDM treated with rapamycin (Additional file 3: Figure S3A–D), or BMDM starved in HBSS (Additional file 3: Figure S3E–H), which are both well-known autophagy-inducing conditions.

**Fig. 2:**
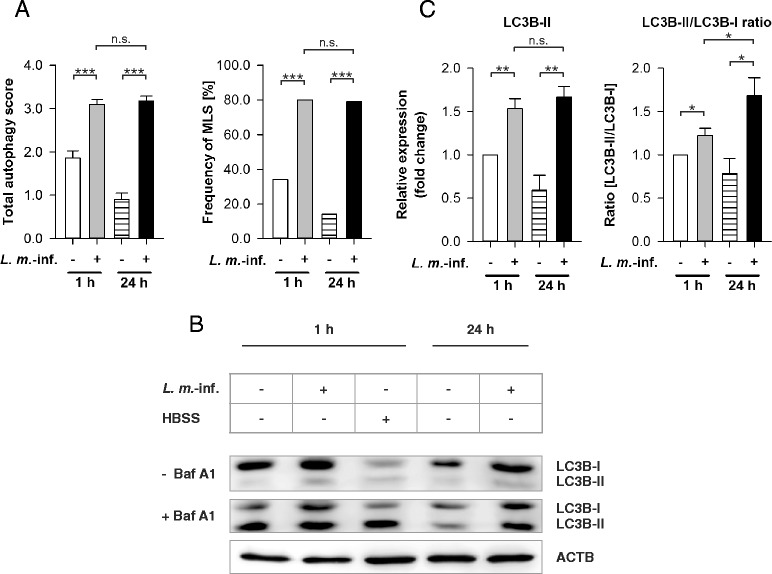
Autophagy assessment in *L. m.*-infected BMDM with TEM and LC3B western blot analyses. Methods: (**a**–**c**) BMDM from BALB/c mice were infected with *L. m.* promastigotes for 1 h or 24 h. Uninfected control BMDM were incubated for the same amount of time in RPMI medium. **a** All BMDM were subjected to TEM analyses. 50 BMDM from each sample were semiquantitatively analyzed for the grade of vacuolization (0 – 3) and the presence of MLS (+1), which resulted in a total autophagy score (maximum = 4). The total autophagy score and frequency of MLS were calculated. **b**, **c** Additionally, proteins were isolated from *L. m.*-infected and uninfected BMDM as well as from HBSS-starved BMDM to monitor autophagy with LC3B western blotting. Cell cultures were partially treated with Baf A1 to monitor autophagic flux. Western blots with proteins from 3 independent experiments were analyzed densitometrically. ACTB served as the internal loading control. Results: (**a**) The total autophagy score and the frequency of MLS were significantly increased in *L. m.*-infected BMDM 1 and 24 h p.i. compared to uninfected controls. There were no significant differences between *L. m.*-infected BMDM 1 and 24 h p.i.. **b** LC3B-II levels in samples from *L. m.*-infected BMDM 1 and 24 h p.i. were increased compared to uninfected controls. An accumulation of LC3B-II was visible in all Baf A1-treated samples (+ Baf A1) compared to controls (− Baf A1), which indicates an autophagic flux. **c** Results of densitometric analyses showed that LC3B-II levels and the LC3B-II/LC3B-I ratios were significantly increased in *L. m.*-infected BMDM 1 and 24 h p.i. compared to uninfected controls. Furthermore, the LC3B-II/LC3B-I ratio of *L. m.*-infected BMDM 24 h p.i. compared to 1 h p.i. was significantly increased. Baf A1 = Bafilomycin A1, *L. m.*-inf. = *L. m.*-infected, n.s. = not significant, * *p* ≤ 0.05, ** *p* ≤ 0.01, *** *p* ≤ 0.001

The semiquantitative analyses of the autophagic phenotype in *L. m.*-infected BMDM 1 and 24 h p.i. clearly demonstrated a significantly higher total autophagy score (Fig. 2a) compared to the uninfected control BMDM at the same time points. There was no significant difference in the scores of *L. m.*-infected BMDM at these time points (Fig. 2a). In addition to calculating the total autophagy score, a strong autophagy induction was also detected by determining of the frequency of MLS in *L. m.*-infected BMDM compared to the uninfected control BMDM (Fig. 2a). Additionally, no significant differences were detected in the total autophagy scores and the frequencies of MLS of *L. m.*-infected BMDM 1 and 24 h p.i. as well as BMDM treated with 500 nM rapamycin for 1 h or starved in HBSS for 1 h (Additional file 3: Figure S3I and S3J). Therefore, the data suggested that the highest rate of vacuolization and MLS formation was already reached in the very early infection phase (<1 h p.i.) of *L. m.*-infected BMDM. Overall, these findings supported the hypothesis of maximum autophagy induction in *L. m.*-infected BMDM at 1 and 24 h p.i..

A comparison of uninfected BMDM revealed a high total autophagy score and an increased frequency of MLS in BMDM 1 h p.i., which dropped significantly until 24 h p.i. (Figs. 2a and 9c). Jaquel and colleagues described this type of infection-independent autophagy as essential during macrophage differentiation induced by macrophage colony-stimulating factor (M-CSF) [35, 36].

**Fig. 3:**
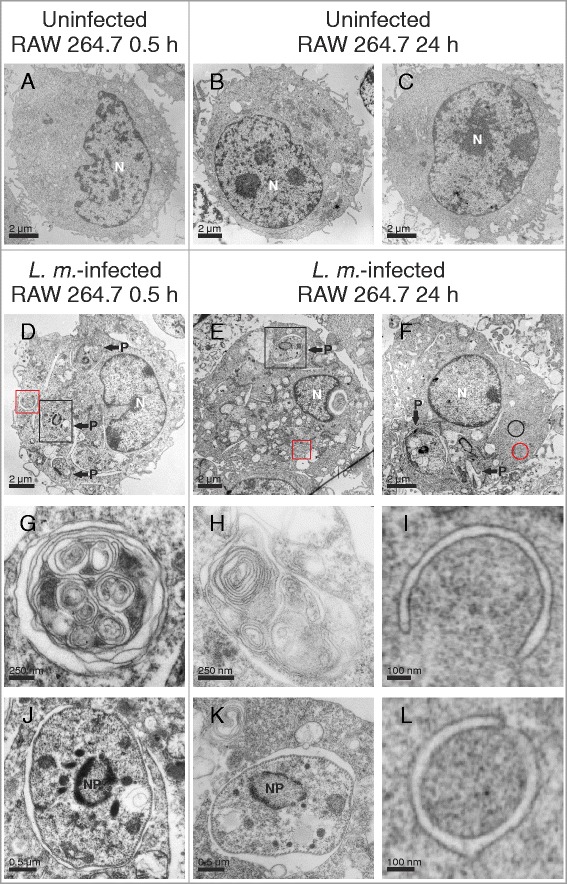
Ultrastructural investigation of autophagy induction in *L. m.*-infected RAW 264.7 macrophages with TEM. Methods: RAW 264.7 macrophages were infected with *L. m.* promastigotes for (**d**, **g**, **j**) 0.5 h and (**e**, **f**, **h**, **i**, **k**, **l**) 24 h. **a**–**c** Uninfected BMDM were incubated for the same amount of time in RPMI medium. All macrophages were subjected to TEM analysis. Results: Autophagic phenotypes characterized by (**d**–**f**) a strong vacuolization, (**g**, **h**) the presence of MLS, (**l**) autophagosomes, and (**i**) phagophores were observed in *L. m.*-infected RAW 264.7 macrophages 0.5 h p.i. and 24 h p.i. compared to uninfected controls. Details in images (**g**–**l**) were magnified from images (**d**–**f**) from a total section of *L. m.*-infected BMDM (red squares = MLS in **g** and **h**, red circle = phagophore in **i**, black circle = autophagosome in **l**, black squares = intracellular parasites in **j** and **k**). N = nucleus of macrophage, NP = nucleus of parasite, P = parasite

Coculture experiments in BMDM followed by TEM were replicated in RAW 264.7 macrophages infected with *L. m.* for 0.5 and 24 h. This immortal cell line displayed the same characteristics of autophagy induction as the effects observed in BMDM (Fig. 3a–l).

In summary, these data revealed that infection with *L. m.* promastigotes induced autophagy in macrophages, which was maintained throughout the differentiation process from promastigotes to amastigotes.

### *L. m.* infection increased LC3B lipidation in BMDM in the early and the late infection phase, and increased LC3B-II/LC3B-I ratio in BMDM in the late infection phase

Apart from observing morphological changes, western blot analyses with LC3B-specific antibodies were used for monitoring autophagy [18]. LC3B appears in two forms: cytosolic LC3B-I and autophagosomal membrane-associated LC3B-II. During autophagy induction, LC3B-I is converted to LC3B-II by conjugation with phosphatidylethanolamine (PE) (= lipidation) [37], which quantitatively correlates with the increased number of autophagosomes [37]. The more LC3B-II is detected, the more autophagosomes are present in the investigated cells. Therefore, this value characterizes the current number of autophagosomes in these cells, but does not indicate changes in the autophagic flux. However, cellular alterations in autophagic activity are best characterized by calculating the LC3B-II/LC3B-I ratio [18, 37, 38]. An increase in the LC3B-II/LC3B-I ratio in the investigated cells compared to the respective control cells indicates increased autophagic activity [18, 37, 38].

Furthermore, treating cells with the ATPase inhibitor Baf A1 is used to qualitatively detect the presence of autophagic flux with LC3B western blot analyses [18, 37]. Cells with autophagic flux show an accumulation of LC3B-II after deacidification of the lysosomal/autophagosomal compartment due to Baf A1. No accumulation of LC3B-II can be observed in cells with complete autophagy inhibition.

In the current experiments, significantly higher amounts of lipidated LC3B-II were detected in samples from *L. m.*-infected BMDM 1 and 24 h p.i. compared to the uninfected controls (Fig. 2b and c). There were no significant differences in LC3B-II protein levels in *L. m.*-infected BMDM at both investigated time points. Therefore, the expression pattern of LC3B-II alone (Fig. 2b and c) correlated with the total autophagy score and the frequency of MLS (Fig. 2a).

Additionally, the ratio of LC3B-II/LC3B-I was significantly increased in *L. m.*-infected BMDM 1 and 24 h p.i. compared to the uninfected control cells at these time points (Fig. 2b and c). However, a significantly lower ratio was detected in *L. m.*-infected BMDM 1 h p.i. compared to infected cells 24 h p.i., which indicates an inhibitory counteracting mechanism in the early infection phase after strong autophagy induction (Fig. 2b and c).

Baf A1 treatment led to a high accumulation of LC3B-II bands compared to the untreated samples and revealed an autophagic flux in *L. m.*-infected BMDM at both investigated time points (Fig. 2b). A significant decrease of LC3B-II accumulation was detected in the uninfected control BMDM 24 h p.i. compared to the uninfected control BMDM 1 h p.i. The cells also showed a reduction in M-CSF-induced autophagy over time.

In conclusion, western blot analyses with LC3B-specific antibodies confirmed the results of the TEM analyses and also produced an inhibitory effect on *L. m.*-induced autophagy in the early infection phase (1 h p.i.).

### Downregulation of ATG5 and UB expression by RNA interference inhibited autophagic digestion of *L. m.* in host macrophages

**Fig. 4:**
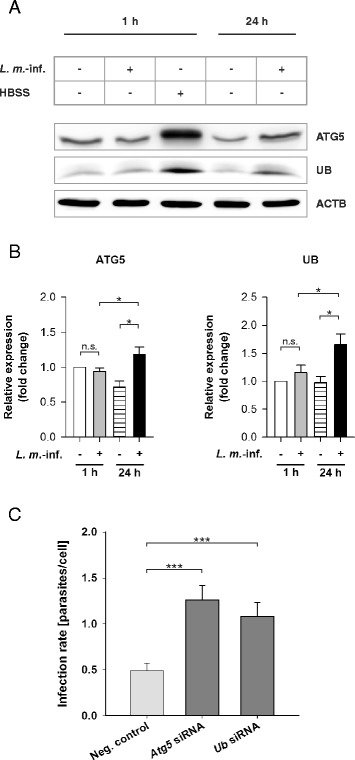
ATG5 and UB western blot analyses with protein extracts from *L. m.*-infected and HBSS-starved BMDM as well as determination of the infection rates of *L. m.*-infected BMDM after ATG5 and UB downregulation by RNA interference. Methods: (**a**, **b**) BMDM from BALB/c mice were infected with *L. m.* promastigotes for 1 and 24 h. Uninfected control BMDM were incubated for the same time in RPMI medium or starved for 1 h in HBSS. The proteins were harvested and subjected to western blot analysis with specific antibodies against ATG5 and UB. Western blots with proteins from 3 independent experiments were analyzed densitometrically. ACTB served as the internal loading control. **c** Additionally, BMDM were transfected with specific siRNAs 4 h prior to infection to downregulate the expression of ATG5 and UB. The cells were finally infected with *L. m.* promastigotes. *L. m.*-infected control BMDM were transfected with negative control siRNA. The infection rates were determined 48 h p.i.. Diagram shows the result of 2 independent experiments. Results: (**a**) ATG5 and UB levels in samples from *L. m.*-infected BMDM 24 h p.i. were increased compared to uninfected control BMDM. **b** Results of the densitometric analyses confirmed that ATG5 and UB were significantly increased in *L. m.*-infected BMDM 24 h p.i. compared to uninfected control BMDM. No upregulation was detected in *L. m.*-infected BMDM 1 h p.i. compared to the respective controls. **c** A significant increase in the infection rate was found in *L. m.*-infected BMDM after downregulation of the protein expression of ATG5 or UB compared to *L. m.*-infected BMDM transfected with negative control siRNA. *L. m.*-inf. = *L. m.*-infected, neg. control = negative control, n.s. = not significant, * *p* ≤ 0.05, *** *p* ≤ 0.001

To confirm autophagy induction in *L. m.*-infected BMDM, the expression of ATG5 and UB, which are two important proteins for autophagic activity, was downregulated by RNA interference. ATG5 is an essential protein for autophagy, especially for autophagosome formation. It is a necessary protein for LC3B-I conjugation to PE to form LC3B-II and for the elongation of autophagic membranes [39]. Western blot analyses demonstrated that ATG5 was overexpressed at the protein level in *L. m.*-infected BMDM 24 h p.i. compared to the respective control cells (Fig. 4a and b). Downregulation of ATG5 expression in BMDM by transfection with specific siRNA prior to infection resulted in significantly decreased ATG5 levels (Additional file 4: Figure S4A) and increased the infection rate significantly at 48 h p.i. (Fig. 4c). This result demonstrated that *L. m.* parasites were cleared from the host macrophages by autophagic digestion.

UB, another autophagy-related protein, was also significantly overexpressed in *L. m.*- infected BMDM 24 h p.i. (Fig. 4a and b). Downregulation of UB expression in BMDM by transfection with specific siRNA prior to infection resulted in significantly decreased UB levels (Additional file 4: Figure S4A) and also led to a significant increase in the infection rate at 48 h p.i. (Fig. 4c). These results indicated that UB might play an important role as an adaptor protein between the parasites and MLS or other autophagic membranes during autophagic clearance of *L. m.* parasites from BMDM, which has been described for many intracellular pathogens that were finally degraded by autophagolysosomes [40, 41].

In summary, these data indicated an ATG5-dependent clearance of *L. m.* parasites in BMDM through autophagic digestion and revealed a putative contribution of UB as an important autophagy-relevant adaptor protein.

### MLS were associated with parasites in the late infection phase

**Fig. 5:**
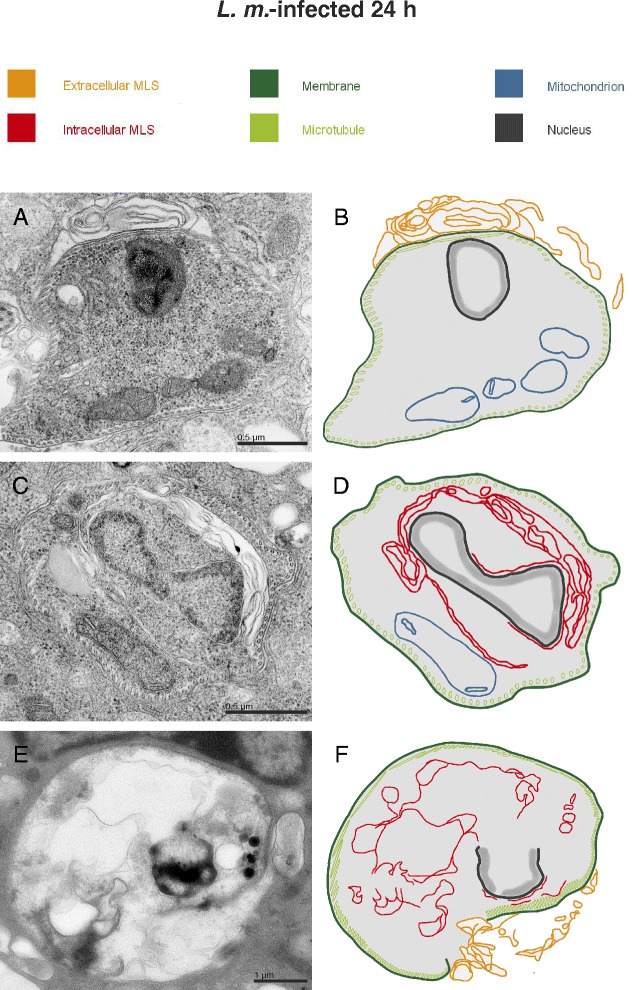
Ultrastructural investigation of parasite-associated localization of MLS with TEM. Methods: BMDM from BALB/c mice were infected with *L. m.* promastigotes for 24 h and subjected to TEM analyses. Results: MLS were frequently observed (**a**) in the cytoplasm of BMDM closely associated with intracellular amastigotes, and (**c**) localized in the cytoplasm of parasites. **e** Additionally, digested amastigotes with a defective cell membrane and remains of intracellular MLS were found in *L. m.*-infected BMDM 24 h p.i.. (**b**, **d**, **f**) Schematic illustrations of images (**a**, **c**, and **e**), respectively

The embedded samples for TEM analyses of *L. m.*-infected BMDM were reanalyzed by TEM and electron tomography to determine if the large parasites, with a length of approximately 2–3 μm, were engulfed by autophagosomes. A strong local association of parasites with extracellular MLS was detected 24 h p.i. (Fig. 5a and b, Additional file 5: Video 1 and Additional file 6: Video 2). However, complete engulfment of *L. m.* parasites in autophagosomes, as it is known for small bacteria, was not visible in the images [20, 42]. Instead, MLS were frequently detected inside the parasites (Fig. 5c and d, Additional file 5: Video 1 and Additional file 7: Video 3). The intracellular appearance of MLS was confirmed by localization of the periplasmic microtubules, which are integrated into the plasma membrane of the parasite. A direct interaction between these parasite tubules and intracellular MLS was frequently observed (Additional file 7: Video 3). Videos also suggested a direct interaction between MLS and the parasite plasma membrane. However, this interaction has to be confirmed in additional and detailed experiments in future. Furthermore, digested amastigotes with a defective cell membrane and the remains of intracellular MLS (Fig. 5e and f) were found.

These results suggested that autophagic digestion of *L. m.* was significantly different compared to the digestion of other intracellular microorganisms and was not associated with complete engulfment of the parasites in autophagosomes. An alternate engulfment mechanism might have been responsible for the autophagic clearance of *L. m.* amastigotes from BMDM.

### Autophagy induction in *L. m.*-infected BMDM was not caused by MTOR cleavage

**Fig. 6:**
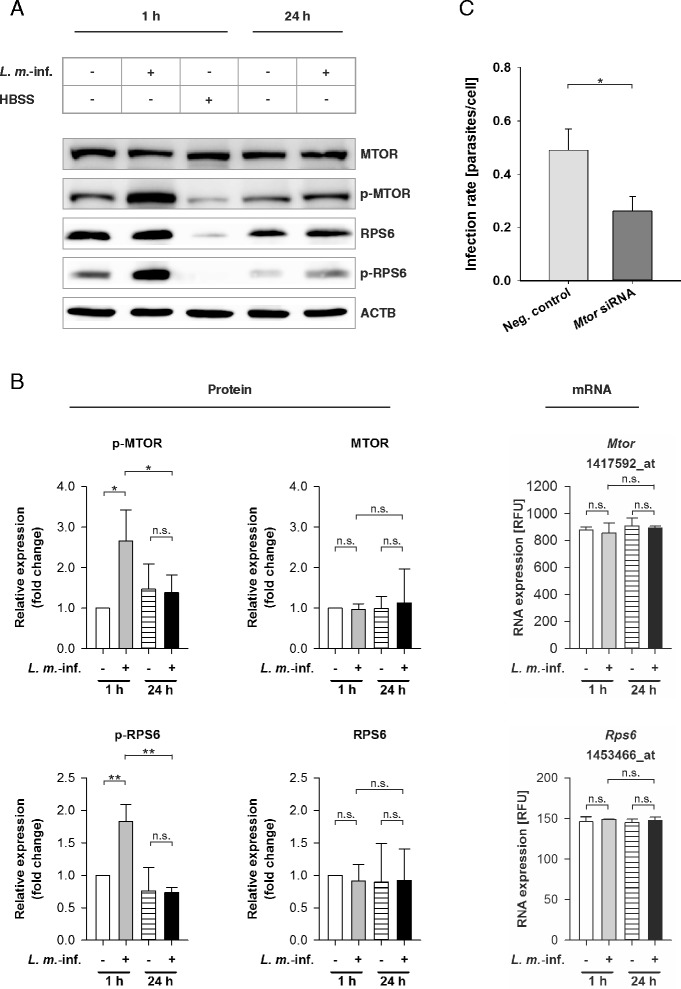
MTOR and RPS6 transcriptomic and western blot analyses with RNAs and protein extracts from *L. m.*-infected and HBSS-starved BMDM, and determination of infection rates of *L. m.*-infected BMDM after MTOR downregulation by RNA interference. Methods: (**a**, **b**) BMDM from BALB/c mice were infected with *L. m.* promastigotes for 1 or 24 h. Uninfected control BMDM were incubated for the same amount of time in RPMI medium or starved for 1 h in HBSS. Proteins were harvested and subjected to western blot analyses with specific antibodies against MTOR, p-MTOR, RPS6, and p-RPS6. Western blots with proteins from 3 independent experiments were analyzed densitometrically. ACTB served as the internal loading control. Total RNA was harvested from *L. m.*-infected BMDM and uninfected control BMDM. The Affymetrix® chips were hybridized with RNA samples from 2 independent experiments were analyzed densitometrically. **c** Additionally, BMDM were transfected with specific siRNA 4 h prior to infection to downregulate the expression of MTOR, and the cells were finally infected with *L. m.* promastigotes. *L. m.*-infected controls were transfected with negative control siRNA. The infection rates were determined 48 h p.i. in 2 independent experiments. Results: (**a**) A significant hyperphosphorylation was observed for MTOR and RPS6 in samples from *L. m.*-infected BMDM 1 h p.i. compared to uninfected control BMDM. **b** Results of densitometric analyses of western blot experiments and Affymetrix® chip analyses showed that MTOR and RPS6 expressions were not regulated at the mRNA or the protein level. However, MTOR and RPS6 were significantly hyperphosphorylated in *L. m.*-infected BMDM 1 h p.i.. **c** A significant decrease in the infection rate was detected in *L. m.*-infected BMDM after downregulation of the protein expression of MTOR compared to *L. m.*-infected BMDM transfected with negative control siRNA. *L. m.*-inf. = *L. m.*-infected, neg. control = negative control, n.s. = not significant, RFU = relative fluorescence units, * *p* ≤ 0.05, ** *p* ≤ 0.01

In the next steps, the MTOR dependency of autophagy induction in BMDM by *L. m.* infection was investigated at the molecular level. Recently, the proteolytical inactivation of MTOR in *L. m.*-infected BMDM by glycoprotein 63 (GP63), a metalloprotease abundantly expressed by *Leishmania* [6, 43], was reported by Jaramillo and colleagues [44]. It is known that inhibition or inactivation of MTOR, a key regulator of autophagy, results in autophagy induction [45]. Therefore, protein lysates from *L. m.*-infected BMDM and the uninfected control BMDM 1 and 24 h p.i. were analyzed with western blot using MTOR-specific antibodies. In contrast to Jaramillo’s study, *L. m.*-infected BMDM showed no evidence of proteolytical inactivation of MTOR by *L. m.* proteases (Fig. 6a and b). Degradation of MTOR was not detected in the early (1 h p.i.) or in the late (24 h p.i.) infection phases. The MTOR-specific signals of *L. m.*-infected macrophages were almost identical in all of the infected samples as well as the uninfected control BMDM (Fig. 6a and b).

Overall, this result indicated that proteolytic degradation of MTOR was not the crucial trigger for autophagy induction in BMDM after infection with *L. m*..

### Transiently increased MTOR phosphorylation in *L. m.*-infected BMDM protected parasites from digestion in the early infection phase

HBSS starvation is a well-characterized autophagy-inducing condition that leads to MTOR hypophosphorylation and decreased kinase activity (Fig. 6a). To investigate if reduced phosphorylation status of MTOR was necessary for autophagy induction during parasite infection, MTOR phosphorylation was monitored in *L. m.*-infected BMDM as well as in the corresponding uninfected controls. However, phosphorylated MTOR was detected in all BMDM. MTOR was even hyperphosphorylated in *L. m.*-infected BMDM 1 h p.i., whereas normal phosphorylation levels were observed 24 h p.i. (Fig. 6a and b). The kinase activity of MTOR was also assessed by monitoring the phosphorylation of the downstream protein RPS6, which reflected the p-MTOR status (Fig. 6a and b). According to the results for p-MTOR, hyperphosphorylation of RPS6 was detected in *L. m.*-infected BMDM 1 h p.i. (Fig. 6a and b). The hyperphosphorylation of MTOR and RPS6 might explain the significantly lower LC3B-II/LC3B-I ratio in *L. m.*-infected BMDM, detected 1 h p.i. compared to 24 h p.i. (Fig. 2b and c). Moreover, downregulation of MTOR expression by transfection with specific siRNA prior to infection resulted in significantly decreased MTOR and p-MTOR levels (Additional file 4: Figure S4A) as well as a decreased infection rate compared to *L. m.*-infected BMDM, which were transfected with negative control siRNA (Fig. 6c). These results indicated that MTOR hyperphosphorylation protected parasites against autophagic digestion in BMDM in the early infection phase.

Remarkably, there was no difference in the phosphorylation status of MTOR and RPS6 in uninfected BMDM 1 and 24 h p.i. (Fig. 6a and b). Therefore, M-CSF-induced autophagy does not appear to be MTOR dependent; although the total autophagy score dropped over time in the uninfected control BMDM (Figs. 2a and 9c).

Taken together, these data suggested that *L. m.*-dependent autophagy was not induced by MTOR hypophosphorylation. Furthermore, MTOR hyperphosphorylation might trigger a counteracting mechanism to prevent autophagic digestion of parasites in the early infection phase.

### Autophagy-, inflammation-, and glycolysis-related genes were differentially expressed in *L. m.*-infected BMDM in the late infection phase

**Fig. 7:**
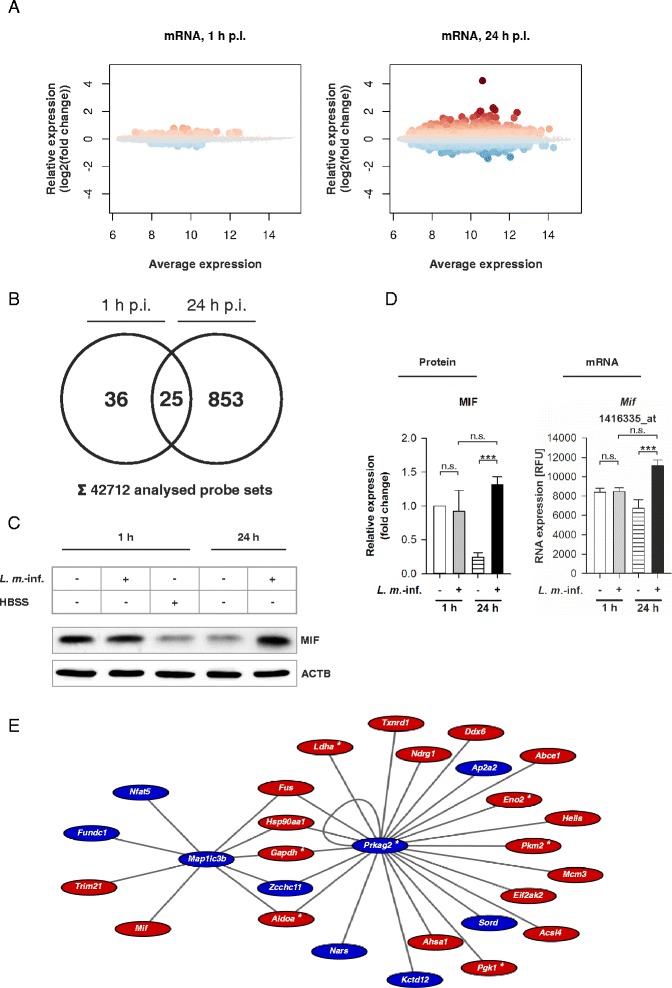
Global analysis of differentially expressed mRNAs in *L. m.*-infected BMDM and MIF western blot analyses with protein extracts from *L. m.*-infected and HBSS-starved BMDM. Methods: (**a**–**e**) BMDM from BALB/c mice were infected with *L. m.* promastigotes for 1 or 24 h. Uninfected control BMDM were incubated for the same amount of time in RPMI medium. Total RNA from 2 independent experiments was harvested from *L. m.*-infected BMDM or uninfected controls and hybridized with Affymetrix® chips. The BMDM were additionally incubated for 1 h in HBSS. Proteins were harvested and subjected to western blot analyses with specific antibodies against MIF. The western blots from 3 independent experiments were analyzed densitometrically. ACTB served as the internal loading control. Results: (**a**) Differentially expressed genes were detected in *L. m.*-infected BMDM 1 and 24 h p.i.. The results were presented in MA plots. Large dots represent probe sets, which had significant differential expression (FDR < 0.05). Dot colors correspond to the direction of gene expression changes (red dots = significant upregulation; blue dots = significant downregulation). **b** At 1 h p.i., 61 probe sets and 878 probe sets at 24 h p.i. had significant differential expression in *L. m.*-infected BMDM. 25 probe sets had significant differential expression at both time points. The results were presented in a Venn diagram. **c** MIF was overexpressed in *L. m.*-infected BMDM 24 h p.i. compared to uninfected control BMDM. **d** Results of densitometric analyses of the western blots and of the corresponding Affymetrix® chip analysis confirmed MIF overexpression at the mRNA and the protein level in *L. m.*-infected BMDM 24 h p.i. compared to uninfected control BMDM. **e** 28 autophagy-related genes in *L. m.*-infected BMDM 24 h p.i. were LISA members. The edges represent known protein-protein interactions determined in the AIN. The colors correspond to the direction of the gene expression changes (red = significant upregulation; blue = significant downregulation). *L. m.*-inf. = *L. m.*-infected, n.s. = not significant, p.i. = post infection, RFU = relative fluorescence units, * = genes of glycolysis, *** *p* ≤ 0.001

After exclusion of MTOR dependency, the transcriptomes of uninfected and *L. m.*-infected BMDM 1 and 24 h p.i. were compared using Affymetrix® chips to identify autophagy-related genes in host macrophages, which induced or regulated autophagic activity (Fig. 7a). Altogether, 61 (1 h p.i.) and 878 (24 h p.i.) differentially expressed probe sets reached global significance (FDR < 0.05) (Fig. 7b, Additional file 8: Table S1 and Additional file 9: Table S2). These probe sets were mapped to 32 upregulated and 11 downregulated genes at 1 h p.i. as well as 310 genes with upregulated as well as 333 with downregulated expression 24 h p.i..

Behrends and colleagues recently published an experimental data-based network of autophagy-specific protein-protein interactions, which was called the **A**utophagy **I**nteraction **N**etwork (**AIN**) [31]. Candidate genes of *L. m.*-infected BMDM in the late infection phase (24 h p.i.) were mapped to AIN to discover interactions. Finally, 28 differentially expressed gene products from the late infection phase were used to form a highly interconnected subgraph called the ***L****eishmania***i**nfection **s**ubset of **A**IN (**LISA**, Fig. 7e and Table 1). LISA described all putative interactions between different gene products, which might play an important role in autophagy regulation during infection of BMDM with *L. m.*. Microtubule-associated proteins 1A/1B light chain 3B (*Map1lc3b* = *Lc3b*) and the AMP-activated, gamma 2 non-catalytic subunit (*Prkag2*) of 5′ AMP-activated protein kinase (AMPK) formed two central interaction hubs that were interconnected by five genes: aldolase A (*Aldoa*), fused in sarcoma (*Fus*), glyceraldehyde-3-phosphate dehydrogenase (*Gapdh*), heat shock protein 90 kDa alpha (*Hsp90aa1*), and the zinc finger CCHC domain containing 11 (*Zcchc11*) (Fig. 7e). Furthermore, *Mif*, a proinflammatory cytokine, was an upregulated component of LISA (Fig. 7e) [46]. Overexpression of MIF was also confirmed by western blot analysis at the protein level (Fig. 7c and d).

**Table 1:** Selected differentially expressed host cell genes in *L. m*.-infected BMDM

**Selected differentially expressed host cell genes in** ***L. m*** **.-infected BMDM 1 h p.i.**			
	Gene name (probe set ID)	Symbol	logFC
Additional autophagy-related genes			
	BCL2/adenovirus E1B interacting protein 3 (1422470_at)	*Bnip3*	0.417
**Selected differentially expressed host cell genes in** ***L. m*** **.-infected BMDM 24 h p.i.**			
	Gene name (probe set ID)	Symbol	logFC
LISA			
	ATP-binding cassette, sub-family E (OABP), member 1 (1416014_at)	*Abce1*	0.392
	Acyl-CoA synthetase long-chain family member 4 (1418911_s_at)	*Acsl4*	0.347
	AHA1, activator of heat shock protein ATPase homolog 1 (yeast) (1424147_at)	*Ahsa1*	0.422
	Aldolase A, fructose-bisphosphate (1433604_x_at)	*Aldoa*	0.496
	Adaptor protein complex AP-2, alpha 2 subunit (1432007_s_at)	*Ap2a2*	−0.536
	DEAD (Asp-Glu-Ala-Asp) box polypeptide 60 (1447789_x_at)	*Ddx6*	0.598
	Eukaryotic translation initiation factor 2-alpha kinase 2 (1440866_at)	*Eif2ak2*	0.533
	Enolase 2, gamma neuronal (1418829_a_at)	*Eno2*	0.673
	FUN14 domain containing 1 (1453369_a_at)	*Fundc1*	−0.357
	Fusion, derived from t(12;16) malignant liposarcoma (human) (1451285_at)	*Fus*	0.397
	Glyceraldehyde-3-phosphate dehydrogenase (1447999_x_at)	*Gapdh*	0.506
	Helicase, lymphoid specific (1417541_at)	*Hells*	0.505
	Heat shock protein 90, alpha (cytosolic), class A member 1 (1426645_at)	*Hsp90aa1*	0.543
	Potassium channel tetramerisation domain containing 12 (1434881_s_at)	*Kctd12*	−0.419
	Lactate dehydrogenase A (1419737_a_at)	*Ldha*	0.558
	Microtubule-associated protein 1 light chain 3 beta (1415929_at)	*Map1lc3b*	−0.445
	Minichromosome maintenance deficient 3 (*S. cerevisiae*) (1420028_s_at)	*Mcm3*	0.365
	Macrophage migration inhibitory factor (1416335_at)	*Mif*	0.731
	Asparaginyl-tRNA synthetase (1428666_at)	*Nars*	−0.384
	N-myc downstream regulated gene 1 (1420760_s_at)	*Ndrg1*	0.545
	Nuclear factor of activated T cells 5 (1438999_a_at)	*Nfat5*	−0.401
	Phosphoglycerate kinase 1 (1417864_at)	*Pgk1*	0.730
	Pyruvate kinase, muscle (1417308_at)	*Pkm2*	0.471
	Protein kinase, AMP-activated, gamma 2 non-catalytic subunit (1423831_at)	*Prkag2*	−0.498
	Sorbitol dehydrogenase (1438183_x_at)	*Sord*	−0.385
	Tripartite motif-containing 21 (1418077_at)	*Trim21*	0.400
	Thioredoxin reductase 1 (1421529_a_at)	*Txnrd1*	0.448
	Zinc finger, CCHC domain containing 11 (1437395_at)	*Zcchc11*	−0.477
Additional autophagy-related genes			
	BCL2/adenovirus E1B interacting protein 3 (1422470_at)	*Bnip3*	0.765
	Cathepsin E (1418989_at)	*Ctse*	−0.852
	DNA-damage regulated autophagy modulator 1 (1424524_at)	*Dram1*	0.445
	Optineurin (1435679_at)	*Optn*	0.355
	Syntaxin 5A (1449679_s_at)	*Stx5a*	−0.429
	Vacuolar protein sorting 41 (yeast) (1437901_a_at)	*Vps41*	−0.423
Glycolysis (GO accession: GO:0006096)			
	Fructose-bisphosphate aldolase A (1433604_x_at)	*Aldoa*	0.496
	Fructose-bisphosphate aldolase C (1451461_a_at)	*Aldoc*	0.636
	Enolase 2 (1418829_a_at)	*Eno2*	0.673
	Glyceraldehyde-3-phosphate dehydrogenase (1447999_x_at)	*Gapdh*	0.506
	Lactate dehydrogenase A (1419737_a_at)	*Ldha*	0.558
	Phosphoglycerate kinase 1 (1417864_at)	*Pgk1*	0.730
	Phosphoglycerate mutase 1 (1426554_a_at)	*Pgam1*	0.546
	Pyruvate kinase, muscle (1417308_at)	*Pkm2*	0.471
	Protein kinase, AMP-activated, gamma 2 non-catalytic subunit (1423831_at)	*Prkag2*	−0.498
	Triosephosphate isomerase 1 (1415918_a_at)	*Tpi1*	0.670
Immune response (GO accession: GO:0006955)			
	BCL2/adenovirus E1B interacting protein 3 (1422470_at)	*Bnip3*	0.765
	Complement component 3 (1423954_at)	*C3*	1.294
	Cell adhesion molecule 1 (1431611_a_at)	*Cadm1*	−0.424
	Chemokine (C-C motif) ligand 12 (1419282_at)	*Ccl12*	0.515
	Chemokine (C-C motif) ligand 2 (1420380_at)	*Ccl2*	0.738
	Chemokine (C-C motif) ligand 24 (1450488_at)	*Ccl24*	−0.485
	Chemokine (C-C motif) ligand 3 (1419561_at)	*Ccl3*	0.643
	Chemokine (C-C motif) ligand 4 (1421578_at)	*Ccl4*	0.710
	Chemokine (C-C motif) ligand 5 (1418126_at)	*Ccl5*	2.250
	Chemokine (C-C motif) ligand 7 (1421228_at)	*Ccl7*	0.523
	CD180 antigen (1421547_at)	*Cd180*	0.382
	CD28 antigen (1437025_at)	*Cd28*	−0.482
	CD300A antigen (1445292_at)	*Cd300a*	−0.441
	Complement component factor h (1423153_x_at)	*Cfh*	−0.783
	C-type lectin domain family 5, member a (1421366_at)	*Clec5a*	0.804
	Chemokine (C-X3-C) receptor 1 (1450020_at)	*Cx3cr1*	−0.379
	Chemokine (C-X-C motif) ligand 1 (1419209_at)	*Cxcl1*	0.808
	Chemokine (C-X-C motif) ligand 10 (1418930_at)	*Cxcl10*	2.023
	Chemokine (C-X-C motif) ligand 11 (1419697_at)	*Cxcl11*	1.205
	Chemokine (C-X-C motif) ligand 13 (1448859_at)	*Cxcl13*	0.360
	Chemokine (C-X-C motif) ligand 9 (1418652_at)	*Cxcl9*	0.698
	DEAD (Asp-Glu-Ala-Asp) box polypeptide 58 (1436562_at)	*Ddx58*	0.520
	Guanylate binding protein 1 (1420549_at)	*Gbp1*	1.349
	Guanylate binding protein 2 (1435906_x_at)	*Gbp2*	1.602
	Guanylate binding protein 3 (1418392_a_at)	*Gbp3*	0.919
	Guanylate binding protein 6 (1438676_at)	*Gbp6*	1.507
	Guanylate binding protein 7 (1434380_at)	*Gbp7*	0.459
	Histocompatibility 28 (1421596_s_at)	*H28*	0.777
	Histocompatibility 2, T region locus 24 (1422160_at)	*H2-T25*	0.847
	Intercellular adhesion molecule 1 (1424067_at)	*Icam1*	0.490
	Interferon induced with helicase C domain 1 (1426276_at)	*Ifih1*	0.664
	Immunoglobulin heavy constant mu (1427351_s_at)	*Ighm*	−0.729
	Interleukin 1 receptor antagonist (1425663_at)	*Il1rn*	0.872
	Immunity-related GTPase family M member 1 (1418825_at)	*Irgm1*	0.793
	Kelch-like 6 (Drosophila) (1437886_at)	*Klhl6*	−0.637
	Mucosa associated lymphoid tissue lymphoma translocation gene 1 (1456126_at)	*Malt1*	0.578
	Major facilitator superfamily domain containing 6 (1424463_at)	*Mfsd6*	−0.602
	Macrophage migration inhibitory factor (1416335_at)	*Mif*	0.731
	Myeloid/lymphoid or mixed-lineage leukemia 5 (1434704_at)	*Mll5*	−0.401
	Myxovirus (influenza virus) resistance 1 (1451905_a_at)	*Mx1*	1.226
	Myxovirus (influenza virus) resistance 2 (1419676_at)	*Mx2*	0.494
	NA (1416016_at)	NA	0.347
	NA (1417314_at)	NA	0.573
	NA (1424775_at)	NA	0.540
	NA (1439343_at)	NA	−0.416
	NA (1447927_at)	NA	1.137
	NA (1449009_at)	NA	1.397
	Neutrophil cytosolic factor 1 (1425609_at)	*Ncf1*	−0.491
	2′-5′ oligoadenylate synthetase 3 (1425374_at)	*Oas3*	0.545
	2′-5′ oligoadenylate synthetase-like 2 (1453196_a_at)	*Oasl2*	0.762
	ORAI calcium release-activated calcium modulator 1 (1424990_at)	*Orai1*	−0.362
	Proteasome (prosome, macropain) subunit, beta type 8 (large multifunctional peptidase 7) (1422962_a_at)	*Psmb8*	0.424
	RAB27A, member RAS oncogene family (1429123_at)	*Rab27a*	−0.393
	Radical S-adenosyl methionine domain containing 2 (1421009_at)	*Rsad2*	2.096
	Serine (or cysteine) peptidase inhibitor, clade A, member 3G (1424923_at)	*Serpina3g*	0.778
	Tumor necrosis factor (1419607_at)	*Tnf*	0.784
	Tumor necrosis factor (ligand) superfamily, member 10 (1459913_at)	*Tnfsf10*	0.495
	Tumor necrosis factor (ligand) superfamily, member 9 (1422924_at)	*Tnfsf9*	0.726
Chemokine signaling pathway (KEGG entry: mmu04062)			
	Adrenergic receptor kinase, beta 1 (1451992_at)	*Adrbk1*	−0.420
	Arrestin, beta 2 (1451987_at)	*Arrb2*	−0.443
	Chemokine (C-C motif) ligand 12 (1419282_at)	*Ccl12*	0.515
	Chemokine (C-C motif) ligand 2 (1420380_at)	*Ccl2*	0.738
	Chemokine (C-C motif) ligand 24 (1450488_at)	*Ccl24*	−0.485
	Chemokine (C-C motif) ligand 3 (1419561_at)	*Ccl3*	0.643
	Chemokine (C-C motif) ligand 4 (1421578_at)	*Ccl4*	0.710
	Chemokine (C-C motif) ligand 5 (1418126_at)	*Ccl5*	2.250
	Chemokine (C-C motif) ligand 7 (1421228_at)	*Ccl7*	0.523
	Chemokine (C-C motif) receptor 1 (1419609_at)	*Ccr1*	0.481
	C-src tyrosine kinase (1423518_at)	*Csk*	−0.387
	Chemokine (C-X3-C) receptor 1 (1450020_at)	*Cx3cr1*	−0.379
	Chemokine (C-X-C motif) ligand 1 (1419209_at)	*Cxcl1*	0.808
	Chemokine (C-X-C motif) ligand 10 (1418930_at)	*Cxcl10*	2.023
	Chemokine (C-X-C motif) ligand 11 (1419697_at)	*Cxcl11*	1.205
	Chemokine (C-X-C motif) ligand 13 (1448859_at)	*Cxcl13*	0.360
	Chemokine (C-X-C motif) ligand 9 (1418652_at)	*Cxcl9*	0.698
	Gardner-Rasheed feline sarcoma viral (Fgr) oncogene homolog (1442804_at)	*Fgr*	0.472
	Neutrophil cytosolic factor 1 (1425609_at)	*Ncf1*	−0.491
	Nuclear factor of kappa light polypeptide gene enhancer in B cells inhibitor, alpha (1448306_at)	*Nfkbia*	0.500
	Protein kinase, cAMP dependent, catalytic, beta (1420611_at)	*Prkacb*	−0.434
	Signal transducer and activator of transcription 1 (1450034_at)	*Stat1*	0.784
	Signal transducer and activator of transcription 2 (1421911_at)	*Stat2*	0.413
	Signal transducer and activator of transcription 3 (1426587_a_at)	*Stat3*	−0.371

Moreover, all significantly differentially expressed genes in *L. m.*-infected BMDM 24 h p.i. (FDR < 0.05), which were identified by Affymetrix® chip hybridization, were subjected to category enrichment analyses (Additional file 10: Figure S5) to identify additional cellular pathways and processes regulated in *L. m.*-infected BMDM and linked to increased autophagic activity. Most remarkably, upregulated genes of glycolysis (GO accession: GO:0006096), a second catabolic pathway, were enriched (Additional file 10: Figure S5 and Table 1). Also, 7 of these genes were included in LISA (Fig. 7e and Table 1). One, *Prkag2*, was even in the two central nodes in LISA (Fig. 7e) and 2 other genes, *Gapdh* and *Aldoa*, connected the nodes directly. Category enrichment analyses also suggested *L. m.-*infected BMDM 24 h p.i. had an inflammatory phenotype due to upregulated activity in a chemokine signaling pathway (KEGG entry: mmu04062) and an immune response (GO accession: GO:0006955), which included changes in *Mif* expression (Additional file 10: Figure S5 and Table 1).

**Fig. 8:**
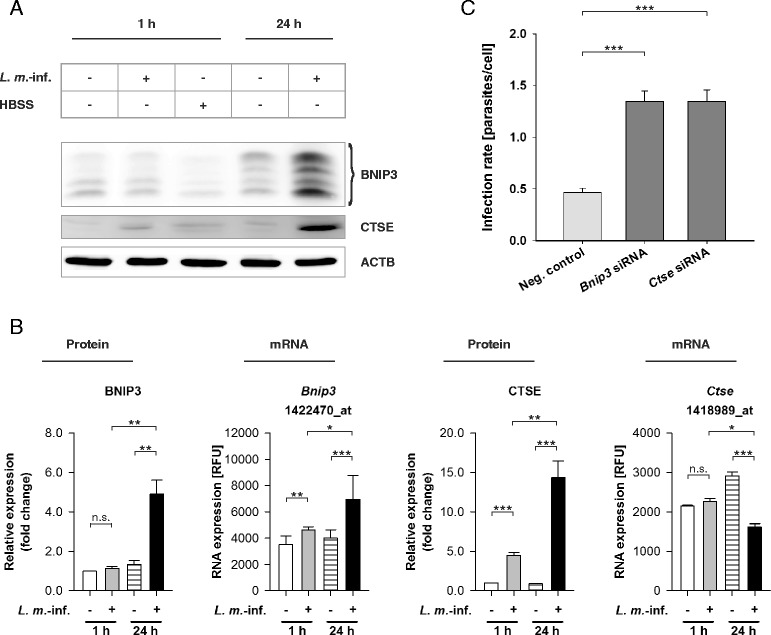
BNIP3 and CTSE transcriptomic and western blot analyses with RNAs and protein extracts from *L. m.*-infected and HBSS-starved BMDM as well as determination of the infection rates of *L. m.*-infected BMDM after BNIP3 and CTSE downregulation by RNA interference. Methods: (**a**, **b**) BMDM from BALB/c mice were infected with *L. m.* promastigotes for 1 h or 24 h. Uninfected control BMDM were incubated for the same amount of time in RPMI medium or starved for 1 h in HBSS. The Proteins were harvested and subjected to western blot analysis with specific antibodies against BNIP3 and CTSE. Western blots from 3 independent experiments were analyzed densitometrically. ACTB served as the internal loading control. Total RNA was harvested from *L. m.*-infected BMDM and uninfected control BMDM. Affymetrix® chips were hybridized with RNA samples from 2 independent experiments and analyzed densitometrically. **c** Additionally, *L. m.*-infected BMDM were transfected with specific siRNAs 20 h p.i. to downregulate the expression of BNIP3 and CTSE. *L. m.*-infected control BMDM were transfected with negative control siRNA. Infection rates were determined 48 h p.i. in 2 independent experiments. Results: (**a**) BNIP3 and CTSE were significantly overexpressed in *L. m.*-infected BMDM 24 h p.i. compared to uninfected control BMDM. **b** Densitometric analyses of western blot experiments confirmed this overexpression 24 h p.i. and showed that CTSE was also overexpressed in *L. m.*-infected BMDM 1 h p.i.. At the mRNA level, *Bnip3* was overexpressed in *L. m.*-infected BMDM 1 and 24 h p.i. and *Ctse* was downregulated in *L. m.*-infected BMDM 24 h p.i.. **c** A significant increase in the infection rates was detected in *L. m.*-infected BMDM after downregulation of protein expression of BNIP3 or CTSE compared to *L. m.*-infected BMDM transfected with negative control siRNA. *L. m.*-inf. = *L. m.*-infected, neg. control = negative control, n.s. = not significant, RFU = relative fluorescence units, * *p* ≤ 0.05, ** *p* ≤ 0.01,*** *p* ≤ 0.001

Additionally, there were 6 differentially expressed genes, including *Bnip3*, *Ctse*, damage-regulated autophagy modulator 1 (*Dram1*), optineurin (*Optn*), syntaxin 5A (*Stx5a*)*,* and vacuolar protein sorting 41 (*Vps41*), that were related to autophagy regulation but not included in the AIN (Table 1) [47–53]. Among these genes, only one, *Bnip3* (a gene contributing to MTOR-independent autophagy induction in mammalian cells [51]), was significantly upregulated at the mRNA level at both 1 and 24 h p.i. (Fig. 8b and Table 1).

In summary, expression profiling revealed that genes contributing to autophagy, glycolysis, and inflammation were differentially expressed in *L. m.*-infected BMDM 24 h p.i., and the genes were closely linked to each other.

### BNIP3 and CTSE contributed to parasite clearance in the late infection phase

*Bnip3* was the only autophagy-related gene that was significantly overexpressed at the mRNA level in Affymetrix® chip analyses in *L. m.*-infected BMDM at both 1 and 24 h p.i. (Fig. 8b and Table 1). Therefore, *Bnip3* expression was investigated at the protein level through western blot analysis. The second gene chosen for western blot analysis was *Ctse,* an aspartic protease thought to be essential for autophagic flux in macrophages [53, 54]. *Ctse* was differentially downregulated at the mRNA level 24 h p.i. compared to the respective control cells (Fig. 8b and Table 1).

A significant upregulation of BNIP3 expression at the protein level was detected 24 h p.i., but not 1 h p.i. (Fig. 8a and b), which suggests a putative inhibition of *Bnip3* translation in the early infection phase. CTSE was also significantly upregulated at the protein level 24 h p.i.; although CTSE was transcriptionally downregulated (Fig. 8a and b). A significant overexpression of CTSE was also detected 1 h p.i. in *L. m.*-infected BMDM compared to the uninfected control cells (Fig. 8a and b). However, the overexpression of CTSE was significantly more pronounced in *L. m.*-infected BMDM 24 h p.i. compared to *L. m.*-infected BMDM 1 h p.i. (Fig. 8a and b). This outcome suggested that CTSE expression was also partially inhibited in the early infection phase. Both proteins, BNIP3 and CTSE, were not differentially expressed at the protein level in HBSS-starved autophagic BMDM, which indicates that changes in these proteins are specific to *L. m.* infections.

Downregulation of BNIP3 and CTSE expression by specific siRNAs in the late infection phase (Additional file 4: Figure S4B), which is a phase normally characterized by high protein levels of BNIP3 and CTSE (Fig. 8a and b), resulted in a significant increase in the infection rates at 48 h p.i. compared to the rate of *L. m.*-infected control BMDM transfected with negative control siRNA at the same time point (Fig. 8c).

**Fig. 9:**
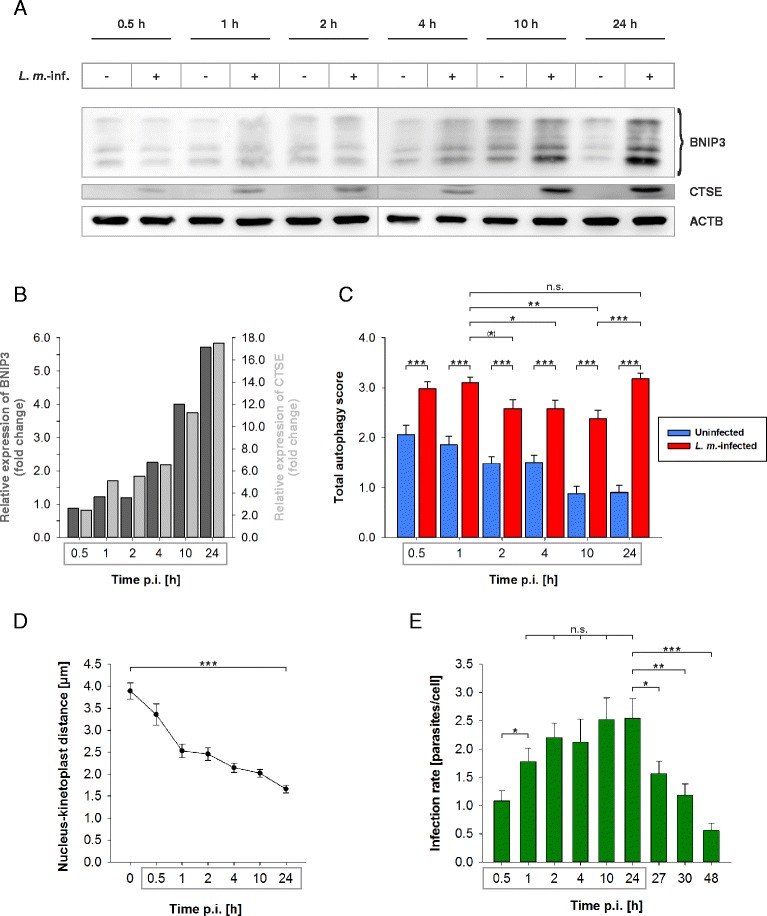
Time course experiments of BNIP3 and CTSE western blot analyses with protein extracts from *L. m.*-infected BMDM, total autophagy scores, determination of the nucleus-kinetoplast distances, and infection rates. Methods: (**a**–**e**) BMDM from BALB/c mice were infected with *L. m.* promastigotes up to 48 h p.i.. Uninfected controls were incubated for the same amount of time in RPMI medium. **a**, **b** Proteins were harvested and subjected to western blot analysis. Protein expressions of *L. m.*-infected BMDM were analyzed densitometrically. ACTB served as the internal loading control. **c** *L. m.-*infected and uninfected BMDM were subjected to TEM analyses. 50 BMDM of each sample were analyzed semiquantitatively for the grade of vacuolization (0 – 3) and the presence of MLS (+1) resulting in the total autophagy score (maximum = 4). **d**, **e** *L. m.*-infected BMDM were stained with a Diff-Quik kit to determine (**d**) the distances between the parasite nuclei and the kinetoplasts and (**e**) the infection rates for each investigated time point. Results: (**a**) BNIP3 and CTSE overexpression in *L. m.*-infected BMDM increased over time up to 24 h p.i. compared to uninfected controls. **b** This overexpression was confirmed densitometrically for BNIP3 (*dark gray columns*) and CTSE (*light gray columns*). **c** The total autophagy score was significantly increased in *L. m.*-infected BMDM (*red columns*) in all investigated samples compared to uninfected controls (*blue columns*). In *L. m.*-infected BMDM at 2 to 10 h p.i., a significant decline in the total autophagy score was detectable compared to *L. m.*-infected BMDM at 1 h p.i.. This decline might has been caused by a partial autophagy inhibition. The total autophagy score significantly rose in *L. m.*-infected BMDM at 24 h p.i. compared to 10 h p.i.. The total autophagy score declined in uninfected controls from 0.5 h until 24 h. **d** The distance between nucleus and kinteoplast shortened over time, which confirmed the differentiation of promastigotes into amastigotes (*black dots*). **e** The infection rates increased over time up to 24 h p.i. (*green columns*). Between 1 and 24 h p.i., there were no significant changes in the infection rates, which indicated an inhibition of autophagic activity. Finally, the infection rates declined from 24 h p.i. until 48 h p.i., which suggested that parasites were autophagically digested. *L. m.*-inf. = *L. m.*-infected, n.s. = not significant, p.i. = post infection, ^(^*^)^ *p* ≤ 0.1, * *p* ≤ 0.05, ** *p* ≤ 0.01, *** *p* ≤ 0.001

Furthermore, time course experiments were performed from 0.5 h p.i. up to 48 h p.i.. At the early time points (0.5 h p.i. until 4 h p.i.), there was a very low protein expression of BNIP3 and CTSE detectable compared to the protein expression at 10 and 24 h p.i. (Fig. 9a and b). Remarkably, the total autophagy scores were significantly reduced in *L. m.*-infected BMDM from 2 h p.i. until 10 h p.i. compared to the score of 1 h p.i., which suggested a partial autophagy inhibition in the early infection phase (Fig. 9c). This might be the explanation of the low expression of autophagy-related BNIP3 and CTSE at the early time points (Fig. 9a and b). The highest expression of BNIP3 and CTSE was detected at 24 h p.i.. At this time point the total autophagy score significantly rose compared to 10 h p.i., which suggested the pivotal role of BNIP3 and CTSE for induction and maintenance of autophagic activity in *L. m.*-infected BMDM (Fig. 9c).

Moreover, there were no significant changes in the infection rates from 1 to 24 h p.i. (Fig. 9e), which indicated no autophagic digestion of parasites in this time frame. This result, the low expression of BNIP3 and CTSE until 4 h p.i., and the significantly reduced total autophagy scores from 2 h p.i. until 10 h p.i. compared to 1 h p.i. supported the idea of a partial autophagy inhibition in the early infection phase. During this time the differentiation of *L. m.* promastigotes to amastigotes took place as shown by the shortening of the nucleus-kinetoplast distances (Fig. 9d). However, the infection rates of *L. m.*-infected BMDM significantly declined between 24 and 48 h p.i. (Fig. 9e), which suggests there was a high autophagic activity accompanied by high BNIP3 and CTSE expression.

In conclusion, these results suggested that BNIP3 and CTSE might have been involved in the autophagic clearance of *L. m.* parasites in the late infection phase, and the expression of these proteins was inhibited in the early infection phase when differentiation from promastigotes to amastiogtes took place.

### Fully activated autophagy in *L. m.*-infected BMDM in the late infection phase was inhibited by deacidification of the lysosomal/autophagolysosomal compartment

The amastigote drug screening assay was applied to investigate the influence of Baf A1, an autophagy inhibitor, and rapamycin, an autophagy inducer, on the infection rates of *L. m.*-infected BMDM in the late infection phase. Baf A1 treatment leads to a deacidification of the lysosomal/autophagolysosomal compartment, whereas rapamycin can induce autophagy by inhibiting MTOR kinase activity. Both compounds were added to *L. m.*-infected BMDM 24 h p.i..

No decline in the infection rates was detected in BMDM treated with Baf A1 (IC_50_ value > maximally used concentration of 0.0644 μM) (Additional file 11: Figure S6A). In contrast, a dose-dependent increase of the infection rates was detected 48 h p.i. in wells with different Baf A1 concentrations (Additional file 11: Figure S6B). The caused deacidification of the lysosomal/autophagolysosomal compartment by this compound might also have affected the activity of acidic lysosomal proteases, which contributed to parasite digestion (e.g., CTSE) [55, 56]. Interestingly, the autophagic clearance was not enhanced by rapamycin treatment (IC_50_ value > maximally used concentration of 2 μM) (Additional file 11: Figure S6A). Furthermore, no dose-dependent changes in the infection rates 48 h p.i. were detected after treatment with this autophagy inducer (Additional file 11: Figure S6B). These results indicated that maximal autophagy activity was induced in *L. m.*-infected BMDM in the late infection phase (24 h p.i.) and could not be further enhanced by rapamycin treatment.

In summary, the results of amastigote drug screening assays demonstrated that autophagic degradation of *L. m.* amastigotes took place with the highest intensity between 24 and 48 h p.i. when the infection rates declined (Fig. 9e).

### Autophagy-related miRNAs mmu-miR-101c, mmu-miR-129-5p, and mmu-miR-210-5p were differentially expressed in *L. m.*-infected BMDM in the late infection phase and directly influenced the parasite clearance

**Fig. 10:**
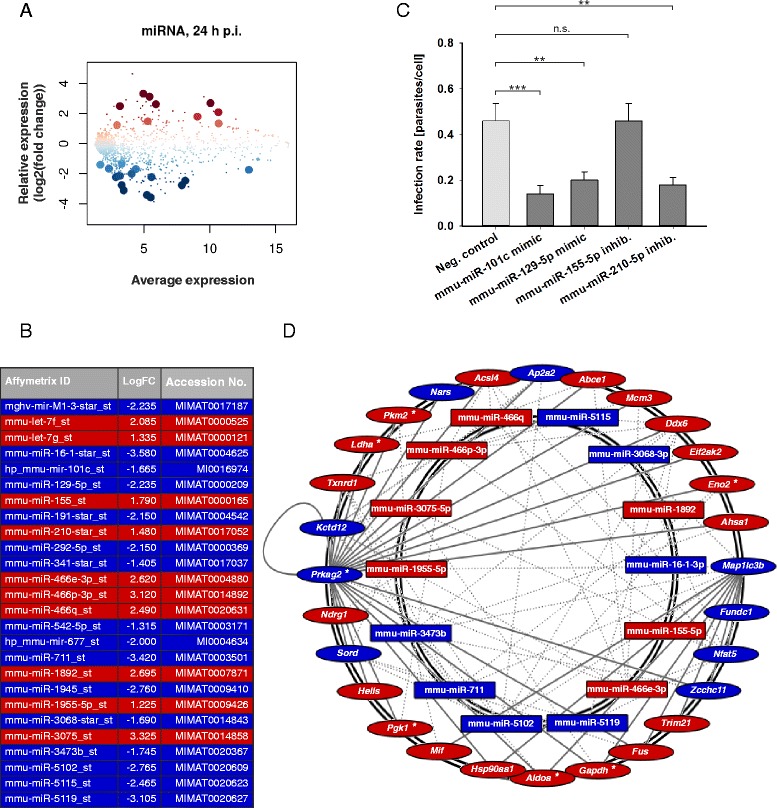
Global analysis of differentially expressed miRNAs in *L. m.*-infected BMDM, bioinformatical prediction of miRNA interactions with LISA, and infection rates of *L. m.*-infected BMDM after transfection with mmu-miR-101c or mmu-miR-129-5p mimics as well as mmu-miR-155-5p or mmu-miR-210-5p inhibitors. Methods: (**a**, **b**, **d**) BMDM from BALB/c mice were infected with *L. m.* promastigotes for 24 h. Uninfected control BMDM were incubated for the same amount of time in RPMI medium. Total RNA was harvested from *L. m.*-infected BMDM and uninfected control BMDM. Affymetrix® chips were hybridized with RNA samples from 2 independent experiments and analyzed densitometrically. Putative interactions between differentially expressed miRNAs and LISA members were predicted bioinformatically and represented in MONA-of-LISA. **c** Additionally, *L. m.*-infected BMDM were transfected with miRNA mimics or inhibitors 20 h p.i.. *L. m.*-infected control BMDM were transfected with a negative control of miRNA mimics or inhibitors. The infection rates were determined 48 h p.i. in 2 independent experiments. Results: (**a**, **b**) Differentially expressed miRNAs were detected in *L. m.*-infected BMDM 24 h p.i.. The results were presented in MA plots. Large dots represent probe sets, which were significantly and differentially expressed (FDR < 0.05). Dot and table colors correspond to the direction of miRNA expression changes (red = significant upregulation; blue = significant downregulation). **b** Table shows the significantly regulated miRNAs at 24 h p.i.. Accession No. provides detailed information about respective miRNA in (http://www.mirbase.org/). **c** A significant decrease in the infection rates was detected in *L. m.*-infected BMDM after transfection with mmu-miR-101c, mmu-miR-129-5p, and mmu-miR-210-5p compared to *L. m.*-infected BMDM transfected with a negative control of miRNA mimics or inhibitors. Transfection with mmu-miR-155-5p did not change the infection rate. **d** Putative interactions of differentially expressed miRNAs with LISA were identified and presented in MONA-of-LISA. Dotted gray lines show connections between the miRNA and the mRNAs of LISA. Solid gray lines are the connections between mRNAs already known from LISA. Node colors correspond to the direction of the gene product expression changes (red nodes = significant upregulation; blue nodes = significant downregulation). Accession No. = Accession number, Affymetrix ID = Affymetrix identifier, inhib. = inhibitor, logFC = log(fold change), neg. control = negative control, n.s. = not significant, p.i. = post infection,* = genes of glycolysis,** *p* ≤ 0.01, *** *p* ≤ 0.001

To identify additional regulatory mechanisms, involved in the autophagic clearance of *L. m.* amastigotes, the small RNA transcriptome at 24 h p.i. was analyzed with Affymetrix® chips (Fig. 10a). This analysis revealed 26 differentially expressed miRNAs (Fig. 10b). Of those miRNAs, 14 out of the 26 were bioinformatically predicted to target gene products in LISA. These putative regulatory interactions between miRNAs and target mRNAs were represented in the **m**iRNAs **o**f **n**etwork **a**nalysis of **LISA** (**MONA**-of-**LISA**) (Fig. 10d and Additional file 12: Figure S7A). Their specific roles in autophagy must be investigated in detail in future experiments.

Apart from bioinformatical predictions, published evidence has linked several differentially expressed miRNAs to autophagy-related genes (Additional file 12: Figure S7B) [57–60]. The miRNAs mmu-miR-101c, mmu-miR-129-5p, mmu-miR-155-5p, and mmu-miR-210-5p were selected for investigation of their impact on the infection rates of *L. m.*-infected BMDM. Transfection of *L. m.*-infected BMDM with miRNA mimics for mmu-miR-101c and mmu-miR-129-5p, or with an mmu-miR-210-5p inhibitor in the late infection phase, resulted in significantly decreased infection rates, which suggests that these miRNAs might influence autophagic processes directly (Fig. 10c). In contrast, transfection with an mmu-miR-155-5p inhibitor did not have any influence on the infection rate of *L. m.*-infected BMDM (Fig. 10c), which suggests this miRNA had no direct participation in autophagic digestion. However, influences of mmu-miR-155-5p on glycolysis or inflammation, which accompany the autophagic activity, must be investigated in further experiments.

Overall, a complementary analysis of mRNA and miRNA expression revealed a complex regulatory network that linked differentially expressed gene products of the glycolytic, inflammatory, and autophagic pathways. Furthermore, mmu-miR-101c, mmu-miR-129-5p, and mmu-miR-210-5p were involved in parasite clearance from BMDM.

## Discussion

Macrophages normally phagocytize and digest cellular debris and pathogens. They also stimulate lymphocytes and other immune cells to elicit an infection response. Some pathogens, including *Leishmania*, have developed the ability to manipulate macrophages to not only avoid intracellular digestion and antigen presentation but also to exploit them as host cells [6, 8].

In the present study, a new aspect of the *Leishmania*-host cell interaction in which *L. m.* amastigotes are cleared from host macrophages *in vitro* was defined. This finding was unexpected because amastigotes are known to be well-adapted to intramacrophagic conditions, and amastigotes are believed to be resistant to proteolytic digestion [6, 23].

However, BMDM generated from *Leishmania*-susceptible BALB/c mice had the capacity to eliminate cytoplasmically localized *L. m.* parasites *in vitro* after full activation of the autophagic machinery. During this clearance, the large parasites were not engulfed by autophagosomes, but they might have been penetrated by MLS from the host cell, which wrapped the parasite cytoplasm and intracellular compartments. In this process, the parasite cell membrane separated the degradation of the parasites from the host cell cytoplasm, which could prevent presentation of antigens from the dying parasites to macrophages. In this context, it has been recently shown that infection of human macrophages with apoptotic-like *L. m.* significantly reduced T-cell proliferation and parasite elimination in an autophagy-dependent way [61]. Additionally, a similar plasma-membrane-sealed digestion of the parasite has already been described during the cell death of *L. m.* promastigotes and amastigotes induced by aziridine-2,3-dicarboxylate-based cysteine protease inhibitors [11]. To the best of our knowledge, such engulfment mechanisms of microorganisms by MLS followed by autophagic clearance have never been described before. However, the nature of MLS, as well as the exact mechanism of engulfment has to be investigated in elaborate experiments in future. Furthermore, the increase in the infection rate in *L. m.*-infected BMDM after downregulation of UB expression by RNA interference suggested the participation of UB in the interaction between parasites and MLS or other autophagic membranes. Ubiquitination before degradation in autophagolysosomes was described for many other intracellar pathogens [40, 41].

The observation in the present study that autophagy induction upon *L. m.* infection was MTOR-independent contradicted a recent study that reported the inactivation of MTOR by parasite-derived protease GP63 [44]. Contrary to the experimental setup of the current study, which utilized WT BALB/c mice, Jaramillo and colleagues generated BMDM from Src homology region 2 domain-containing phosphatase-1 (SHP-1)-deficient BALB/c mice. Because SHP-1 is a GP63-activated host cell phosphatase [6], the absence of SHP-1 in BMDM might explain a higher affinity of GP63 for MTOR as well as the observed proteolytic MTOR cleavage. Moreover, in the present study, no hypophosphorylation of MTOR, which is normally an autophagy-inducing mechanism, was observed in *L. m.*-infected BMDM at the investigated time points. In contrast, hyperphosphorylation of MTOR was detected after infection with promastigotes, and there was a decreased infection rate after knocking down p-MTOR with RNA interference. This result implied that autophagolysosomal digestion might have been inhibited by unknown MTOR-dependent mechanisms in the early infection phase, to ensure a complete differentiation from promastigotes to amastigotes.

Finally, Affymetrix® chip and western blot analyses indicated that overexpressed BNIP3 and CTSE contributed to the autophagic activity in *L. m.*-infected BMDM. Downregulation of both proteins by RNA interference significantly increased the infection rates of *L. m.*-infected BMDM 48 h p.i.. These results were consistent with the literature. Several BNIP3-dependent autophagy-inducing mechanisms and a direct interaction between BNIP3 and LC3 were reported [62–64]. Involvement of the endolysosomal aspartic protease CTSE in autophagy was proposed by Kaminsky and Zhivotovsky [54] based on the observation that macrophages from CTSE-deficient mice displayed a lysosomal storage disorder [65]. This prediction was confirmed by Tsukuba and colleagues, who demonstrated an impaired autophagic proteolysis in BMDM generated from CTSE-deficient mice [53]. Furthermore, it has been shown that CTSE is predominantly expressed in antigen-presenting cells [55, 56, 66], and normally contributes to macrophage-mediated antigen presentation to T cells [67].

The significantly reduced protein expression of CTSE at 1 h p.i. compared to 24 h p.i. suggested inhibition of this autophagy-related protease occurred in the early infection phase. The simultaneous MTOR hyperphosphorylation at this time point supported the idea of a putative CTSE-p-MTOR regulation axis, which was observed in macrophages generated from CTSE-deficient mice [53]. Furthermore, the expression data of *Ctse* in the late infection phase suggested there is a large pool of *Ctse* mRNA available in BMDM for fast translation activation in the event of pathogenic invasion. The apparent decrease of mRNA levels in *L. m.*-infected BMDM support this idea, and the reduced mRNA might be necessary for fast translation of *Ctse* mRNA into the CTSE protein during infection-specific autophagy enhancement. To the best of our knowledge, the protein overexpression of CTSE was linked to an autophagy-cleared infection for the first time in the current study. The impact of this aspartic protease in the infection process has already been demonstrated in CTSE-deficient mice, which showed dramatically increased susceptibility to the bacterium *Staphylococcus aureus* [66], a bacterium, which induced autophagy in macrophages [68].

Finally, global gene expression changes in *L. m.*-infected BMDM 24 h p.i., which were detected in the present study, compared to the AIN identified by Behrends and colleagues [31] led to LISA, which consists of differentially expressed genes that might be potentially involved in regulating autophagic activity in *L. m.*-infected BMDM. Remarkably, enolase 2 (*Eno2*)*, Aldoa, Gapdh,* lactate dehydrogenase A (*Ldha*)*,* phosphoglycerate kinase 1 (*Pgk1*), and the glycolytic genes included in LISA, were upregulated in amastigote-infected BMDM, which confirms the results of a study by Rhabi and colleagues [69]. Additionally, the negative regulator of glycolysis *Prkag2* [70], which is one of the central nodes in LISA, was downregulated. Altogether, these expression data suggested that both catabolic processes, autophagy and glycolysis, were closely linked to each other in *L. m*.-infected BMDM. Additionally, increased glycolytic processes might have characterized the inflammatory phenotype of *L. m.*-infected BMDM [71], which was suggested after the category enrichment analyses. Furthermore, there was overexpression of proinflammatory MIF in *L. m.*-infected BMDM in the late infection phase. This cytokine, which was also included in LISA and originally described for its ability to inhibit random migration of macrophages *in vitro* [72], is a key player in protozoan infectious diseases [73]. Notably, *L. m.* expresses parasitic MIF orthologues to modulate the host response by binding the host cell MIF receptor cluster of differentiation 74 (CD74) [74]. The exact role of MIF in autophagy regulation is not fully understood. Both autophagy promoting and autophagy inhibiting properties have been reported for MIF [46, 75]. However, MIF-overexpressing macrophages might be potentially involved in the enhancement of *Leishmania* infection *in vivo* by recruiting new uninfected host macrophages to the infection. In this scenario, the death of some *L. m.* parasites after autophagic digestion might have enhanced the survival of other undamaged parasites, which were able to infect the newly recruited macrophages. Further experimental analyses are necessary to characterize these complex relations between autophagy, glycolysis, and inflammation in detail.

miRNAs play an important role in the interaction between host cells and different parasites [76–79]. In amastigote-loaded BMDM, 26 miRNAs were differentially expressed during the late infection phase. Such miRNAs have the potential to regulate the translation of target mRNAs influencing autophagic activity during parasite clearance [80, 81]. 14 of these 26 miRNAs were linked to LISA, which resulted in MONA-of-LISA. Additionally, 8 of these 26 miRNAs were linked to other autophagy-related genes, which were published in the literature [82–87]. Finally, miRNAs mmu-miR-101c, mmu-miR-129-5p, mmu-miR-155-5p, and mmu-miR-210-5p were selected to investigate their influence on infection rates of *L. m.*-infected BMDM. Experimental evidence reported in the literature suggested a possible impact of these 4 miRNAs on autophagic clearance of *L. m.* amastigotes from host BMDM. In addition to overexpression in *L. m.*-infected murine BMDM, miR-155 and miR-210 were also enhanced in primary human macrophages after infection with *L. m.* [88]. Therefore, miR-210 was upregulated in a hypoxia-inducible factor 1 alpha (HIF1A)-dependent manner [88, 89]. HIF1A is a transcription factor also involved in BNIP3 and MIF overexpression as well as autophagy regulation [51, 90]. Direct promotion of autophagy was also demonstrated for miR-155 during the clearance of *Mycobacteria* [59]. The miRNAs mmu-miR-101c and mmu-miR-129-5p were significantly downregulated in our experiments during the late infection phase. Inhibition of autophagy by miR-101 targeting autophagy related 4 (ATG4) protein, which is an essential protein for LC3 processing, was recently reported [57]. miR-129 might influence CTSE expression indirectly by binding the 3-untranslated region (3**′**-UTR) of specificity protein 1 (SP1) [60]. SP1 is a transcription factor that positively regulates CTSE expression in macrophages [91]. A significant downregulation of SP1-expression was observed after transfection of HeLa cells with exogenous miR-129-5p [60]. However, the results in the present study showed that 3 out of these 4 investigated miRNAs had a significant impact on the antiparasitic activity in *L. m.*-infected BMDM. Transfection of *L. m.*-infected BMDM with an mmu-miR-210-5p inhibitor as well as with mmu-miR-101c and mmu-miR-129 mimics significantly decreased the infection rates of these cells. Only the transfection of *L. m.*-infected BMDM with an mmu-miR-155-5p inhibitor did not change the infection rate. However, miR-155 was frequently overexpressed in activated macrophages [92, 93]. Therefore, the upregulation of glycolytic and inflammatory genes and the expression pattern of mmu-miR-155-5p in our experiments suggested *L. m.*-infected autophagic macrophages in the late infection phase had an inflammatory phenotype. In contrast to the expected decrease of infection rates from transfection of *L. m.*-infected BMDM with an mmu-miR-210-5p inhibitor and an mmu-miR-129-5p mimic, the infection rates also decreased after treatment with an mmu-miR-101c mimic. This result contradicted the previous observation of autophagy inhibition by targeting ATG4 with miR-101c [57]. In summary, there is a strong experimental evidence in the present study that miRNAs might contribute to autophagic clearance of *L. m.* from BMDM, but further functional analyses are necessary to clarify the exact contributions of each identified miRNA to apply that knowledge in future miRNA-based antileishmanial therapeutic approaches [94]. The absence of RNA interference in the “Old World” *Leishmania* species (e.g., *L. m.*, *Leishmania donovani*) [95], and the presence of this mechanism in host cells are advantages for avoiding unwanted side effects during miRNA-based therapies.

In conclusion, beside the bilateral interactions between parasites and host macrophages, it will be important in the future to understand the more complex interplay of *L. m.*-infected macrophages with T helper (Th)-cells. These cells secrete anti-leishmanial Th1 cytokines (e.g., autophagy-inducing interferon gamma) and pro-leishmanial Th2 cytokines (e.g., autophagy-inhibiting interleukin-4) after antigen-presentation by DCs or macrophages [4, 96]. The susceptibility for *L. m.* infections of BALB/c mice with a strong pro-leishmanial Th2 response, and the resistance to *L. m.* infections of C57BL/6 mice with a strong anti-leishmanial Th1 response [23] suggest there is an impact of Th-cell-secreted cytokines on autophagy regulation in host macrophages *in vivo*. This scenario is probably because macrophages generated from both mouse strains can clear amastigotes *in vitro* (Additional file 13: Figure S8). A similar complex interaction of macrophages with Th1/Th2-specific cytokines was already reported for *Mycobacterium tuberculosis* infection-induced autophagy [8].

## Conclusions

**Fig. 11:**
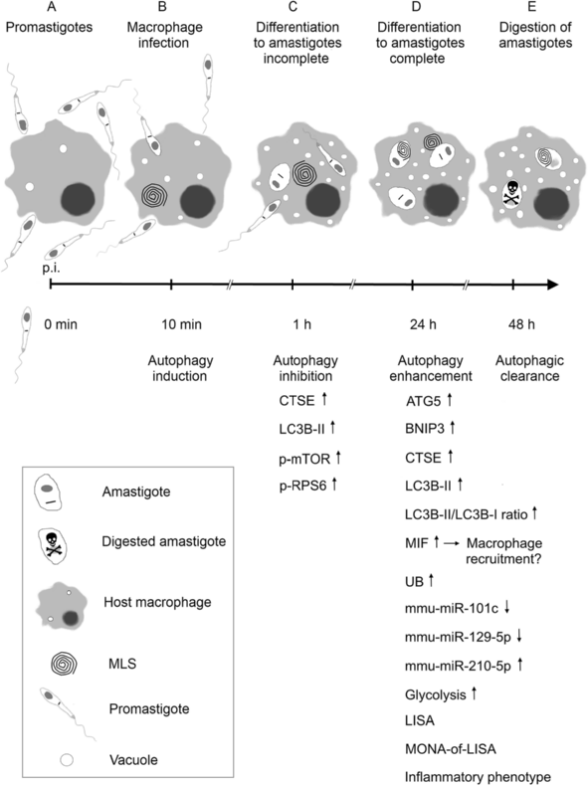
Schematic summary of results. **a** 0 h p.i.: macrophages were infected with *L. m.* promastigotes. **b** 10 min p.i.: promastigotes attached to macrophages and were phagocytosed by the cells. **c** 1 h p.i. (early infection phase): promastigotes differentiated intracellularly into amastigotes. At this point, the differentiation was not complete. Hyperphosphorylation of MTOR and RPS6 suggested autophagy inhibition. **d** 24 h p.i. (late infection phase): amastigote differentiation was completed. ATG5, BNIP3, CTSE, MIF, UB, and miRNAs mmu-miR-155-5p and mmu-miR-210-5p, were overexpressed. Expression of miRNAs mmu-miR-101c and mmu-miR-129-5p was downregulated. The LC3B-II/LC3B-I ratio was elevated and suggested an increased autophagic flux. Glycolytic genes were upregulated. Overexpressed MIF might have attracted new uninfected host macrophages. Putative regulatory mechanisms at the RNA level were identified, which were summarized in LISA and MONA-of-LISA. Additionally, inflammatory functions (e.g., the immune response and chemokine signaling pathway) were upregulated, which indicates *L. m.*-infected BMDM had an inflammatory phenotype. **e** 48 h p.i.: amastigotes were autophagically digested, which resulted in a decline in the infection rate. p.i. = post infection

As outlined in Fig. 11, the experimental results of the current study clearly demonstrated that (1) autophagy was induced in *L. m.*-infected BMDM in the early and in the late infection phase; (2) autophagy induction was MTOR-independent; (3) autophagy was dependent on p-MTOR inhibition in host macrophages in the early infection phase before differentiation from promastigotes to amastigotes was completed; (4) MLS were a prerequisite for autophagic clearance of *L. m.* amastigotes; and (5) *L. m.* amastigotes were subsequently cleared from host macrophages by the autophagic machinery in the late infection phase. Furthermore, several molecules regulating autophagy activity in the late infection phase were identified. Direct influences on parasitic clearance were shown for (1) the proteins BNIP3 and CTSE, and (2) the miRNAs mmu-miR-101c, mmu-miR-129-5p, and mmu-miR-210-5p. Moreover, additional bioinformatic analyses revealed the complex RNA network MONA-of-LISA, which putatively regulates the autophagic activity of *L. m.*-infected BMDM and consists of differentially expressed autophagy-, glycolysis-, and inflammation-related mRNAs and miRNAs. The fact that host macrophages could clear *L. m.* infections from host macrophages by autophagic digestion, and the identification of putative regulatory mechanisms of autophagic activity, might be key information for developing new anti-leishmanial therapies.

## Additional files

Additional file 1: Figure S1. Ultrastructural investigation of *L. m.* promastigotes with TEM. Method: *L. m.* promastigotes were harvested form blood agar plates and subjected to TEM analyses. Result: *L. m.* promastigotes are lancet-shaped and externally flagellated with a length of approximately 10 μm. F = flagellum, FP = flagellar pocket, K = kinetoplast, kDNA = kinetoplastid DNA, L = lysosome-like vacuole, M = mitochondrion, NP = nucleus of parasite. Additional file 2: Figure S2. Investigation of cytotoxic effects of specific siRNAs and miRNA mimics or inhibitors on BMDM. Methods: BMDM from BALB/c mice were transfected with specific siRNAs, miRNA mimics or inhibitors, and negative control RNA. Untreated BMDM served as control BMDM. Cytotoxicity was tested by alamarBlue® cytotoxicity assay. Results: Transfection of BMDM with used siRNAs, miRNA mimics or inhibitors, as well as negative control RNA had no cytotoxic effects on BMDM compared to untreated BMDM. Inhib. = inhibitor, neg. control = negative control, OD = optical density. Additional file 3: Figure S3. Ultrastructural investigation of autophagy induction with TEM and autophagy assessment in rapamycin-treated and HBSS-starved BMDM. Methods: BMDM from BALB/c mice were treated with (A – D, I, J) 500 nM rapamycin for 1 h or starved in (E – H, I, J) HBSS for 1 h. All BMDM were subjected to TEM analyses. (I, J) 50 BMDM of each sample were analyzed semiquantitatively for the grade of vacuolization (0 – 3) and the presence of MLS (+1), which resulted in a total autophagy score (maximum = 4). The total autophagy score and frequency of MLS were calculated. Results: Autophagic phenotypes characterized by (A, B, E, F) a strong vacuolization, (C, G) presence of MLS, and (D, H) of autophagosomes were detected in rapamycin-treated and HBSS-starved BMDM. Details in images C, D, G, H were magnified from images A, B, E, F from sections of BMDM (red squares = MLS in C and G, red circles = autophagosomes in D and H). (I, J) The total autophagy score and the frequency of MLS were not significantly different in *L. m.*-infected BMDM 1 and 24 h p.i. compared to autophagy-induced BMDM with rapamycin or HBSS. The total autophagy score and the frequency of MLS were significantly increased in *L. m.*-infected BMDM 1 and 24 h p.i. compared to uninfected control BMDM. N = nucleus of macrophage, n.s. = not significant, *** p ≤ 0.001. Additional file 4: Figure S4. Western blot analyses of downregulation of protein expression of ATG5, BNIP3, CTSE, MTOR, p-MTOR, and UB in *L. m.*-infected BMDM by RNA interference. Methods: (A) BMDM from BALB/c mice were transfected with *Atg5*-, *Mtor*-, or *Ub*-specific siRNAs 4 h prior to infection to downregulate the expression of proteins. The cells were also infected with *L. m. *promastigotes. *L. m.*-infected control BMDM were transfected with negative control siRNA. Protein from *L. m.*-infected BMDM was harvested after 2 h, 8 h, and 20 h p.i. (6, 12, and 24 h after transfection) and subjected to western blot analyses. ACTB served as the internal loading control. (B) BMDM from BALB/c mice were infected with *L. m.* promastigotes 20 h p.i.. *L. m.*-infected BMDM were transfected with *Bnip3*- or *Ctse*-specific siRNAs to downregulate the expression of proteins. *L. m.*-infected control BMDM were transfected with negative control siRNA. Protein from *L. m.*-infected BMDM was harvested after 26 h, 32 h, and 44 h p.i. (6, 12, and 24 h after transfection) and subjected to western blot analyses. ACTB served as the internal loading control. Results: (A, B) Western blot analyses of transfected *L. m.*-infected BMDM showed a specific downregulation of protein levels compared to the BMDM transfected with negative control siRNA. Neg. control = negative control, p.i. = post infection. Additional file 5:
**Video 1.** Localization of MLS in a representative *L. m.* amastigote in BMDM 24 h p.i.. Electron tomography displays extracellular (red frame) and intracellular (blue frame) localization of MLS. Magnification 10000 × . Additional file 6:
**Video 2.** Close association of extracellular MLS and parasite membrane. Magnification of red frame from Video 1. Detailed electron tomography of close association between extracellular MLS and amastigote membrane (red arrow). Magnification 25000 × . Additional file 7:
**Video 3.** Interaction between intracellular MLS and parasitic microtubules. Magnification of blue frame from Video 1. Detailed electron tomography of interaction between intracellular MLS and amastigote microtubules (blue arrows). Magnification 25000 × . Additional file 8: Table S1. Differentially expressed genes between uninfected and *L. m.-*infected BMDM 1 h p.i. Additional file 9: Table S2. Differentially expressed genes between uninfected and *L. m.-*infected BMDM 24 h p.i. Additional file 10: Figure S5. Category enrichment analyses of differentially expressed genes in *L. m.*-infected BMDM 24 h p.i.. Methods: Total RNA was harvested from *L. m.*-infected BMDM 24 h p.i. and uninfected control BMDM. Affymetrix® chips were hybridized with RNA samples from 2 independent experiments. Genes displaying globally significant expression changes (FDR < 0.05) were subjected to category enrichment analyses. Results: Category enrichment analyses showed several significantly regulated categories (FDR < 0.05). Beside autophagy, glycolysis, a second catabolic pathway, was significantly regulated. The enrichment analyses also suggested an inflammatory phenotype of *L. m.-*infected BMDM 24 h p.i., e.g. by demonstration of regulated immune response and chemokine signaling pathway. Radar line diagram shows the number of differentially expressed genes in the respective category. Colors indicate the database of enriched categories (green = Gene Ontology database; orange = KEGG database). Additional file 11: Figure S6. Investigation of influences of autophagy induction and inhibition on infection rates in the late infection phase. Methods: (A, B) The amastigote drug screening assay was applied to investigate the influence of an autophagy inhibitor, Baf A1, and of an autophagy inducer, rapamycin, on the infection rate of *L. m.*-infected BMDM in the late infection phase. BMDM were infected with luciferase-transgenic *L. m.* promastigotes. Baf A1 or rapamycin were added to BMDM 24 h p.i. when amastigote differentiation was completed and autophagy was fully induced. Then, BMDM were incubated for further 24 h. Untreated *L. m.*-infected BMDM were incubated for the same amount of time in RPMI. After cell lysis with a luciferin-containing buffer, the IC_50_-values for Baf A1 and rapamycin against *L. m.* amastigotes and BMDM were determined by the resulting luminescence. Results: (A) There were no IC_50_-values determinable. Thus, no decline of the infection rate by Baf A1 and rapamycin was detectable in BMDM. (B) Measurement of luminescence showed a dose-dependent increase of the infection rate with increasing Baf A1 concentrations applied to inhibit the lysosomal/autophagosomal acidification. Interestingly, no further enhancement of autophagic clearance with rapamycin was possible. Baf A1 = Bafilomycin A1, IC_50_ = half maximal inhibitory concentration, LU = luminescence unit, n.s. = not significant, ^(^*^)^ p ≤ 0.1, * p ≤ 0.05, ^+^ = maximally used concentration of compound. Additional file 12: Figure S7. Analyses of interactions of miRNAs with target genes in *L. m.*-infected BMDM 24 h p.i.. Methods: (A) Total RNA was harvested from *L. m.*-infected BMDM 24 h p.i. and uninfected control BMDM. Affymetrix® chips were hybridized with RNA samples from 2 independent experiments. Significantly differentially expressed miRNAs were predicted bioinformatically to target genes of LISA. (B) Apart from the bioinformatical predictions, published evidence has linked several differentially expressed miRNAs to further autophagy-related genes. Results: (A) Affymetrix® chip analyses revealed 26 differentially expressed miRNAs. Thereof, 14 of these 26 were bioinformatically predicted to target gene products in LISA, which resulted in MONA-of-LISA. (B) Interactions between miRNAs and autophagy-related target genes, which have been previously reported. Letters from a – l are linked with the corresponding literature (a = [82], b = [83], c = [84], d = [63], e = [58], f = [85], g = [89], h = [88], i = [86], j = [59], k = [60], l = [87]). miRNA/mRNA names are linked with databases for additional information. Colors indicate direction of regulation. (Red = upregulation; blue = downregulation). * = miRNA entry on (http://www.mirbase.org/) was removed. Additional file 13: Figure S8. Analysis of infection rates in *L. m.*-infected BMDM generated from BALB/c or C57BL/6 mice during time course experiments. Methods: BMDM either generated from BALB/c or C57BL/6 mice were infected with *L. m.* promastigotes. In a time frame from 0.5 to 48 h, *L. m.*-infected BMDM were harvested and the infection rates of *L. m.*-infected BMDM were determined by light microscopy after staining with Diff-Quik kit for each investigated time point. For each time point 50 individual BMDM were analyzed. Results: *L. m.*-infected BMDM generated from *Leishmania*-resistant C57BL/6 and *Leishmania*-susceptible BALB/c mice showed similar infection courses. In *L. m.*-infected BMDM generated from C57BL/6 mice, the infection rates were lower and declined faster compared to *L. m.*-infected BMDM generated from BALB/c mice. p.i. = post infection.

## Abbreviations

*Actb*, ACTB: β-Actin
AIN: Autophagy interaction network
*Aldoa*: Aldolase A
AMPK: 5′ AMP-activated protein kinase
ATG4: Autophagy related 4
*Atg5*, ATG5: Autophagy related 5
Baf A1: Bafilomycin A1
BMDM: Bone marrow-derived macrophages
*Bnip3*, BNIP3: BCL2/adenovirus E1B 19 kDa protein-interacting protein 3
CD74: Cluster of differentiation 74
*Ctse*, CTSE: Cathepsin E
DC: Dendritic cell
*Dram1*: Damage-regulated autophagy modulator 1
DMSO: Dimethyl sulfoxide
*Eno2*: Enolase 2
F: Flagellum
FCS: Fetal calf serum
FDR: False discovery rate
FP: Flagellar pocket
*Fus*: Fused in sarcoma
*Gapdh*: Glyceraldehyde-3-phosphate dehydrogenase
GEO: Gene expression omnibus
GO: Gene ontology
GSEA: Gene set enrichment analysis
GP63: Glycoprotein 63
HBSS: Hank’s balanced salt solution
Hepes: 4-(2-hydroxyethyl)-1-piperazineethanesulfonic acid
HGNC: HUGO Genome Nomenclature Committee
HIF1A: Hypoxia-inducible factor 1 alpha
HOMEM: Modified minimal Eagle’s medium
HRP: Horse radish peroxidase
*Hsp90aa1*: Heat shock protein 90 kDa alpha
HUGO: Human Genome Organization
IC_50_: Half maximal inhibitory concentration
K: Kinetoplast
kDNA: Kinetoplastid DNA
KEGG: Kyoto encyclopedia of genes and genomes
LC3B: Microtubule-associated proteins 1A/1B light chain 3B
*Ldha*: Lactate dehydrogenase A
LISA: *Leishmania* infection subset of AIN
LU: Luminescence unit
*L. m.*: *Leishmania major*
*L. m.*-inf.: *L. m.*-infected
L: Lysosome-like vacuole
M: Mitochondrion
*Map1lc3b*: Microtubule-associated proteins 1A/1B light chain 3B
MGI: Mouse genome informatics
*Mif*, MIF: Macrophage migration inhibitory factor
miR, miRNA: microRNA
MLS: Myelin-like structures
MONA: miRNAs of network analysis
MONA-of-LISA: miRNAs of network analysis of *Leishmania* infection subset of AIN
mRNA: Messenger RNA
*Mtor*, MTOR: Mechanistic target of rapamycin
M-CSF: Macrophage colony-stimulating factor
N: Nucleus of macrophage
Neg. control: Negative control
NIH: National Institutes of Health
NP: Nucleus of parasite
n.s.: Not significant
OD: Optical density
*Optn*: Optineurin
P: Parasite
PBS: Phophate-buffered saline
PE: Phosphatidylethanolamine
p.i.: Post infection
*Pgk1*: Phosphoglycerate kinase 1
*Prkag2*: Protein kinase, AMP-activated, gamma 2 non-catalytic subunit
p-MTOR: Phosphorylated MTOR
p-RPS6: Phosphorylated RPS6
RFU: Relative fluorescence units
RIN: RNA integrity number
RMA: Robust multi-array average
RPMI: Roswell Park Memorial Institute Medium 1640
*Rps6*, RPS6: Ribosomal protein S6
SDS: Sodium dodecyl sulfate
SDS-PAGE: Sodium dodecyl sulfate polyacrylamide gel electrophoresis
SHP-1: Src homology region 2 domain-containing phosphatase-1
siRNA: Small interfering RNA
SP1: Specificity protein 1
*Stx5a*: Syntaxin 5A
TEM: Transmission electron microscopy
Th: T helper
*Ub*, UB: Ubiquitin
*Vps41*: Vacuolar protein sorting 41
WHO: World Health Organization
WT: Wild-type
*Zcchc11*: Zinc finger, CCHC domain containing 11
3′-UTR: 3-untranslated region
