# Supplementary figures and images for: Autophagic digestion of Leishmania major by host macrophages is associated with differential expression of BNIP3, CTSE, and the miRNAs miR-101c, miR-129, and miR-210
Source: Parasit Vectors. 2015 Jul 31;8:404. doi: 10.1186/s13071-015-0974-3 (PMC4521392; doi:10.1186/s13071-015-0974-3)

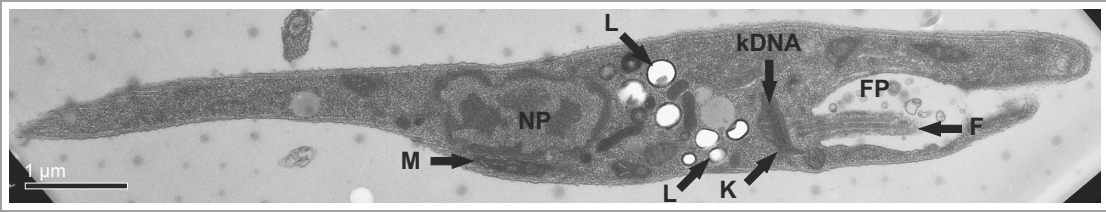

Supplement: Additional file 1: Figure S1. — Ultrastructural investigation of L. m. promastigotes with TEM. Method: L. m. promastigotes were harvested form blood agar plates and subjected to TEM analyses. Result: L. m. promastigotes are lancet-shaped and externally flagellated with a length of approximately 10 μm. F = flagellum, FP = flagellar pocket, K = kinetoplast, kDNA = kinetoplastid DNA, L = lysosome-like vacuole, M = mitochondrion, NP = nucleus of parasite. [file 13071_2015_974_MOESM1_ESM.pdf]

**Cytotoxicity against BMDM**

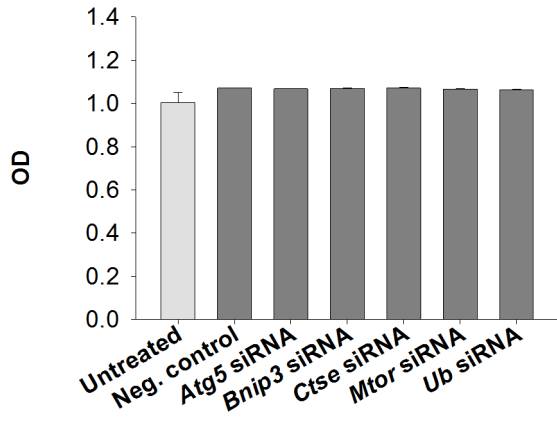

**Cytotoxicity against BMDM**

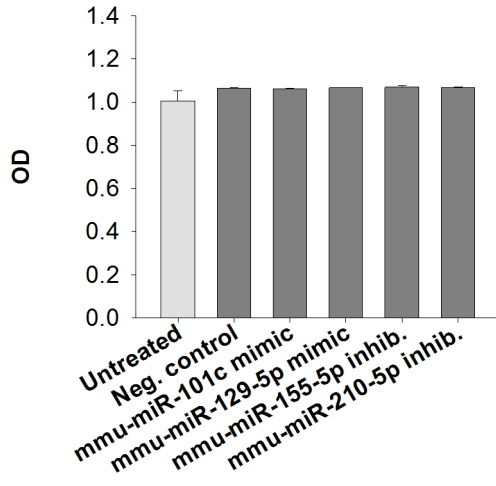

Supplement: Additional file 2: Figure S2. — Investigation of cytotoxic effects of specific siRNAs and miRNA mimics or inhibitors on BMDM. Methods: BMDM from BALB/c mice were transfected with specific siRNAs, miRNA mimics or inhibitors, and negative control RNA. Untreated BMDM served as control BMDM. Cytotoxicity was tested by alamarBlue® cytotoxicity assay. Results: Transfection of BMDM with used siRNAs, miRNA mimics or inhibitors, as well as negative control RNA had no cytotoxic effects on BMDM compared to untreated BMDM. Inhib. = inhibitor, neg. control = negative control, OD = optical density. [file 13071_2015_974_MOESM2_ESM.pdf]

## Rapamycin 1 h

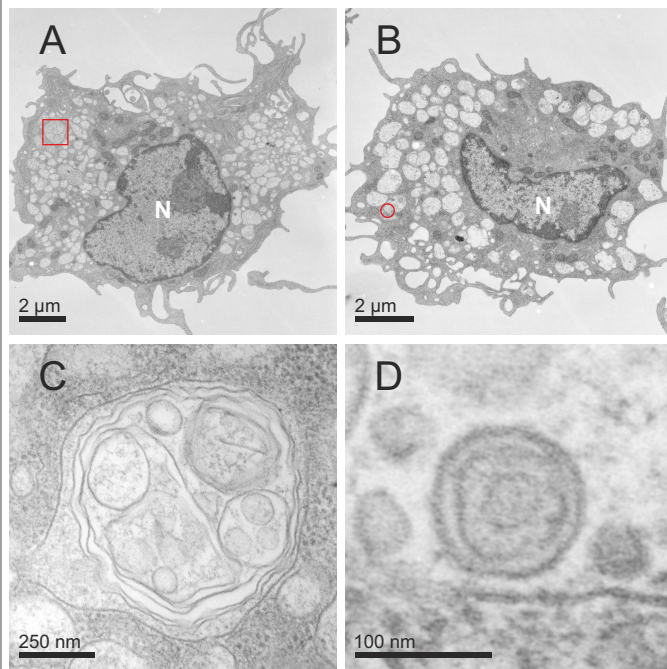

## HBSS 1 h

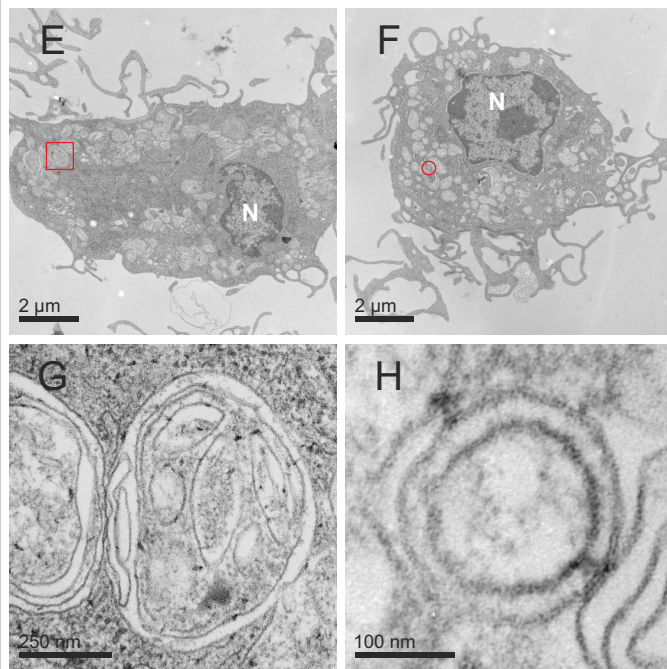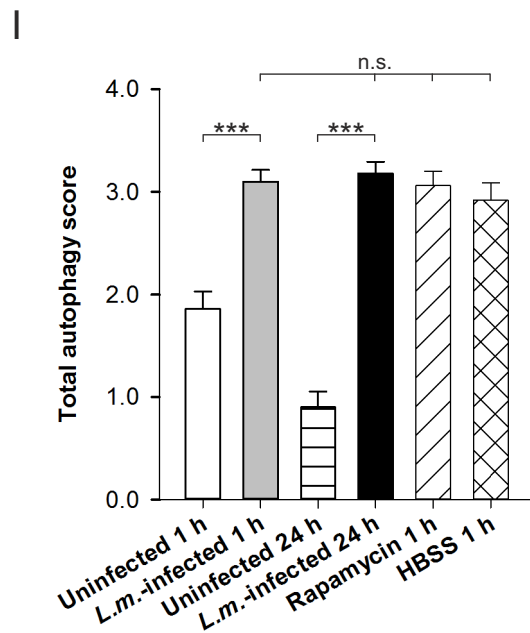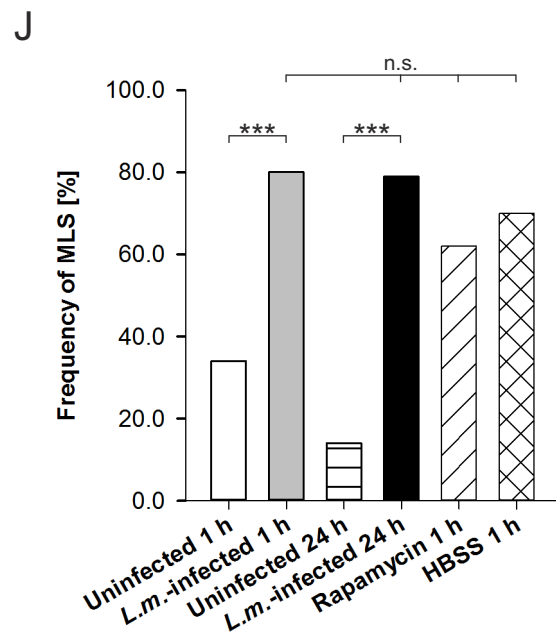

Supplement: Additional file 3: Figure S3. — Ultrastructural investigation of autophagy induction with TEM and autophagy assessment in rapamycin-treated and HBSS-starved BMDM. Methods: BMDM from BALB/c mice were treated with (A – D, I, J) 500 nM rapamycin for 1 h or starved in (E – H, I, J) HBSS for 1 h. All BMDM were subjected to TEM analyses. (I, J) 50 BMDM of each sample were analyzed semiquantitatively for the grade of vacuolization (0 – 3) and the presence of MLS (+1), which resulted in a total autophagy score (maximum = 4). The total autophagy score and frequency of MLS were calculated. Results: Autophagic phenotypes characterized by (A, B, E, F) a strong vacuolization, (C, G) presence of MLS, and (D, H) of autophagosomes were detected in rapamycin-treated and HBSS-starved BMDM. Details in images C, D, G, H were magnified from images A, B, E, F from sections of BMDM (red squares = MLS in C and G, red circles = autophagosomes in D and H). (I, J) The total autophagy score and the frequency of MLS were not significantly different in L. m.-infected BMDM 1 and 24 h p.i. compared to autophagy-induced BMDM with rapamycin or HBSS. The total autophagy score and the frequency of MLS were significantly increased in L. m.-infected BMDM 1 and 24 h p.i. compared to uninfected control BMDM. N = nucleus of macrophage, n.s. = not significant, *** p ≤ 0.001. [file 13071_2015_974_MOESM3_ESM.pdf]

A

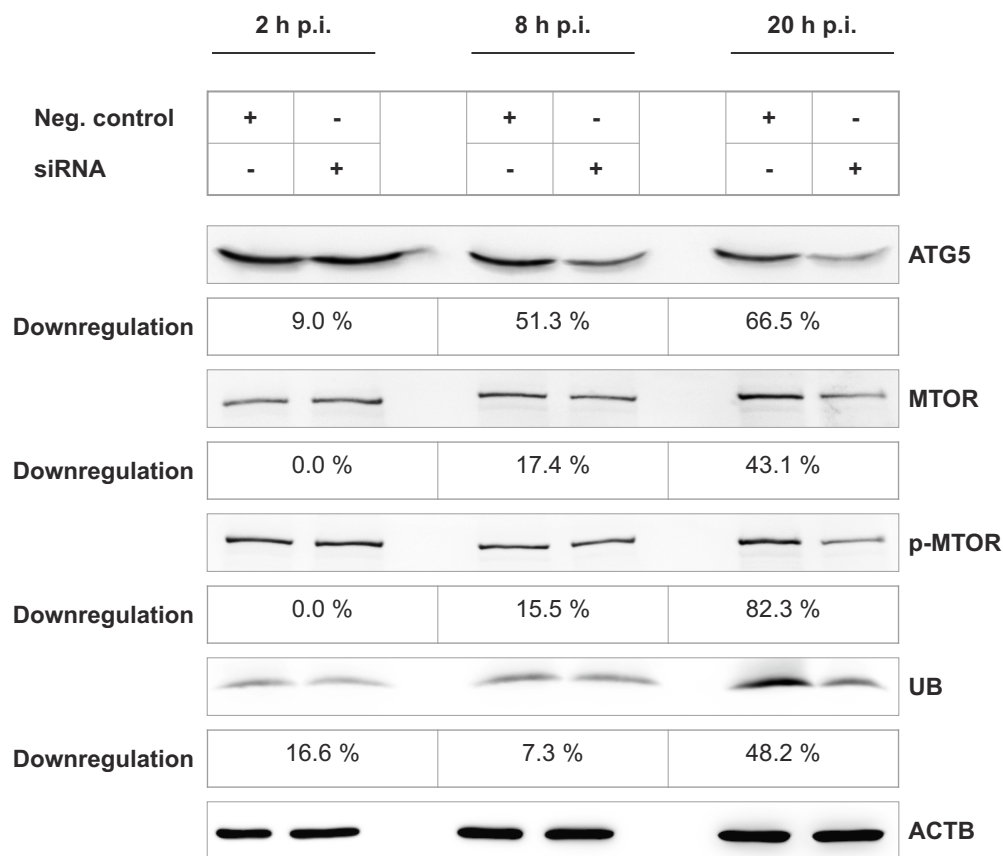

B

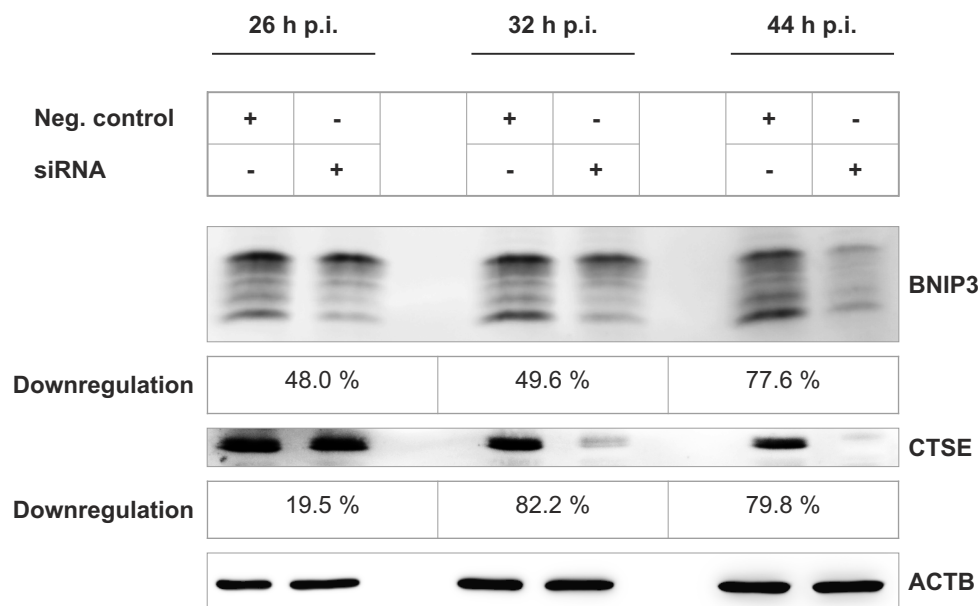

Supplement: Additional file 4: Figure S4. — Western blot analyses of downregulation of protein expression of ATG5, BNIP3, CTSE, MTOR, p-MTOR, and UB in L. m.-infected BMDM by RNA interference. Methods: (A) BMDM from BALB/c mice were transfected with Atg5-, Mtor-, or Ub-specific siRNAs 4 h prior to infection to downregulate the expression of proteins. The cells were also infected with L. m. promastigotes. L. m.-infected control BMDM were transfected with negative control siRNA. Protein from L. m.-infected BMDM was harvested after 2 h, 8 h, and 20 h p.i. (6, 12, and 24 h after transfection) and subjected to western blot analyses. ACTB served as the internal loading control. (B) BMDM from BALB/c mice were infected with L. m. promastigotes 20 h p.i.. L. m.-infected BMDM were transfected with Bnip3- or Ctse-specific siRNAs to downregulate the expression of proteins. L. m.-infected control BMDM were transfected with negative control siRNA. Protein from L. m.-infected BMDM was harvested after 26 h, 32 h, and 44 h p.i. (6, 12, and 24 h after transfection) and subjected to western blot analyses. ACTB served as the internal loading control. Results: (A, B) Western blot analyses of transfected L. m.-infected BMDM showed a specific downregulation of protein levels compared to the BMDM transfected with negative control siRNA. Neg. control = negative control, p.i. = post infection. [file 13071_2015_974_MOESM4_ESM.pdf]

# Category enrichment analyses

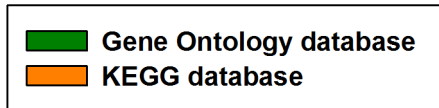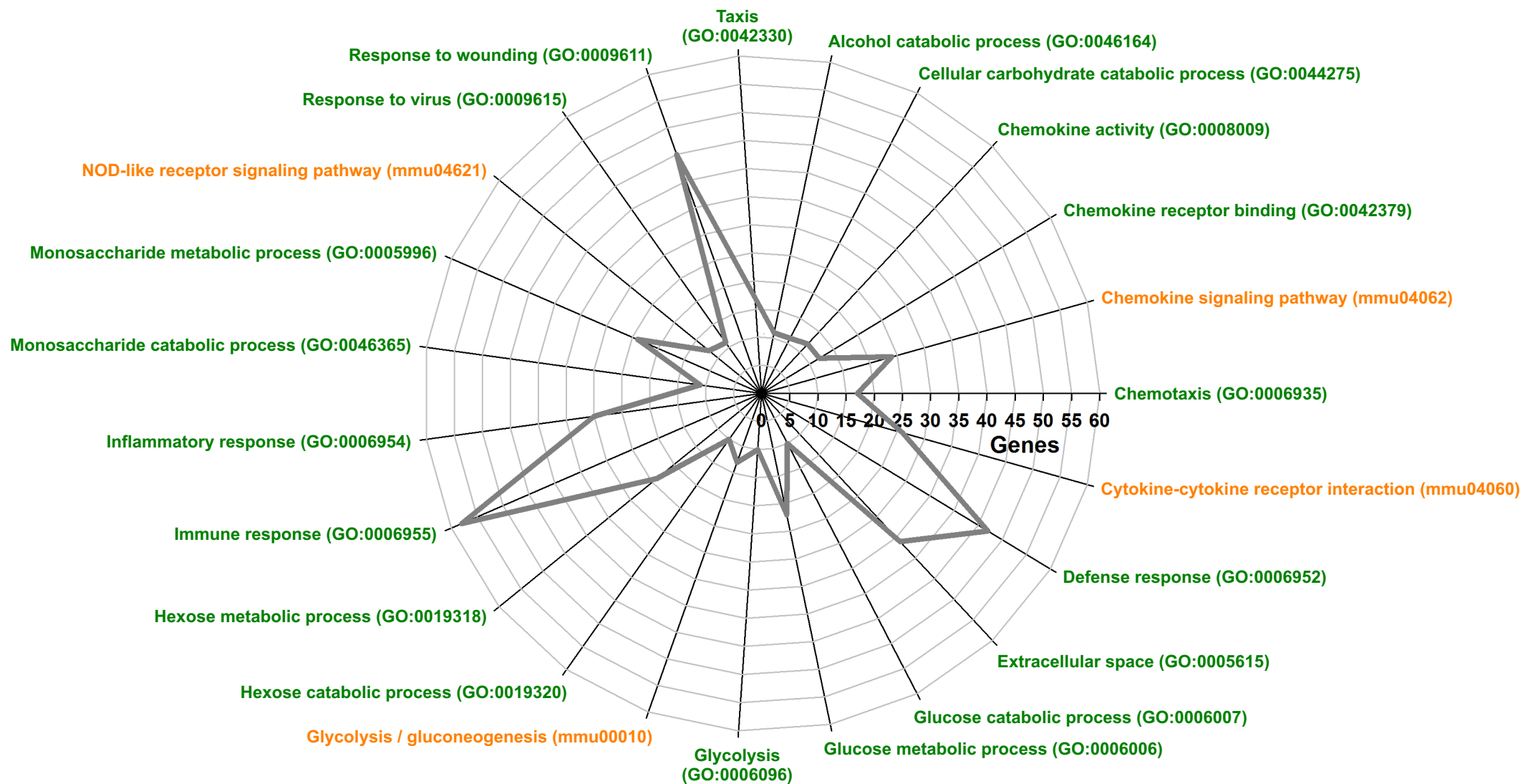

Supplement: Additional file 10: Figure S5. — Category enrichment analyses of differentially expressed genes in L. m.-infected BMDM 24 h p.i.. Methods: Total RNA was harvested from L. m.-infected BMDM 24 h p.i. and uninfected control BMDM. Affymetrix® chips were hybridized with RNA samples from 2 independent experiments. Genes displaying globally significant expression changes (FDR < 0.05) were subjected to category enrichment analyses. Results: Category enrichment analyses showed several significantly regulated categories (FDR < 0.05). Beside autophagy, glycolysis, a second catabolic pathway, was significantly regulated. The enrichment analyses also suggested an inflammatory phenotype of L. m.-infected BMDM 24 h p.i., e.g. by demonstration of regulated immune response and chemokine signaling pathway. Radar line diagram shows the number of differentially expressed genes in the respective category. Colors indicate the database of enriched categories (green = Gene Ontology database; orange = KEGG database). [file 13071_2015_974_MOESM10_ESM.pdf]

A

| Compound  | IC <sub>50</sub> -values [ $\mu$ M] |                       |
|-----------|-------------------------------------|-----------------------|
|           | Amastigotes                         | BMDM                  |
| Baf A1    | > 0.0644 <sup>+</sup>               | > 0.0644 <sup>+</sup> |
| Rapamycin | > 2.0 <sup>+</sup>                  | > 2.0 <sup>+</sup>    |

B

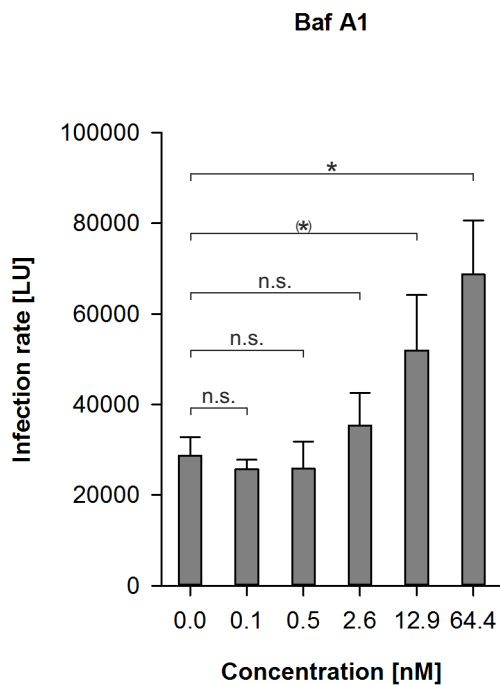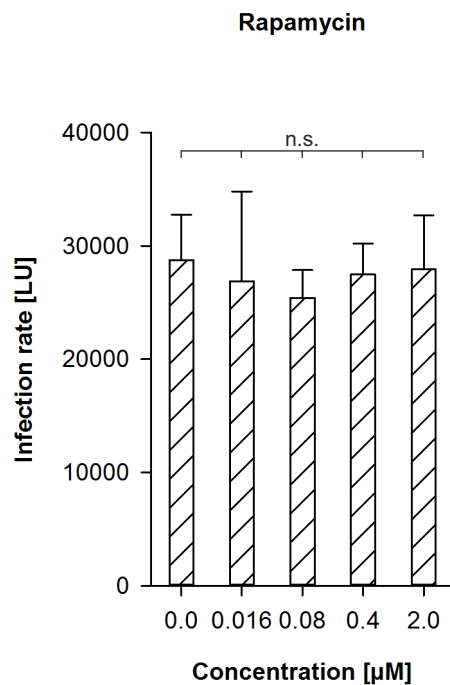

Supplement: Additional file 11: Figure S6. — Investigation of influences of autophagy induction and inhibition on infection rates in the late infection phase. Methods: (A, B) The amastigote drug screening assay was applied to investigate the influence of an autophagy inhibitor, Baf A1, and of an autophagy inducer, rapamycin, on the infection rate of L. m.-infected BMDM in the late infection phase. BMDM were infected with luciferase-transgenic L. m. promastigotes. Baf A1 or rapamycin were added to BMDM 24 h p.i. when amastigote differentiation was completed and autophagy was fully induced. Then, BMDM were incubated for further 24 h. Untreated L. m.-infected BMDM were incubated for the same amount of time in RPMI. After cell lysis with a luciferin-containing buffer, the IC50-values for Baf A1 and rapamycin against L. m. amastigotes and BMDM were determined by the resulting luminescence. Results: (A) There were no IC50-values determinable. Thus, no decline of the infection rate by Baf A1 and rapamycin was detectable in BMDM. (B) Measurement of luminescence showed a dose-dependent increase of the infection rate with increasing Baf A1 concentrations applied to inhibit the lysosomal/autophagosomal acidification. Interestingly, no further enhancement of autophagic clearance with rapamycin was possible. Baf A1 = Bafilomycin A1, IC50 = half maximal inhibitory concentration, LU = luminescence unit, n.s. = not significant, (*) p ≤ 0.1, * p ≤ 0.05, + = maximally used concentration of compound. [file 13071_2015_974_MOESM11_ESM.pdf]

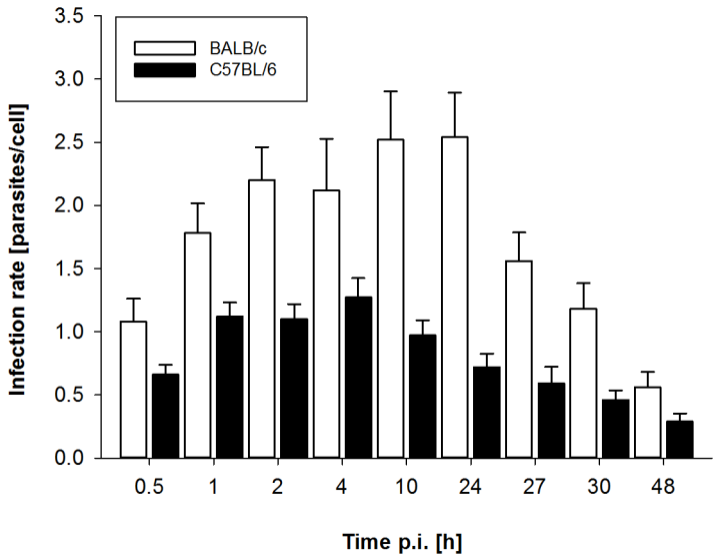

Supplement: Additional file 13: Figure S8. — Analysis of infection rates in L. m.-infected BMDM generated from BALB/c or C57BL/6 mice during time course experiments. Methods: BMDM either generated from BALB/c or C57BL/6 mice were infected with L. m. promastigotes. In a time frame from 0.5 to 48 h, L. m.-infected BMDM were harvested and the infection rates of L. m.-infected BMDM were determined by light microscopy after staining with Diff-Quik kit for each investigated time point. For each time point 50 individual BMDM were analyzed. Results: L. m.-infected BMDM generated from Leishmania-resistant C57BL/6 and Leishmania-susceptible BALB/c mice showed similar infection courses. In L. m.-infected BMDM generated from C57BL/6 mice, the infection rates were lower and declined faster compared to L. m.-infected BMDM generated from BALB/c mice. p.i. = post infection. [file 13071_2015_974_MOESM13_ESM.pdf]
