# Supplementary material for: Autophagic digestion of Leishmania major by host macrophages is associated with differential expression of BNIP3, CTSE, and the miRNAs miR-101c, miR-129, and miR-210
Source: Parasit Vectors. 2015 Jul 31;8:404. doi: 10.1186/s13071-015-0974-3 (PMC4521392; doi:10.1186/s13071-015-0974-3)
Supplement: Additional file 8: Table S1. — Differentially expressed genes between uninfected and L. m.-infected BMDM 1 h p.i. [file 13071_2015_974_MOESM8_ESM.docx]

**Table S1.** Differentially expressed genes between uninfected and *L. m.-*infected BMDM 1 h p.i..

| **Affymetrix ID** | **Gene name** | **Symbol** | **logFC** | **FDR** |
| --- | --- | --- | --- | --- |
| **1424976_at** | **ras homolog gene family, member V** | ***Rhov*** | 0.766 | 0.000 |
| 1425895_a_at | inhibitor of DNA binding 1 | *Id1* | -0.562 | 0.000 |
| 1444987_at | cathepsin B | *Ctsb* | 0.718 | 0.000 |
| 1443673_x_at | NA | *NA* | -0.534 | 0.000 |
| 1416111_at | CD83 antigen | *Cd83* | 0.538 | 0.000 |
| **1427736_a_at** | **chemokine (C-C motif) receptor-like 2** | ***Ccrl2*** | 0.567 | 0.000 |
| 1443208_at | NA | *NA* | 0.491 | 0.000 |
| **1449954_at** | **NA** | ***NA*** | 0.494 | 0.000 |
| **1418649_at** | **EGL nine homolog 3 (C. elegans)** | ***Egln3*** | 0.596 | 0.001 |
| 1440790_x_at | NA | *NA* | -0.452 | 0.001 |
| **1419561_at** | **chemokine (C-C motif) ligand 3** | ***Ccl3*** | 0.782 | 0.002 |
| 1415834_at | dual specificity phosphatase 6 | *Dusp6* | -0.423 | 0.002 |
| 1446861_at | glucosamine (N-acetyl)-6-sulfatase | *Gns* | 0.429 | 0.003 |
| 1449363_at | activating transcription factor 3 | *Atf3* | 0.505 | 0.003 |
| **1424915_s_at** | **RIKEN cDNA 2310044G17 gene** | ***2310044G17Rik*** | 0.416 | 0.003 |
| 1445626_at | NA | *NA* | 0.481 | 0.004 |
| **1429682_at** | **family with sequence similarity 46, member C** | ***Fam46c*** | 0.406 | 0.004 |
| 1453596_at | inhibitor of DNA binding 2 | *Id2* | 0.413 | 0.004 |
| **1445583_x_at** | **NA** | ***NA*** | 0.417 | 0.005 |
| **1417601_at** | **regulator of G-protein signaling 1** | ***Rgs1*** | 0.463 | 0.006 |
| 1425965_at | ubiquitin C | *Ubc* | 0.403 | 0.006 |
| **1418025_at** | **basic helix-loop-helix family, member e40** | ***Bhlhe40*** | 0.417 | 0.006 |
| 1443534_at | NA | *NA* | -0.397 | 0.006 |
| **1419209_at** | **chemokine (C-X-C motif) ligand 1** | ***Cxcl1*** | 0.668 | 0.007 |
| 1443504_at | uncharacterized protein E330022O07 | *E330022O07* | 0.396 | 0.007 |
| 1424155_at | fatty acid binding protein 4, adipocyte | *Fabp4* | 0.478 | 0.007 |
| **1422470_at** | **BCL2/adenovirus E1B interacting protein 3** | ***Bnip3*** | 0.417 | 0.007 |
| **1434130_at** | **lipoma HMGIC fusion partner-like 2** | ***Lhfpl2*** | 0.378 | 0.007 |
| 1423142_a_at | GTP binding protein 4 | *Gtpbp4* | -0.381 | 0.007 |
| **1452160_at** | **TCDD-inducible poly(ADP-ribose) polymerase** | ***Tiparp*** | 0.459 | 0.013 |
| **1454742_at** | **RasGEF domain family, member 1B** | ***Rasgef1b*** | 0.461 | 0.013 |
| **1433699_at** | **tumor necrosis factor, alpha-induced protein 3** | ***Tnfaip3*** | 0.487 | 0.015 |
| 1441788_s_at | dyskeratosis congenita 1, dyskerin homolog (human) | *Dkc1* | -0.379 | 0.018 |
| 1416250_at | B cell translocation gene 2, anti-proliferative | *Btg2* | -0.350 | 0.018 |
| 1430165_at | serine/threonine kinase 17b (apoptosis-inducing) | *Stk17b* | -0.376 | 0.019 |
| 1459171_at | epithelial membrane protein 1 | *Emp1* | 0.408 | 0.019 |
| 1437244_at | growth arrest-specific 2 like 3 | *Gas2l3* | 0.356 | 0.019 |
| 1438796_at | nuclear receptor subfamily 4, group A, member 3 | *Nr4a3* | 0.363 | 0.019 |
| **1423233_at** | **CCAAT/enhancer binding protein (C/EBP), delta** | ***Cebpd*** | -0.385 | 0.020 |
| **1439819_at** | **expressed sequence AU015263** | ***AU015263*** | -0.354 | 0.024 |
| **1450976_at** | **N-myc downstream regulated gene 1** | ***Ndrg1*** | 0.385 | 0.026 |
| 1455980_a_at | growth arrest-specific 2 like 3 | *Gas2l3* | 0.340 | 0.026 |
| 1417168_a_at | ubiquitin specific peptidase 2 | *Usp2* | 0.341 | 0.027 |
| 1458292_at | proteasome (prosome, macropain) subunit,  alpha type 1 | *Psma1* | -0.349 | 0.027 |
| 1426721_s_at | TCDD-inducible poly(ADP-ribose) polymerase | *Tiparp* | 0.384 | 0.028 |
| **1441887_x_at** | **predicted gene 6377** | ***Gm6377*** | 0.393 | 0.030 |
| 1441360_at | NA | *NA* | -0.347 | 0.030 |
| 1454847_at | lipoma HMGIC fusion partner-like 2 | *Lhfpl2* | 0.343 | 0.030 |
| 1441894_s_at | GRP1 (general receptor for phosphoinositides 1)-associated scaffold protein | *Grasp* | 0.332 | 0.035 |
| 1447408_at | NA | *NA* | -0.336 | 0.035 |
| 1460096_at | NA | *NA* | 0.360 | 0.035 |
| 1447527_at | NA | *NA* | -0.330 | 0.035 |
| **1457644_s_at** | **chemokine (C-X-C motif) ligand 1** | ***Cxcl1*** | 0.600 | 0.035 |
| **1434129_s_at** | **lipoma HMGIC fusion partner-like 2** | ***Lhfpl2*** | 0.327 | 0.035 |
| 1440563_at | NA | *NA* | 0.330 | 0.035 |
| 1457539_at | DNA segment, Chr 10, ERATO Doi 709, expressed | *D10Ertd709e* | 0.452 | 0.038 |
| 1436480_at | dipeptidylpeptidase 7 | *Dpp7* | 0.322 | 0.040 |
| 1456661_at | NA | *NA* | -0.320 | 0.040 |
| **1419697_at** | **chemokine (C-X-C motif) ligand 11** | ***Cxcl11*** | -0.386 | 0.044 |
| 1440721_at | RIKEN cDNA 5930433N17 gene | *5930433N17Rik* | -0.323 | 0.048 |
| **1419030_at** | **ERO1-like (S. cerevisiae)** | ***Ero1l*** | 0.342 | 0.049 |

Bold font = genes significantly regulated in 1 h and 24 h samples.
