# Supplementary material for: Autophagic digestion of Leishmania major by host macrophages is associated with differential expression of BNIP3, CTSE, and the miRNAs miR-101c, miR-129, and miR-210
Source: Parasit Vectors. 2015 Jul 31;8:404. doi: 10.1186/s13071-015-0974-3 (PMC4521392; doi:10.1186/s13071-015-0974-3)
Supplement: Additional file 9: Table S2. — Differentially expressed genes between uninfected and L. m.-infected BMDM 24 h p.i. [file 13071_2015_974_MOESM9_ESM.docx]

**Table S2.** Differentially expressed genes between uninfected and *L. m.-*infected BMDM 24 h p.i..

| **Affymetrix ID** | **Gene name** | **Symbol** | **logFC** | **FDR** |
| --- | --- | --- | --- | --- |
| 1427381_at | immunoresponsive gene 1 | *Irg1* | 4.232 | 0.000 |
| 1421009_at | radical S-adenosyl methionine domain containing 2 | *Rsad2* | 2.096 | 0.000 |
| 1421008_at | radical S-adenosyl methionine domain containing 2 | *Rsad2* | 1.997 | 0.000 |
| 1436058_at | radical S-adenosyl methionine domain containing 2 | *Rsad2* | 1.918 | 0.000 |
| 1450826_a_at | serum amyloid A 3 | *Saa3* | 1.761 | 0.000 |
| 1431591_s_at | NA | *NA* | 1.695 | 0.000 |
| 1450783_at | interferon-induced protein  with tetratricopeptide repeats 1 | *Ifit1* | 1.607 | 0.000 |
| 1418126_at | chemokine (C-C motif) ligand 5 | *Ccl5* | 2.250 | 0.000 |
| 1435906_x_at | guanylate binding protein 2 | *Gbp2* | 1.602 | 0.000 |
| 1450484_a_at | cytidine monophosphate (UMP-CMP) kinase 2, mitochondrial | *Cmpk2* | 1.576 | 0.000 |
| 1418930_at | chemokine (C-X-C motif) ligand 10 | *Cxcl10* | 2.023 | 0.000 |
| 1438676_at | guanylate binding protein 6 | *Gbp6* | 1.507 | 0.000 |
| 1449025_at | interferon-induced protein  with tetratricopeptide repeats 3 | *Ifit3* | 1.563 | 0.000 |
| 1418293_at | interferon-induced protein  with tetratricopeptide repeats 2 | *Ifit2* | 1.534 | 0.000 |
| 1418240_at | guanylate binding protein 2 | *Gbp2* | 1.553 | 0.000 |
| 1450387_s_at | adenylate kinase 4 | *Ak4* | 1.387 | 0.000 |
| 1449009_at | NA | *NA* | 1.397 | 0.000 |
| 1435665_at | tripartite motif-containing 30D | *Trim30d* | 1.327 | 0.000 |
| 1420591_at | G protein-coupled receptor 84 | *Gpr84* | 1.338 | 0.000 |
| 1420549_at | guanylate binding protein 1 | *Gbp1* | 1.349 | 0.000 |
| 1423954_at | complement component 3 | *C3* | 1.294 | 0.000 |
| 1439221_s_at | CD40 antigen | *Cd40* | 1.265 | 0.000 |
| 1419029_at | ERO1-like (S. cerevisiae) | *Ero1l* | 1.226 | 0.000 |
| **1418649_at** | **EGL nine homolog 3 (C. elegans)** | ***Egln3*** | 1.604 | 0.000 |
| **1419697_at** | **chemokine (C-X-C motif) ligand 11** | ***Cxcl11*** | 1.205 | 0.000 |
| 1438868_at | NA | *NA* | 1.160 | 0.000 |
| 1448383_at | matrix metallopeptidase 14 (membrane-inserted) | *Mmp14* | 1.156 | 0.000 |
| 1451905_a_at | myxovirus (influenza virus) resistance 1 | *Mx1* | 1.226 | 0.000 |
| 1427102_at | schlafen 4 | *Slfn4* | 1.146 | 0.000 |
| 1419714_at | CD274 antigen | *Cd274* | 1.134 | 0.000 |
| 1447927_at | NA | *NA* | 1.137 | 0.000 |
| 1460415_a_at | CD40 antigen | *Cd40* | 1.136 | 0.000 |
| 1460038_at | POU domain, class 3, transcription factor 1 | *Pou3f1* | 1.096 | 0.000 |
| 1425294_at | SLAM family member 8 | *Slamf8* | 1.171 | 0.000 |
| 1421830_at | adenylate kinase 4 | *Ak4* | 1.094 | 0.000 |
| 1419603_at | interferon activated gene 204 | *Ifi204* | 1.148 | 0.000 |
| 1418191_at | ubiquitin specific peptidase 18 | *Usp18* | 1.079 | 0.000 |
| 1429947_a_at | Z-DNA binding protein 1 | *Zbp1* | 1.045 | 0.000 |
| 1419569_a_at | interferon-stimulated protein | *Isg20* | 1.051 | 0.000 |
| 1434372_at | expressed sequence AW112010 | *AW112010* | 1.116 | 0.000 |
| 1419043_a_at | interferon inducible GTPase 1 | *Iigp1* | 1.078 | 0.000 |
| 1428834_at | dual specificity phosphatase 4 | *Dusp4* | 0.966 | 0.000 |
| 1449473_s_at | CD40 antigen | *Cd40* | 0.958 | 0.000 |
| 1447839_x_at | adrenomedullin | *Adm* | 1.068 | 0.000 |
| **1419030_at** | **ERO1-like (S. cerevisiae)** | ***Ero1l*** | 0.947 | 0.000 |
| 1417193_at | superoxide dismutase 2, mitochondrial | *Sod2* | 1.013 | 0.000 |
| 1449324_at | ERO1-like (S. cerevisiae) | *Ero1l* | 1.021 | 0.000 |
| 1438037_at | hect domain and RLD 6 | *Herc6* | 0.949 | 0.000 |
| 1436538_at | ankyrin repeat domain 37 | *Ankrd37* | 0.915 | 0.000 |
| 1435331_at | pyrin and HIN domain family, member 1 | *Pyhin1* | 0.936 | 0.000 |
| 1428735_at | CD69 antigen | *Cd69* | 0.980 | 0.000 |
| 1429678_at | RIKEN cDNA 5730508B09 gene | *5730508B09Rik* | 0.938 | 0.000 |
| 1418392_a_at | guanylate binding protein 3 | *Gbp3* | 0.919 | 0.000 |
| 1419042_at | interferon inducible GTPase 1 | *Iigp1* | 0.932 | 0.000 |
| 1435529_at | predicted gene 14446 | *Gm14446* | 0.909 | 0.000 |
| 1448610_a_at | superoxide dismutase 2, mitochondrial | *Sod2* | 0.960 | 0.000 |
| **1433699_at** | **tumor necrosis factor, alpha-induced protein 3** | ***Tnfaip3*** | 0.876 | 0.000 |
| 1417141_at | interferon gamma induced GTPase | *Igtp* | 0.894 | 0.000 |
| 1455096_at | fibronectin leucine rich transmembrane protein 2 | *Flrt2* | 0.875 | 0.000 |
| 1440342_at | RIKEN cDNA G530011O06 gene | *G530011O06Rik* | 0.992 | 0.000 |
| 1425663_at | interleukin 1 receptor antagonist | *Il1rn* | 0.872 | 0.000 |
| 1416077_at | adrenomedullin | *Adm* | 0.936 | 0.000 |
| 1448955_s_at | Ca2+-dependent secretion activator | *Cadps* | 1.079 | 0.000 |
| 1451385_at | family with sequence similarity 162, member A | *Fam162a* | 0.843 | 0.000 |
| 1437635_at | NA | *NA* | 0.911 | 0.000 |
| 1422953_at | formyl peptide receptor 2 | *Fpr2* | 1.403 | 0.000 |
| 1417292_at | interferon gamma inducible protein 47 | *Ifi47* | 0.964 | 0.000 |
| 1428660_s_at | torsin family 3, member A | *Tor3a* | 0.887 | 0.000 |
| 1447100_s_at | RIKEN cDNA 5730508B09 gene | *5730508B09Rik* | 0.813 | 0.000 |
| 1421366_at | C-type lectin domain family 5, member a | *Clec5a* | 0.804 | 0.000 |
| 1453939_x_at | predicted gene 9706 | *Gm9706* | 0.801 | 0.000 |
| 1418825_at | immunity-related GTPase family M member 1 | *Irgm1* | 0.793 | 0.000 |
| 1427345_a_at | sulfotransferase family 1A, phenol-preferring,  member 1 | *Sult1a1* | -1.104 | 0.000 |
| 1419607_at | tumor necrosis factor | *Tnf* | 0.784 | 0.000 |
| 1423555_a_at | interferon-induced protein 44 | *Ifi44* | 0.847 | 0.000 |
| 1450034_at | signal transducer and activator of transcription 1 | *Stat1* | 0.784 | 0.000 |
| 1418989_at | cathepsin E | *Ctse* | -0.852 | 0.000 |
| **1419209_at** | **chemokine (C-X-C motif) ligand 1** | ***Cxcl1*** | 0.808 | 0.000 |
| 1422160_at | histocompatibility 2, T region locus 24 | *H2-T24* | 0.847 | 0.000 |
| 1419604_at | Z-DNA binding protein 1 | *Zbp1* | 0.915 | 0.000 |
| 1453196_a_at | 2'-5' oligoadenylate synthetase-like 2 | *Oasl2* | 0.762 | 0.000 |
| 1450430_at | mannose receptor, C type 1 | *Mrc1* | -1.053 | 0.000 |
| 1417149_at | procollagen-proline, 2-oxoglutarate 4-dioxygenase (proline 4-hydroxylase), alpha II polypeptide | *P4ha2* | 0.804 | 0.000 |
| 1422438_at | epoxide hydrolase 1, microsomal | *Ephx1* | -0.892 | 0.000 |
| 1438702_at | fibronectin leucine rich transmembrane protein 2 | *Flrt2* | 0.828 | 0.000 |
| 1449270_at | plexin domain containing 2 | *Plxdc2* | -0.739 | 0.000 |
| 1429175_at | transmembrane protein 178 | *Tmem178* | 0.824 | 0.000 |
| 1423883_at | acyl-CoA synthetase long-chain family member 1 | *Acsl1* | 0.822 | 0.000 |
| 1429184_at | GTPase, very large interferon inducible 1 | *Gvin1* | 0.803 | 0.000 |
| 1424923_at | serine (or cysteine) peptidase inhibitor,  clade A, member 3G | *Serpina3g* | 0.778 | 0.000 |
| 1438511_a_at | RIKEN cDNA 1190002H23 gene | *1190002H23Rik* | 0.749 | 0.000 |
| 1440920_at | matrix metallopeptidase 14 (membrane-inserted) | *Mmp14* | 0.735 | 0.001 |
| 1443698_at | XIAP associated factor 1 | *Xaf1* | 0.717 | 0.001 |
| 1421998_at | torsin family 3, member A | *Tor3a* | 0.718 | 0.001 |
| 1422138_at | plasminogen activator, urokinase | *Plau* | 0.733 | 0.001 |
| 1450643_s_at | acyl-CoA synthetase long-chain family member 1 | *Acsl1* | 0.712 | 0.001 |
| 1420654_a_at | glucan (1,4-alpha-), branching enzyme 1 | *Gbe1* | 0.750 | 0.001 |
| 1455500_at | ring finger protein 213 | *Rnf213* | 0.760 | 0.001 |
| 1420774_a_at | RIKEN cDNA 4930583H14 gene | *4930583H14Rik* | 0.702 | 0.001 |
| 1436172_at | predicted gene, 20559 | *Gm20559* | 0.700 | 0.001 |
| 1443414_at | EST C78513 | *C78513* | 0.731 | 0.001 |
| **1457644_s_at** | **chemokine (C-X-C motif) ligand 1** | ***Cxcl1*** | 0.697 | 0.001 |
| 1417793_at | immunity-related GTPase family M member 2 | *Irgm2* | 0.718 | 0.001 |
| 1451563_at | EGF-like module containing, mucin-like,  hormone receptor-like sequence 4 | *Emr4* | -1.299 | 0.001 |
| 1429514_at | phosphatidic acid phosphatase type 2B | *Ppap2b* | 0.695 | 0.001 |
| 1418652_at | chemokine (C-X-C motif) ligand 9 | *Cxcl9* | 0.698 | 0.001 |
| 1416010_a_at | EH-domain containing 1 | *Ehd1* | 0.742 | 0.001 |
| 1425917_at | histocompatibility 28 | *H28* | 0.770 | 0.001 |
| 1416335_at | macrophage migration inhibitory factor | *Mif* | 0.731 | 0.001 |
| 1450652_at | cathepsin K | *Ctsk* | 0.684 | 0.001 |
| 1424998_at | EGF-like module containing, mucin-like,  hormone receptor-like sequence 4 | *Emr4* | -1.399 | 0.001 |
| 1418829_a_at | enolase 2, gamma neuronal | *Eno2* | 0.673 | 0.001 |
| **1422470_at** | **BCL2/adenovirus E1B interacting protein 3** | ***Bnip3*** | 0.765 | 0.001 |
| 1421578_at | chemokine (C-C motif) ligand 4 | *Ccl4* | 0.710 | 0.001 |
| 1435792_at | NA | *NA* | 0.683 | 0.001 |
| 1450454_at | torsin family 3, member A | *Tor3a* | 0.701 | 0.001 |
| 1428420_a_at | RIKEN cDNA 1200009I06 gene | *1200009I06Rik* | 0.667 | 0.001 |
| 1417023_a_at | fatty acid binding protein 4, adipocyte | *Fabp4* | 0.694 | 0.001 |
| 1416749_at | HtrA serine peptidase 1 | *Htra1* | 0.698 | 0.001 |
| 1415918_a_at | triosephosphate isomerase 1 | *Tpi1* | 0.670 | 0.001 |
| 1426276_at | interferon induced with helicase C domain 1 | *Ifih1* | 0.664 | 0.001 |
| 1419004_s_at | NA | *NA* | 0.786 | 0.001 |
| 1453757_at | hect domain and RLD 6 | *Herc6* | 0.772 | 0.001 |
| 1455870_at | A kinase (PRKA) anchor protein 2 | *Akap2* | 0.667 | 0.001 |
| 1417864_at | phosphoglycerate kinase 1 | *Pgk1* | 0.730 | 0.001 |
| **1424976_at** | **ras homolog gene family, member V** | ***Rhov*** | 0.798 | 0.001 |
| 1449305_at | coagulation factor X | *F10* | 0.717 | 0.001 |
| 1422141_s_at | component of Sp100-rs | *Csprs* | 0.726 | 0.001 |
| 1439831_at | NA | *NA* | 0.715 | 0.001 |
| 1427691_a_at | interferon (alpha and beta) receptor 2 | *Ifnar2* | -0.688 | 0.001 |
| 1416011_x_at | EH-domain containing 1 | *Ehd1* | 0.645 | 0.001 |
| 1424882_a_at | 5'-nucleotidase domain containing 2 | *Nt5dc2* | -0.697 | 0.001 |
| **1449954_at** | **NA** | ***NA*** | 0.754 | 0.001 |
| 1447851_x_at | ATPase, class V, type 10A | *Atp10a* | 0.639 | 0.001 |
| **1419561_at** | **chemokine (C-C motif) ligand 3** | ***Ccl3*** | 0.643 | 0.001 |
| 1439764_s_at | insulin-like growth factor 2 mRNA binding protein 2 | *Igf2bp2* | 0.632 | 0.001 |
| 1421596_s_at | histocompatibility 28 | *H28* | 0.777 | 0.001 |
| 1448990_a_at | myosin IB | *Myo1b* | 0.699 | 0.001 |
| 1448698_at | cyclin D1 | *Ccnd1* | 0.656 | 0.001 |
| 1431095_a_at | hect domain and RLD 6 | *Herc6* | 0.673 | 0.001 |
| 1420499_at | GTP cyclohydrolase 1 | *Gch1* | 0.643 | 0.001 |
| 1453472_a_at | SLAM family member 7 | *Slamf7* | 0.634 | 0.001 |
| 1422924_at | tumor necrosis factor (ligand) superfamily,  member 9 | *Tnfsf9* | 0.726 | 0.001 |
| 1418648_at | EGL nine homolog 3 (C. elegans) | *Egln3* | 0.624 | 0.001 |
| 1452927_x_at | triosephosphate isomerase 1 | *Tpi1* | 0.634 | 0.001 |
| 1420380_at | chemokine (C-C motif) ligand 2 | *Ccl2* | 0.738 | 0.002 |
| 1451050_at | 5'-nucleotidase, cytosolic III | *Nt5c3* | 0.624 | 0.002 |
| 1423757_x_at | insulin-like growth factor binding protein 4 | *Igfbp4* | -0.792 | 0.002 |
| 1459679_s_at | myosin IB | *Myo1b* | 0.676 | 0.002 |
| 1454731_at | myosin X | *Myo10* | 0.620 | 0.002 |
| 1451263_a_at | fatty acid binding protein 4, adipocyte | *Fabp4* | 0.617 | 0.002 |
| 1428838_a_at | deoxycytidine kinase | *Dck* | 0.648 | 0.002 |
| 1417523_at | pleckstrin | *Plek* | 0.629 | 0.002 |
| 1438640_x_at | phosphoglycerate kinase 1 | *Pgk1* | 0.614 | 0.002 |
| **1454742_at** | **RasGEF domain family, member 1B** | ***Rasgef1b*** | 0.614 | 0.002 |
| 1435659_a_at | triosephosphate isomerase 1 | *Tpi1* | 0.625 | 0.002 |
| 1451461_a_at | aldolase C, fructose-bisphosphate | *Aldoc* | 0.636 | 0.002 |
| 1423436_at | glutathione S-transferase, alpha 3 | *Gsta3* | 0.701 | 0.002 |
| 1427242_at | DEAD (Asp-Glu-Ala-Asp) box polypeptide 4 | *Ddx4* | 0.616 | 0.002 |
| **1427736_a_at** | **chemokine (C-C motif) receptor-like 2** | ***Ccrl2*** | 0.644 | 0.002 |
| 1440879_at | ATP-binding cassette, sub-family A (ABC1),  member 9 | *Abca9* | -1.011 | 0.002 |
| 1423756_s_at | insulin-like growth factor binding protein 4 | *Igfbp4* | -0.916 | 0.002 |
| 1438716_at | tripartite motif-containing 30D | *Trim30d* | 0.633 | 0.002 |
| 1423804_a_at | isopentenyl-diphosphate delta isomerase | *Idi1* | 0.696 | 0.002 |
| 1438251_x_at | HtrA serine peptidase 1 | *Htra1* | 0.595 | 0.002 |
| 1450906_at | plexin C1 | *Plxnc1* | -0.610 | 0.002 |
| 1419721_at | niacin receptor 1 | *Niacr1* | 0.612 | 0.002 |
| 1422542_at | G protein-coupled receptor 34 | *Gpr34* | -1.120 | 0.002 |
| 1417420_at | cyclin D1 | *Ccnd1* | 0.617 | 0.002 |
| 1449124_at | ral guanine nucleotide dissociation stimulator,-like 1 | *Rgl1* | 0.639 | 0.002 |
| 1422139_at | plasminogen activator, urokinase | *Plau* | 0.589 | 0.002 |
| 1416067_at | interferon-related developmental regulator 1 | *Ifrd1* | 0.601 | 0.002 |
| 1436781_at | mannosidase 2, alpha B1 | *Man2b1* | -0.722 | 0.003 |
| 1421340_at | mitogen-activated protein kinase kinase kinase 5 | *Map3k5* | -0.622 | 0.003 |
| 1439181_at | zinc finger protein 658 | *Zfp658* | -0.670 | 0.003 |
| 1448134_at | cDNA sequence X99384 | *X99384* | -0.643 | 0.003 |
| 1444003_at | neuralized homolog 3 homolog (Drosophila) | *Neurl3* | -0.744 | 0.003 |
| 1429146_at | small VCP/p97-interacting protein | *Svip* | -0.664 | 0.003 |
| 1455318_at | T cell immunoglobulin and mucin domain  containing 4 | *Timd4* | 0.606 | 0.003 |
| 1455581_x_at | predicted gene, 20559 | *Gm20559* | 0.608 | 0.003 |
| 1422095_a_at | cytidine monophosphate (UMP-CMP) kinase 2, mitochondrial | *Cmpk2* | 1.002 | 0.003 |
| 1435975_at | DENN/MADD domain containing 4A | *Dennd4a* | -0.659 | 0.003 |
| 1453234_at | RIKEN cDNA 1300002K09 gene | *1300002K09Rik* | 0.619 | 0.003 |
| 1456080_a_at | serine incorporator 3 | *Serinc3* | -0.594 | 0.003 |
| 1417481_at | receptor (calcitonin) activity modifying protein 1 | *Ramp1* | -0.708 | 0.003 |
| 1418912_at | plexin domain containing 2 | *Plxdc2* | -0.688 | 0.003 |
| 1451777_at | DEAD (Asp-Glu-Ala-Asp) box polypeptide 60 | *Ddx60* | 0.598 | 0.003 |
| 1420671_x_at | membrane-spanning 4-domains, subfamily A,  member 4C | *Ms4a4c* | 0.564 | 0.003 |
| 1437405_a_at | insulin-like growth factor binding protein 4 | *Igfbp4* | -0.881 | 0.003 |
| 1434583_at | transmembrane protein 26 | *Tmem26* | 0.561 | 0.003 |
| 1448509_at | family with sequence similarity 107, member B | *Fam107b* | -0.703 | 0.003 |
| 1419737_a_at | lactate dehydrogenase A | *Ldha* | 0.558 | 0.003 |
| 1447624_s_at | storkhead box 2 | *Stox2* | 0.658 | 0.003 |
| 1437176_at | NLR family, CARD domain containing 5 | *Nlrc5* | 0.559 | 0.003 |
| 1448908_at | phosphatidic acid phosphatase type 2B | *Ppap2b* | 0.567 | 0.003 |
| 1422824_s_at | epidermal growth factor receptor pathway  substrate 8 | *Eps8* | -0.734 | 0.004 |
| 1456874_at | fibronectin leucine rich transmembrane protein 2 | *Flrt2* | 0.587 | 0.004 |
| 1456288_at | schlafen 5 | *Slfn5* | 0.602 | 0.004 |
| 1423161_s_at | sprouty protein with EVH-1 domain 1,  related sequence | *Spred1* | 0.551 | 0.004 |
| 1435836_at | pyruvate dehydrogenase kinase, isoenzyme 1 | *Pdk1* | 0.668 | 0.004 |
| 1440315_at | muscleblind-like 1 (Drosophila) | *Mbnl1* | -0.553 | 0.004 |
| 1451122_at | isopentenyl-diphosphate delta isomerase | *Idi1* | 0.684 | 0.004 |
| 1436164_at | solute carrier family 30 (zinc transporter), member 1 | *Slc30a1* | 0.555 | 0.004 |
| 1456126_at | mucosa associated lymphoid tissue lymphoma translocation gene 1 | *Malt1* | 0.578 | 0.004 |
| 1428781_at | dermokine | *Dmkn* | -0.549 | 0.004 |
| 1416630_at | inhibitor of DNA binding 3 | *Id3* | -0.808 | 0.004 |
| 1436515_at | BTB and CNC homology 2 | *Bach2* | -0.698 | 0.004 |
| 1420760_s_at | N-myc downstream regulated gene 1 | *Ndrg1* | 0.545 | 0.004 |
| 1434580_at | ectonucleotide pyrophosphatase/  phosphodiesterase 4 | *Enpp4* | 0.581 | 0.004 |
| 1452646_at | transformation related protein 53 inducible nuclear protein 2 | *Trp53inp2* | -0.608 | 0.004 |
| 1448929_at | coagulation factor XIII, A1 subunit | *F13a1* | 0.783 | 0.004 |
| 1428242_at | histocompatibility (minor) HA-1 | *Hmha1* | -0.601 | 0.004 |
| 1448793_a_at | syndecan 4 | *Sdc4* | 0.541 | 0.004 |
| 1439012_a_at | deoxycytidine kinase | *Dck* | 0.554 | 0.004 |
| 1451564_at | poly (ADP-ribose) polymerase family, member 14 | *Parp14* | 0.557 | 0.004 |
| 1439435_x_at | phosphoglycerate kinase 1 | *Pgk1* | 0.544 | 0.004 |
| 1448251_at | RIKEN cDNA 9030425E11 gene | *9030425E11Rik* | -0.893 | 0.004 |
| 1434815_a_at | mitogen-activated protein kinase-activated protein kinase 3 | *Mapkapk3* | -0.591 | 0.005 |
| 1456442_at | RAB3A interacting protein (rabin3)-like 1 | *Rab3il1* | -0.558 | 0.005 |
| 1453072_at | G protein-coupled receptor 160 | *Gpr160* | -0.627 | 0.005 |
| 1425374_at | 2'-5' oligoadenylate synthetase 3 | *Oas3* | 0.545 | 0.005 |
| 1418116_at | torsin A interacting protein 2 | *Tor1aip2* | 0.533 | 0.005 |
| 1424176_a_at | annexin A4 | *Anxa4* | 0.550 | 0.005 |
| **1445583_x_at** | **NA** | ***NA*** | 0.584 | 0.005 |
| 1421402_at | metastasis associated 3 | *Mta3* | -0.607 | 0.005 |
| 1440866_at | eukaryotic translation initiation factor 2-alpha  kinase 2 | *Eif2ak2* | 0.533 | 0.005 |
| 1422533_at | cytochrome P450, family 51 | *Cyp51* | 0.530 | 0.005 |
| 1437514_at | RIKEN cDNA B430306N03 gene | *B430306N03Rik* | 0.570 | 0.005 |
| 1426645_at | heat shock protein 90, alpha (cytosolic),  class A member 1 | *Hsp90aa1* | 0.543 | 0.005 |
| 1455992_at | vestigial like 4 (Drosophila) | *Vgll4* | -0.563 | 0.005 |
| 1425214_at | pyrimidinergic receptor P2Y, G-protein coupled, 6 | *P2ry6* | -0.544 | 0.005 |
| 1426554_a_at | phosphoglycerate mutase 1 | *Pgam1* | 0.546 | 0.005 |
| 1437584_at | NA | *NA* | -0.609 | 0.005 |
| 1421228_at | chemokine (C-C motif) ligand 7 | *Ccl7* | 0.523 | 0.005 |
| 1418937_at | deiodinase, iodothyronine, type II | *Dio2* | 0.905 | 0.005 |
| 1449317_at | CASP8 and FADD-like apoptosis regulator | *Cflar* | 0.545 | 0.005 |
| 1441105_at | predicted gene 11110 | *Gm11110* | 0.536 | 0.005 |
| **1450976_at** | **N-myc downstream regulated gene 1** | ***Ndrg1*** | 0.539 | 0.005 |
| 1417314_at | NA | *NA* | 0.573 | 0.005 |
| 1450291_s_at | membrane-spanning 4-domains, subfamily A,  member 4C | *Ms4a4c* | 0.525 | 0.005 |
| 1437406_x_at | insulin-like growth factor binding protein 4 | *Igfbp4* | -0.730 | 0.005 |
| 1431705_a_at | mucolipin 2 | *Mcoln2* | 0.518 | 0.006 |
| 1438069_a_at | RNA binding motif protein 5 | *Rbm5* | -0.517 | 0.006 |
| 1417116_at | solute carrier family 6  (neurotransmitter transporter, creatine), member 8 | *Slc6a8* | 0.517 | 0.006 |
| 1423160_at | sprouty protein with EVH-1 domain 1,  related sequence | *Spred1* | 0.521 | 0.006 |
| 1421027_a_at | myocyte enhancer factor 2C | *Mef2c* | -0.522 | 0.006 |
| 1424714_at | aldolase C, fructose-bisphosphate | *Aldoc* | 0.529 | 0.006 |
| 1440739_at | vascular endothelial growth factor C | *Vegfc* | 0.572 | 0.006 |
| 1424775_at | NA | *NA* | 0.540 | 0.006 |
| 1416892_s_at | family with sequence similarity 107, member B | *Fam107b* | -0.686 | 0.006 |
| 1427091_at | zinc finger, NFX1-type containing 1 | *Znfx1* | 0.515 | 0.006 |
| 1417256_at | matrix metallopeptidase 13 | *Mmp13* | 0.603 | 0.006 |
| 1433571_at | serine incorporator 5 | *Serinc5* | -0.662 | 0.006 |
| 1421821_at | low density lipoprotein receptor | *Ldlr* | 0.814 | 0.006 |
| 1460239_at | tetraspanin 13 | *Tspan13* | -0.516 | 0.006 |
| 1451680_at | sulfiredoxin 1 homolog (S. cerevisiae) | *Srxn1* | 0.513 | 0.006 |
| 1427404_x_at | predicted gene 5506 | *Gm5506* | 0.604 | 0.006 |
| 1424443_at | transmembrane 6 superfamily member 1 | *Tm6sf1* | -0.605 | 0.006 |
| 1422544_at | myosin X | *Myo10* | 0.517 | 0.006 |
| 1428392_at | Ras association (RalGDS/AF-6) domain family member 2 | *Rassf2* | -0.681 | 0.006 |
| 1448694_at | Jun oncogene | *Jun* | 0.505 | 0.007 |
| 1426112_a_at | CD72 antigen | *Cd72* | 0.505 | 0.007 |
| 1456494_a_at | NA | *NA* | 0.511 | 0.007 |
| 1419123_a_at | platelet-derived growth factor, C polypeptide | *Pdgfc* | -0.929 | 0.007 |
| 1417604_at | calcium/calmodulin-dependent protein kinase I | *Camk1* | -0.503 | 0.007 |
| 1423747_a_at | pyruvate dehydrogenase kinase, isoenzyme 1 | *Pdk1* | 0.651 | 0.007 |
| 1419601_at | potassium inwardly-rectifying channel, subfamily J, member 10 | *Kcnj10* | -0.777 | 0.007 |
| 1457248_x_at | hydroxysteroid (17-beta) dehydrogenase 7 | *Hsd17b7* | 0.504 | 0.007 |
| 1425125_at | oncoprotein induced transcript 3 | *Oit3* | 0.513 | 0.007 |
| 1423153_x_at | complement component factor h | *Cfh* | -0.763 | 0.007 |
| 1442116_at | G protein-coupled receptor 176 | *Gpr176* | 0.538 | 0.007 |
| 1448306_at | nuclear factor of kappa light polypeptide gene enhancer in B cells inhibitor, alpha | *Nfkbia* | 0.500 | 0.007 |
| 1419023_x_at | NA | *NA* | 0.621 | 0.007 |
| 1439680_at | tumor necrosis factor (ligand) superfamily,  member 10 | *Tnfsf10* | 0.495 | 0.008 |
| 1422526_at | acyl-CoA synthetase long-chain family member 1 | *Acsl1* | 0.495 | 0.008 |
| 1460003_at | expressed sequence AI956758 | *AI956758* | -0.689 | 0.008 |
| 1459913_at | tumor necrosis factor (ligand) superfamily,  member 10 | *Tnfsf10* | 0.495 | 0.008 |
| 1419676_at | myxovirus (influenza virus) resistance 2 | *Mx2* | 0.494 | 0.008 |
| 1419549_at | arginase, liver | *Arg1* | 0.531 | 0.008 |
| 1424067_at | intercellular adhesion molecule 1 | *Icam1* | 0.490 | 0.008 |
| 1450646_at | cytochrome P450, family 51 | *Cyp51* | 0.559 | 0.008 |
| 1425609_at | neutrophil cytosolic factor 1 | *Ncf1* | -0.491 | 0.008 |
| 1438730_at | neuron navigator 1 | *Nav1* | -0.489 | 0.008 |
| 1418643_at | tetraspanin 13 | *Tspan13* | -0.500 | 0.008 |
| 1456890_at | DEAD (Asp-Glu-Ala-Asp) box polypeptide 58 | *Ddx58* | 0.492 | 0.008 |
| 1429692_s_at | GTP cyclohydrolase 1 | *Gch1* | 0.538 | 0.008 |
| 1417189_at | proteasome (prosome, macropain) 28 subunit, beta | *Psme2* | 0.494 | 0.008 |
| 1421733_a_at | protein-tyrosine sulfotransferase 1 | *Tpst1* | 0.513 | 0.008 |
| 1437103_at | insulin-like growth factor 2 mRNA binding protein 2 | *Igf2bp2* | 0.504 | 0.008 |
| 1417541_at | helicase, lymphoid specific | *Hells* | 0.505 | 0.009 |
| 1419022_a_at | NA | *NA* | 0.625 | 0.009 |
| 1435349_at | neuropilin 2 | *Nrp2* | 0.485 | 0.009 |
| 1422823_at | epidermal growth factor receptor pathway  substrate 8 | *Eps8* | -0.652 | 0.009 |
| 1438157_s_at | nuclear factor of kappa light polypeptide gene enhancer in B cells inhibitor, alpha | *Nfkbia* | 0.483 | 0.009 |
| 1429639_at | glycerophosphocholine phosphodiesterase GDE1 homolog (S. cerevisiae) | *Gpcpd1* | -0.590 | 0.009 |
| 1457658_x_at | annexin A4 | *Anxa4* | 0.482 | 0.009 |
| 1451626_x_at | NA | *NA* | -0.632 | 0.009 |
| 1420905_at | interleukin 17 receptor A | *Il17ra* | -0.673 | 0.009 |
| 1452592_at | microsomal glutathione S-transferase 2 | *Mgst2* | -0.513 | 0.009 |
| 1417419_at | cyclin D1 | *Ccnd1* | 0.539 | 0.009 |
| 1436528_at | Kazal-type serine peptidase inhibitor domain 1 | *Kazald1* | -0.506 | 0.009 |
| 1453299_a_at | NA | *NA* | 0.483 | 0.009 |
| 1422535_at | cyclin E2 | *Ccne2* | 0.484 | 0.009 |
| 1424464_s_at | major facilitator superfamily domain containing 6 | *Mfsd6* | -0.507 | 0.009 |
| 1432543_a_at | Kruppel-like factor 13 | *Klf13* | -0.570 | 0.009 |
| 1438519_at | kelch-like 24 (Drosophila) | *Klhl24* | -0.489 | 0.009 |
| 1448239_at | heme oxygenase (decycling) 1 | *Hmox1* | 0.504 | 0.009 |
| 1421052_a_at | spermine synthase | *Sms* | 0.482 | 0.009 |
| 1422966_a_at | transferrin receptor | *Tfrc* | 0.494 | 0.009 |
| 1424594_at | sterile alpha motif domain containing 4 | *Samd4* | -0.796 | 0.009 |
| 1430309_at | Nipped-B homolog (Drosophila) | *Nipbl* | -0.527 | 0.009 |
| 1419609_at | chemokine (C-C motif) receptor 1 | *Ccr1* | 0.481 | 0.009 |
| 1449731_s_at | nuclear factor of kappa light polypeptide gene enhancer in B cells inhibitor, alpha | *Nfkbia* | 0.500 | 0.009 |
| 1436562_at | DEAD (Asp-Glu-Ala-Asp) box polypeptide 58 | *Ddx58* | 0.520 | 0.009 |
| 1459894_at | IQ motif containing GTPase activating protein 2 | *Iqgap2* | -0.600 | 0.009 |
| 1418046_at | nucleosome assembly protein 1-like 2 | *Nap1l2* | 0.479 | 0.009 |
| 1451767_at | neutrophil cytosolic factor 1 | *Ncf1* | -0.486 | 0.009 |
| 1457999_at | family with sequence similarity 82, member B | *Fam82b* | -0.724 | 0.009 |
| 1452353_at | G protein-coupled receptor 155 | *Gpr155* | -0.759 | 0.009 |
| 1456174_x_at | N-myc downstream regulated gene 1 | *Ndrg1* | 0.512 | 0.010 |
| 1433678_at | phospholipase D family, member 4 | *Pld4* | -0.485 | 0.010 |
| 1436194_at | PRELI domain containing 2 | *Prelid2* | 0.519 | 0.010 |
| 1435763_at | TBC1 domain family, member 16 | *Tbc1d16* | -0.472 | 0.010 |
| 1450061_at | ectodermal-neural cortex 1 | *Enc1* | -0.830 | 0.010 |
| 1417961_a_at | tripartite motif-containing 30A | *Trim30a* | 0.478 | 0.010 |
| 1451361_a_at | patatin-like phospholipase domain containing 7 | *Pnpla7* | -0.524 | 0.010 |
| 1416713_at | tubulin polymerization-promoting protein family member 3 | *Tppp3* | -0.473 | 0.010 |
| 1442804_at | Gardner-Rasheed feline sarcoma viral (Fgr) oncogene homolog | *Fgr* | 0.472 | 0.010 |
| 1448596_at | solute carrier family 6 (neurotransmitter transporter, creatine), member 8 | *Slc6a8* | 0.548 | 0.010 |
| 1452408_at | NA | *NA* | 0.474 | 0.010 |
| 1428029_a_at | H2A histone family, member V | *H2afv* | -0.479 | 0.010 |
| 1441907_s_at | CD93 antigen | *Cd93* | -0.471 | 0.010 |
| 1460634_at | ral guanine nucleotide dissociation stimulator | *Ralgds* | 0.468 | 0.010 |
| 1422567_at | family with sequence similarity 129, member A | *Fam129a* | -0.565 | 0.010 |
| 1456386_at | NA | *NA* | -0.570 | 0.010 |
| 1449453_at | bone marrow stromal cell antigen 1 | *Bst1* | 0.474 | 0.010 |
| 1437886_at | kelch-like 6 (Drosophila) | *Klhl6* | -0.637 | 0.010 |
| 1419417_at | vascular endothelial growth factor C | *Vegfc* | 0.470 | 0.010 |
| 1415997_at | thioredoxin interacting protein | *Txnip* | -0.466 | 0.011 |
| 1420946_at | alpha thalassemia/mental retardation syndrome X-linked homolog (human) | *Atrx* | -0.480 | 0.011 |
| 1422668_at | serine (or cysteine) peptidase inhibitor, clade B, member 9b | *Serpinb9b* | 0.521 | 0.011 |
| 1418770_at | CD2 antigen | *Cd2* | -0.619 | 0.011 |
| 1418809_at | NA | *NA* | 0.492 | 0.011 |
| 1457780_at | syntaxin 11 | *Stx11* | 0.465 | 0.011 |
| 1425107_a_at | leukemia inhibitory factor receptor | *Lifr* | -0.567 | 0.011 |
| 1452078_a_at | solute carrier family 11 (proton-coupled divalent metal ion transporters), member 2 | *Slc11a2* | 0.472 | 0.011 |
| 1416345_at | translocase of inner mitochondrial membrane 8 homolog a1 (yeast) | *Timm8a1* | 0.463 | 0.011 |
| 1451149_at | phosphoglucomutase 2 | *Pgm2* | 0.483 | 0.011 |
| 1416593_at | glutaredoxin | *Glrx* | 0.589 | 0.011 |
| 1451353_at | transmembrane 6 superfamily member 1 | *Tm6sf1* | -0.565 | 0.011 |
| 1423748_at | pyruvate dehydrogenase kinase, isoenzyme 1 | *Pdk1* | 0.476 | 0.011 |
| 1429723_at | RIKEN cDNA 6330409N04 gene | *6330409N04Rik* | 0.460 | 0.011 |
| 1423064_at | DNA methyltransferase 3A | *Dnmt3a* | 0.601 | 0.012 |
| 1442015_at | RIKEN cDNA 2500002B13 gene | *2500002B13Rik* | 0.460 | 0.012 |
| 1448250_at | RIKEN cDNA 9030425E11 gene | *9030425E11Rik* | -0.900 | 0.012 |
| 1417203_at | ethylmalonic encephalopathy 1 | *Ethe1* | -0.539 | 0.012 |
| 1417409_at | Jun oncogene | *Jun* | 0.465 | 0.012 |
| 1423437_at | glutathione S-transferase, alpha 3 | *Gsta3* | 0.457 | 0.012 |
| 1451132_at | pre B cell leukemia transcription factor interacting protein 1 | *Pbxip1* | -0.458 | 0.012 |
| 1457825_x_at | transcobalamin 2 | *Tcn2* | -0.599 | 0.012 |
| 1436223_at | integrin beta 8 | *Itgb8* | 0.458 | 0.012 |
| 1449156_at | lymphocyte antigen 9 | *Ly9* | 0.456 | 0.012 |
| 1416530_a_at | purine-nucleoside phosphorylase | *Pnp* | 0.461 | 0.012 |
| **1417601_at** | **regulator of G-protein signaling 1** | ***Rgs1*** | 0.456 | 0.012 |
| 1426936_at | NA | *NA* | -0.473 | 0.012 |
| 1427186_a_at | myocyte enhancer factor 2A | *Mef2a* | -0.489 | 0.012 |
| 1428853_at | patched homolog 1 | *Ptch1* | -0.549 | 0.012 |
| 1428479_at | nuclear factor of activated T cells, cytoplasmic, calcineurin dependent 1 | *Nfatc1* | -0.515 | 0.012 |
| 1455137_at | Rap guanine nucleotide exchange factor (GEF) 5 | *Rapgef5* | 0.453 | 0.013 |
| 1434150_a_at | NA | *NA* | -0.474 | 0.013 |
| 1429144_at | glycerophosphocholine phosphodiesterase GDE1 homolog (S. cerevisiae) | *Gpcpd1* | -0.498 | 0.013 |
| 1431997_at | glyceraldehyde-3-phosphate dehydrogenase pseudogene | *3000002C10Rik* | 0.486 | 0.013 |
| 1438684_at | NUAK family, SNF1-like kinase, 1 | *Nuak1* | -0.693 | 0.013 |
| 1454699_at | sestrin 1 | *Sesn1* | -0.595 | 0.013 |
| 1418580_at | receptor transporter protein 4 | *Rtp4* | 0.509 | 0.013 |
| **1423233_at** | **CCAAT/enhancer binding protein (C/EBP), delta** | ***Cebpd*** | -0.696 | 0.013 |
| 1436907_at | neuron navigator 1 | *Nav1* | -0.450 | 0.013 |
| 1420904_at | interleukin 17 receptor A | *Il17ra* | -0.478 | 0.013 |
| 1425281_a_at | TSC22 domain family, member 3 | *Tsc22d3* | -0.558 | 0.013 |
| 1421011_at | hydroxysteroid (17-beta) dehydrogenase 11 | *Hsd17b11* | -0.463 | 0.013 |
| 1416296_at | interleukin 2 receptor, gamma chain | *Il2rg* | 0.493 | 0.013 |
| 1416246_a_at | coronin, actin binding protein 1A | *Coro1a* | -0.449 | 0.013 |
| 1450084_s_at | influenza virus NS1A binding protein | *Ivns1abp* | -0.695 | 0.013 |
| 1448788_at | CD200 antigen | *Cd200* | 0.522 | 0.014 |
| 1419605_at | C-type lectin domain family 10, member A | *Clec10a* | -0.664 | 0.014 |
| 1421529_a_at | thioredoxin reductase 1 | *Txnrd1* | 0.448 | 0.014 |
| 1440209_at | membrane-associated ring finger (C3HC4) 1 | *March1* | -0.654 | 0.014 |
| 1433604_x_at | aldolase A, fructose-bisphosphate | *Aldoa* | 0.496 | 0.014 |
| 1421992_a_at | insulin-like growth factor binding protein 4 | *Igfbp4* | -0.463 | 0.015 |
| 1424524_at | DNA-damage regulated autophagy modulator 1 | *Dram1* | 0.445 | 0.015 |
| 1456204_at | RIKEN cDNA 2010107H07 gene | *2010107H07Rik* | -0.603 | 0.015 |
| 1419315_at | SLAM family member 9 | *Slamf9* | 0.473 | 0.015 |
| 1420965_a_at | ectodermal-neural cortex 1 | *Enc1* | -0.922 | 0.015 |
| 1455269_a_at | coronin, actin binding protein 1A | *Coro1a* | -0.447 | 0.015 |
| 1454973_at | activating transcription factor 7 interacting protein | *Atf7ip* | -0.494 | 0.015 |
| 1435492_at | suppressor of cytokine signaling 6 | *Socs6* | -0.563 | 0.015 |
| 1428574_a_at | chimerin (chimaerin) 2 | *Chn2* | -0.446 | 0.015 |
| 1452347_at | myocyte enhancer factor 2A | *Mef2a* | -0.450 | 0.015 |
| 1424451_at | acetyl-Coenzyme A acyltransferase 1B | *Acaa1b* | -0.439 | 0.015 |
| 1430388_a_at | sulfatase 2 | *Sulf2* | -0.507 | 0.016 |
| 1448502_at | solute carrier family 16  (monocarboxylic acid transporters), member 7 | *Slc16a7* | -0.530 | 0.016 |
| 1445292_at | CD300A antigen | *Cd300a* | -0.441 | 0.016 |
| 1438115_a_at | solute carrier family 9 (sodium/hydrogen exchanger), member 3 regulator 1 | *Slc9a3r1* | -0.457 | 0.016 |
| 1428988_at | ATP-binding cassette, sub-family C (CFTR/MRP), member 3 | *Abcc3* | -0.681 | 0.016 |
| 1434380_at | guanylate binding protein 7 | *Gbp7* | 0.459 | 0.016 |
| 1424828_a_at | fumarate hydratase 1 | *Fh1* | -0.594 | 0.016 |
| 1444361_at | NA | *NA* | -0.455 | 0.016 |
| 1433711_s_at | sestrin 1 | *Sesn1* | -0.575 | 0.016 |
| 1453289_at | eukaryotic translation initiation factor 2C, 4 | *Eif2c4* | -0.620 | 0.016 |
| 1449176_a_at | deoxycytidine kinase | *Dck* | 0.457 | 0.016 |
| 1444203_at | NA | *NA* | 0.455 | 0.016 |
| 1423835_at | zinc finger protein 503 | *Zfp503* | 0.476 | 0.016 |
| 1434297_at | RIKEN cDNA E130304F04 gene | *E130304F04Rik* | -0.453 | 0.016 |
| 1435945_a_at | potassium intermediate/small conductance calcium-activated channel, subfamily N, member 4 | *Kcnn4* | 0.482 | 0.017 |
| 1426008_a_at | solute carrier family 7 (cationic amino acid transporter, y+ system), member 2 | *Slc7a2* | 0.443 | 0.017 |
| 1418002_at | HIG1 domain family, member 2A | *Higd2a* | -0.519 | 0.017 |
| 1425718_a_at | influenza virus NS1A binding protein | *Ivns1abp* | -0.694 | 0.017 |
| 1434403_at | sprouty-related, EVH1 domain containing 2 | *Spred2* | 0.435 | 0.017 |
| 1452360_a_at | lysine (K)-specific demethylase 5A | *Kdm5a* | -0.454 | 0.017 |
| **1441887_x_at** | **predicted gene 6377** | ***Gm6377*** | 0.477 | 0.017 |
| 1438957_x_at | CDP-diacylglycerol synthase  (phosphatidate cytidylyltransferase) 2 | *Cds2* | 0.434 | 0.017 |
| 1418985_at | CTTNBP2 N-terminal like | *Cttnbp2nl* | -0.433 | 0.017 |
| 1424029_at | TSPY-like 4 | *Tspyl4* | -0.457 | 0.017 |
| 1423571_at | sphingosine-1-phosphate receptor 1 | *S1pr1* | -0.442 | 0.017 |
| 1449379_at | kinase insert domain protein receptor | *Kdr* | 0.525 | 0.017 |
| 1417584_at | solute carrier family 11 (proton-coupled divalent  metal ion transporters), member 2 | *Slc11a2* | 0.433 | 0.017 |
| 1456940_at | solute carrier family 43, member 2 | *Slc43a2* | -0.614 | 0.017 |
| 1450199_a_at | stabilin 1 | *Stab1* | -0.612 | 0.018 |
| 1451421_a_at | rogdi homolog (Drosophila) | *Rogdi* | -0.429 | 0.018 |
| 1456163_at | family with sequence similarity 72, member A | *Fam72a* | 0.428 | 0.018 |
| 1427351_s_at | immunoglobulin heavy constant mu | *Ighm* | -0.729 | 0.018 |
| 1417185_at | lymphocyte antigen 6 complex, locus A | *Ly6a* | 0.645 | 0.018 |
| 1459984_at | melanoma inhibitory activity 3 | *Mia3* | -0.493 | 0.018 |
| 1448715_x_at | NA | *NA* | -0.497 | 0.018 |
| 1448163_at | glucosamine-6-phosphate deaminase 1 | *Gnpda1* | -0.428 | 0.018 |
| 1450322_s_at | NA | *NA* | 0.458 | 0.018 |
| 1420649_at | zinc finger homeobox 3 | *Zfhx3* | -0.432 | 0.018 |
| 1445421_at | NA | *NA* | -0.564 | 0.018 |
| 1418004_a_at | transmembrane protein 176B | *Tmem176b* | -0.532 | 0.018 |
| 1417654_at | syndecan 4 | *Sdc4* | 0.446 | 0.018 |
| 1439830_at | mitogen-activated protein kinase kinase kinase 5 | *Map3k5* | -0.584 | 0.018 |
| 1426519_at | procollagen-proline, 2-oxoglutarate 4-dioxygenase (proline 4-hydroxylase), alpha 1 polypeptide | *P4ha1* | 0.498 | 0.019 |
| 1425128_at | UDP-GlcNAc:betaGal beta-1,3-N-acetylglucosaminyltransferase 8 | *B3gnt8* | -0.461 | 0.019 |
| 1418578_at | diacylglycerol kinase, alpha | *Dgka* | -0.424 | 0.019 |
| 1435397_at | zinc finger and BTB domain containing 44 | *Zbtb44* | -0.556 | 0.019 |
| 1458802_at | human immunodeficiency virus type I enhancer binding protein 3 | *Hivep3* | 0.423 | 0.019 |
| 1439766_x_at | vascular endothelial growth factor C | *Vegfc* | 0.426 | 0.019 |
| 1449168_a_at | NA | *NA* | 0.440 | 0.019 |
| 1436982_at | trinucleotide repeat containing 6b | *Tnrc6b* | -0.486 | 0.019 |
| 1427313_at | prostaglandin I receptor (IP) | *Ptgir* | 0.421 | 0.019 |
| 1426875_s_at | sulfiredoxin 1 homolog (S. cerevisiae) | *Srxn1* | 0.492 | 0.019 |
| 1424463_at | major facilitator superfamily domain containing 6 | *Mfsd6* | -0.602 | 0.019 |
| 1415929_at | microtubule-associated protein 1 light chain 3 beta | *Map1lc3b* | -0.445 | 0.019 |
| 1430527_a_at | ring finger protein 167 | *Rnf167* | -0.421 | 0.019 |
| 1451992_at | adrenergic receptor kinase, beta 1 | *Adrbk1* | -0.420 | 0.019 |
| 1449708_s_at | checkpoint kinase 1 | *Chek1* | 0.425 | 0.019 |
| 1423672_at | tetratricopeptide repeat domain 30B | *Ttc30b* | -0.502 | 0.019 |
| 1423489_at | monocyte to macrophage differentiation-associated | *Mmd* | -0.473 | 0.019 |
| 1438027_at | NA | *NA* | 0.441 | 0.020 |
| 1421307_at | carbonic anhydrase 13 | *Car13* | 0.420 | 0.020 |
| 1423704_at | phospholipase A2, group XV | *Pla2g15* | -0.482 | 0.020 |
| 1416773_at | WEE 1 homolog 1 (S. pombe) | *Wee1* | 0.419 | 0.020 |
| 1438415_s_at | Yip1 domain family, member 2 | *Yipf2* | -0.445 | 0.020 |
| 1450716_at | a disintegrin-like and metallopeptidase (reprolysin type) with thrombospondin type 1 motif, 1 | *Adamts1* | 0.488 | 0.020 |
| 1422741_a_at | bobby sox homolog (Drosophila) | *Bbx* | -0.480 | 0.020 |
| 1424147_at | AHA1, activator of heat shock protein ATPase homolog 1 (yeast) | *Ahsa1* | 0.422 | 0.021 |
| 1422962_a_at | proteasome (prosome, macropain) subunit, beta type 8 (large multifunctional peptidase 7) | *Psmb8* | 0.424 | 0.021 |
| 1426834_s_at | RIKEN cDNA D930015E06 gene | *D930015E06Rik* | -0.430 | 0.021 |
| 1439343_at | NA | *NA* | -0.416 | 0.021 |
| 1428750_at | CDC42 effector protein (Rho GTPase binding) 2 | *Cdc42ep2* | 0.479 | 0.021 |
| 1424171_a_at | hydroxyacyl glutathione hydrolase | *Hagh* | -0.585 | 0.021 |
| 1454993_a_at | serine/arginine-rich splicing factor 3 | *Srsf3* | 0.416 | 0.021 |
| 1450714_at | antizyme inhibitor 1 | *Azin1* | 0.418 | 0.021 |
| **1418025_at** | **basic helix-loop-helix family, member e40** | ***Bhlhe40*** | 0.429 | 0.021 |
| 1422006_at | eukaryotic translation initiation factor 2-alpha  kinase 2 | *Eif2ak2* | 0.440 | 0.021 |
| 1435108_at | Rho GTPase activating protein 22 | *Arhgap22* | -0.465 | 0.021 |
| 1450842_a_at | centromere protein A | *Cenpa* | -0.421 | 0.021 |
| 1416788_a_at | isocitrate dehydrogenase 3 (NAD+), gamma | *Idh3g* | -0.436 | 0.021 |
| 1448749_at | pleckstrin | *Plek* | 0.413 | 0.021 |
| 1416105_at | nicotinamide nucleotide transhydrogenase | *Nnt* | -0.507 | 0.021 |
| 1423785_at | EGL nine homolog 1 (C. elegans) | *Egln1* | 0.455 | 0.021 |
| 1417308_at | pyruvate kinase, muscle | *Pkm2* | 0.471 | 0.022 |
| 1426373_at | ski sarcoma viral oncogene homolog (avian) | *Ski* | -0.466 | 0.022 |
| 1449065_at | acyl-CoA thioesterase 1 | *Acot1* | -0.435 | 0.022 |
| 1429049_at | NUAK family, SNF1-like kinase, 2 | *Nuak2* | -0.480 | 0.022 |
| 1425156_at | guanylate binding protein 7 | *Gbp7* | 0.438 | 0.022 |
| 1429503_at | family with sequence similarity 69, member A | *Fam69a* | -0.443 | 0.022 |
| 1437871_at | phosphoglucomutase 5 | *Pgm5* | 0.419 | 0.022 |
| 1449106_at | glutathione peroxidase 3 | *Gpx3* | -0.461 | 0.022 |
| 1424358_at | ubiquitin-conjugating enzyme E2E 2 (UBC4/5 homolog, yeast) | *Ube2e2* | 0.412 | 0.022 |
| 1452598_at | GINS complex subunit 1 (Psf1 homolog) | *Gins1* | 0.415 | 0.022 |
| 1439106_at | zinc finger protein 462 | *Zfp462* | 0.466 | 0.022 |
| 1445068_at | mucosa associated lymphoid tissue lymphoma translocation gene 1 | *Malt1* | 0.464 | 0.022 |
| 1419256_at | spectrin beta 2 | *Spnb2* | -0.411 | 0.022 |
| 1441855_x_at | chemokine (C-X-C motif) ligand 1 | *Cxcl1* | 0.410 | 0.022 |
| 1428306_at | DNA-damage-inducible transcript 4 | *Ddit4* | 0.522 | 0.022 |
| 1422703_at | glycerol kinase | *Gyk* | 0.412 | 0.022 |
| 1455065_x_at | NA | *NA* | -0.447 | 0.022 |
| 1452677_at | polyribonucleotide nucleotidyltransferase 1 | *Pnpt1* | 0.420 | 0.023 |
| 1449815_a_at | single-stranded DNA binding protein 2 | *Ssbp2* | -0.610 | 0.023 |
| 1419564_at | zinc finger protein 467 | *Zfp467* | -0.419 | 0.023 |
| 1457293_at | zinc finger and BTB domain containing 4 | *Zbtb4* | -0.433 | 0.023 |
| 1453013_at | zinc finger protein 740 | *Zfp740* | -0.418 | 0.023 |
| 1419280_at | phosphatidylinositol-5-phosphate 4-kinase,  type II, alpha | *Pip4k2a* | -0.522 | 0.023 |
| 1448891_at | Fc receptor-like S, scavenger receptor | *Fcrls* | -0.819 | 0.023 |
| 1424032_at | hydrogen voltage-gated channel 1 | *Hvcn1* | 0.420 | 0.023 |
| 1416592_at | glutaredoxin | *Glrx* | 0.524 | 0.023 |
| 1416315_at | abhydrolase domain containing 4 | *Abhd4* | -0.483 | 0.023 |
| 1431611_a_at | cell adhesion molecule 1 | *Cadm1* | -0.424 | 0.023 |
| 1425981_a_at | retinoblastoma-like 2 | *Rbl2* | -0.459 | 0.023 |
| 1422603_at | ribonuclease, RNase A family 4 | *Rnase4* | -0.477 | 0.023 |
| 1421252_a_at | myocyte enhancer factor 2A | *Mef2a* | -0.436 | 0.023 |
| 1417884_at | solute carrier family 16  (monocarboxylic acid transporters), member 6 | *Slc16a6* | -0.575 | 0.023 |
| 1437025_at | CD28 antigen | *Cd28* | -0.482 | 0.023 |
| 1426975_at | amplified in osteosarcoma | *Os9* | -0.410 | 0.023 |
| 1416897_at | poly (ADP-ribose) polymerase family, member 9 | *Parp9* | 0.406 | 0.023 |
| 1417474_at | intraflagellar transport 46 homolog (Chlamydomonas) | *Ift46* | -0.409 | 0.023 |
| 1417394_at | Kruppel-like factor 4 (gut) | *Klf4* | -0.416 | 0.023 |
| 1425014_at | nuclear receptor subfamily 2, group C, member 2 | *Nr2c2* | -0.407 | 0.023 |
| 1455220_at | frequently rearranged in advanced  T cell lymphomas 2 | *Frat2* | -0.469 | 0.023 |
| 1449679_s_at | syntaxin 5A | *Stx5a* | -0.429 | 0.023 |
| 1420819_at | src-like adaptor | *Sla* | -0.563 | 0.023 |
| 1455316_x_at | cDNA sequence BC094435 | *BC094435* | -0.490 | 0.023 |
| 1418318_at | ring finger protein 128 | *Rnf128* | 0.405 | 0.024 |
| 1416789_at | isocitrate dehydrogenase 3 (NAD+), gamma | *Idh3g* | -0.449 | 0.024 |
| 1438116_x_at | solute carrier family 9 (sodium/hydrogen exchanger), member 3 regulator 1 | *Slc9a3r1* | -0.432 | 0.024 |
| 1438004_at | PAP associated domain containing 7 | *Papd7* | 0.420 | 0.024 |
| 1417000_at | ankyrin repeat and BTB (POZ) domain containing 1 | *Abtb1* | -0.417 | 0.024 |
| 1429537_at | serine/arginine-rich splicing factor 18 | *Sfrs18* | -0.530 | 0.024 |
| 1434929_at | cDNA sequence BC035044 | *BC035044* | -0.574 | 0.024 |
| 1431050_at | ribosomal protein S6 kinase, polypeptide 5 | *Rps6ka5* | -0.486 | 0.024 |
| 1436544_at | ATPase, class V, type 10D | *Atp10d* | -0.476 | 0.024 |
| 1419238_at | ATP-binding cassette, sub-family A (ABC1),  member 7 | *Abca7* | -0.403 | 0.024 |
| 1451206_s_at | cytohesin 1 interacting protein | *Cytip* | -0.595 | 0.024 |
| 1416007_at | special AT-rich sequence binding protein 1 | *Satb1* | -0.435 | 0.024 |
| 1423475_at | cyclin M2 | *Cnnm2* | -0.522 | 0.024 |
| 1429515_at | ubiquitin protein ligase E3 component n-recognin 2 | *Ubr2* | -0.406 | 0.025 |
| 1434191_at | alkylglycerol monooxygenase | *Agmo* | -0.411 | 0.025 |
| 1416206_at | signal-induced proliferation associated gene 1 | *Sipa1* | -0.414 | 0.025 |
| 1420611_at | protein kinase, cAMP dependent, catalytic, beta | *Prkacb* | -0.434 | 0.025 |
| 1417756_a_at | lymphocyte specific 1 | *Lsp1* | -0.414 | 0.025 |
| 1438322_x_at | farnesyl diphosphate farnesyl transferase 1 | *Fdft1* | 0.435 | 0.025 |
| 1418077_at | tripartite motif-containing 21 | *Trim21* | 0.400 | 0.025 |
| 1449271_a_at | heme binding protein 2 | *Hebp2* | -0.406 | 0.025 |
| 1437465_a_at | prolyl 4-hydroxylase, beta polypeptide | *P4hb* | 0.413 | 0.025 |
| 1416295_a_at | interleukin 2 receptor, gamma chain | *Il2rg* | 0.405 | 0.025 |
| **1434130_at** | **lipoma HMGIC fusion partner-like 2** | ***Lhfpl2*** | 0.417 | 0.025 |
| 1451860_a_at | tripartite motif-containing 30A | *Trim30a* | 0.441 | 0.025 |
| 1450876_at | complement component factor h | *Cfh* | -0.620 | 0.025 |
| 1459740_s_at | uncoupling protein 2 (mitochondrial, proton carrier) | *Ucp2* | -0.404 | 0.025 |
| 1428527_at | sorting nexin 7 | *Snx7* | 0.402 | 0.025 |
| 1438928_x_at | ninjurin 1 | *Ninj1* | 0.427 | 0.025 |
| 1450488_at | chemokine (C-C motif) ligand 24 | *Ccl24* | -0.485 | 0.025 |
| 1452841_at | phosphoglucomutase 2-like 1 | *Pgm2l1* | -0.457 | 0.026 |
| 1449591_at | caspase 4, apoptosis-related cysteine peptidase | *Casp4* | 0.399 | 0.026 |
| 1434151_at | methyltransferase like 7A1 | *Mettl7a1* | -0.399 | 0.026 |
| 1449947_s_at | zinc finger homeobox 3 | *Zfhx3* | -0.533 | 0.026 |
| 1452473_at | proline rich 15 | *Prr15* | 0.398 | 0.026 |
| 1447864_s_at | pogo transposable element with KRAB domain | *Pogk* | 0.424 | 0.026 |
| 1434232_a_at | VMA21 vacuolar H+-ATPase homolog (S. cerevisiae) | *Vma21* | 0.439 | 0.026 |
| 1420973_at | AT rich interactive domain 5B (MRF1-like) | *Arid5b* | -0.442 | 0.026 |
| 1427005_at | polo-like kinase 2 (Drosophila) | *Plk2* | 0.410 | 0.026 |
| 1452907_at | galactosylceramidase | *Galc* | -0.493 | 0.026 |
| 1419665_a_at | nuclear protein 1 | *Nupr1* | 0.440 | 0.026 |
| 1452764_at | suppressor of cytokine signaling 6 | *Socs6* | -0.426 | 0.026 |
| 1418174_at | D site albumin promoter binding protein | *Dbp* | -0.439 | 0.027 |
| 1423974_at | nuclear mitotic apparatus protein 1 | *Numa1* | -0.410 | 0.027 |
| 1437112_at | phospholipase D1 | *Pld1* | -0.462 | 0.027 |
| 1438211_s_at | D site albumin promoter binding protein | *Dbp* | -0.598 | 0.027 |
| 1447204_at | NA | *NA* | 0.452 | 0.027 |
| 1419666_x_at | nuclear protein 1 | *Nupr1* | 0.404 | 0.027 |
| 1439811_at | 5-methyltetrahydrofolate-homocysteine methyltransferase | *Mtr* | -0.405 | 0.027 |
| 1447789_x_at | DEAD (Asp-Glu-Ala-Asp) box polypeptide 6 | *Ddx6* | 0.428 | 0.027 |
| 1450377_at | thrombospondin 1 | *Thbs1* | -0.929 | 0.027 |
| 1425714_a_at | Nfat activating molecule with ITAM motif 1 | *Nfam1* | -0.393 | 0.027 |
| 1440275_at | runt related transcription factor 3 | *Runx3* | 0.393 | 0.027 |
| 1439027_at | N(alpha)-acetyltransferase 25, NatB auxiliary subunit | *Naa25* | 0.423 | 0.027 |
| 1426165_a_at | caspase 3 | *Casp3* | -0.416 | 0.027 |
| 1434451_at | NA | *NA* | -0.545 | 0.028 |
| 1416737_at | glycogen synthase 1, muscle | *Gys1* | 0.407 | 0.028 |
| 1448529_at | thrombomodulin | *Thbd* | -0.479 | 0.028 |
| 1426454_at | Rho, GDP dissociation inhibitor (GDI) beta | *Arhgdib* | -0.392 | 0.028 |
| 1446354_at | RIKEN cDNA C130098B18 gene | *C130098B18Rik* | -0.431 | 0.028 |
| 1448559_at | flotillin 1 | *Flot1* | -0.408 | 0.029 |
| 1416008_at | special AT-rich sequence binding protein 1 | *Satb1* | -0.555 | 0.029 |
| 1456844_at | calcium/calmodulin-dependent protein kinase II, delta | *Camk2d* | 0.396 | 0.029 |
| 1421551_s_at | interferon activated gene 202B | *Ifi202b* | 0.412 | 0.029 |
| 1423214_at | plexin C1 | *Plxnc1* | -0.409 | 0.029 |
| 1418992_at | coagulation factor X | *F10* | 0.395 | 0.029 |
| 1452352_at | cytotoxic T lymphocyte-associated protein 2 beta | *Ctla2b* | -0.733 | 0.029 |
| 1439079_a_at | Erbb2 interacting protein | *Erbb2ip* | -0.493 | 0.029 |
| 1428623_at | plexin A1 | *Plxna1* | 0.388 | 0.029 |
| 1415871_at | transforming growth factor, beta induced | *Tgfbi* | -0.401 | 0.029 |
| 1423176_at | transducer of ErbB-2.1 | *Tob1* | -0.463 | 0.030 |
| 1421911_at | signal transducer and activator of transcription 2 | *Stat2* | 0.413 | 0.030 |
| 1421223_a_at | annexin A4 | *Anxa4* | 0.392 | 0.030 |
| 1450970_at | glutamate oxaloacetate transaminase 1, soluble | *Got1* | -0.396 | 0.030 |
| 1451987_at | arrestin, beta 2 | *Arrb2* | -0.443 | 0.030 |
| 1438683_at | WAS protein family, member 2 | *Wasf2* | -0.429 | 0.031 |
| 1457687_at | B cell leukemia/lymphoma 2 | *Bcl2* | 0.422 | 0.031 |
| 1436395_at | caspase recruitment domain family, member 6 | *Card6* | -0.477 | 0.031 |
| 1427368_x_at | feline sarcoma oncogene | *Fes* | -0.440 | 0.031 |
| 1436125_at | DNA segment, Chr 16, ERATO Doi 472, expressed | *D16Ertd472e* | -0.398 | 0.031 |
| 1426642_at | fibronectin 1 | *Fn1* | -0.400 | 0.031 |
| 1460437_at | cytohesin 4 | *Cyth4* | -0.418 | 0.031 |
| **1439819_at** | **expressed sequence AU015263** | ***AU015263*** | -0.487 | 0.031 |
| 1438183_x_at | sorbitol dehydrogenase | *Sord* | -0.385 | 0.031 |
| 1418907_at | coagulation factor V | *F5* | -0.503 | 0.031 |
| 1421038_a_at | potassium intermediate/small conductance calcium-activated channel, subfamily N, member 4 | *Kcnn4* | 0.395 | 0.031 |
| 1447738_s_at | ankyrin repeat domain 13 family, member D | *Ankrd13d* | -0.393 | 0.031 |
| 1417892_a_at | sirtuin 3 (silent mating type information regulation 2, homolog) 3 (S. cerevisiae) | *Sirt3* | -0.546 | 0.031 |
| AFFXGapdhMur/  M32599_M_at | glyceraldehyde-3-phosphate dehydrogenase | *Gapdh* | 0.497 | 0.031 |
| 1437494_at | mitogen-activated protein kinase-activated protein kinase 3 | *Mapkapk3* | -0.411 | 0.031 |
| 1434955_at | membrane-associated ring finger (C3HC4) 1 | *March1* | -0.538 | 0.031 |
| 1452620_at | phosphoenolpyruvate carboxykinase 2 (mitochondrial) | *Pck2* | -0.395 | 0.031 |
| 1435142_at | SFT2 domain containing 2 | *Sft2d2* | -0.418 | 0.031 |
| 1437132_x_at | neural precursor cell expressed,  developmentally down-regulated gene 9 | *Nedd9* | -0.499 | 0.031 |
| 1416250_at | B cell translocation gene 2, anti-proliferative | *Btg2* | -0.633 | 0.031 |
| 1449089_at | nuclear receptor interacting protein 1 | *Nrip1* | -0.382 | 0.032 |
| 1439375_x_at | aldolase A, fructose-bisphosphate | *Aldoa* | 0.397 | 0.032 |
| 1437127_at | zinc finger protein 945 | *Zfp945* | -0.466 | 0.032 |
| 1442884_at | hepatocyte growth factor | *Hgf* | -0.385 | 0.032 |
| 1421028_a_at | myocyte enhancer factor 2C | *Mef2c* | -0.456 | 0.032 |
| 1428669_at | brain expressed myelocytomatosis oncogene | *Bmyc* | -0.438 | 0.032 |
| 1450677_at | checkpoint kinase 1 | *Chek1* | 0.382 | 0.032 |
| 1439107_a_at | myeloid/lymphoid or mixed-lineage leukemia 5 | *Mll5* | -0.382 | 0.032 |
| 1419282_at | chemokine (C-C motif) ligand 12 | *Ccl12* | 0.515 | 0.032 |
| 1437265_at | NA | *NA* | -0.424 | 0.032 |
| 1437395_at | zinc finger, CCHC domain containing 11 | *Zcchc11* | -0.477 | 0.032 |
| 1452094_at | procollagen-proline, 2-oxoglutarate 4-dioxygenase (proline 4-hydroxylase), alpha 1 polypeptide | *P4ha1* | 0.431 | 0.032 |
| 1452796_at | differentially expressed in FDCP 6 | *Def6* | -0.381 | 0.032 |
| 1423831_at | protein kinase, AMP-activated,  gamma 2 non-catalytic subunit | *Prkag2* | -0.498 | 0.032 |
| 1421547_at | CD180 antigen | *Cd180* | 0.382 | 0.032 |
| 1426246_at | protein S (alpha) | *Pros1* | -0.466 | 0.032 |
| 1435697_a_at | cytohesin 1 interacting protein | *Cytip* | -0.571 | 0.032 |
| 1443923_at | A kinase (PRKA) anchor protein 13 | *Akap13* | -0.410 | 0.032 |
| 1448200_at | transcobalamin 2 | *Tcn2* | -0.573 | 0.032 |
| 1429234_s_at | septin 11 | *Sept11* | 0.383 | 0.032 |
| 1424478_at | Bardet-Biedl syndrome 2 (human) | *Bbs2* | -0.405 | 0.032 |
| 1438294_at | ataxin 1 | *Atxn1* | -0.523 | 0.032 |
| 1448831_at | angiopoietin 2 | *Angpt2* | 0.393 | 0.032 |
| 1424518_at | NA | *NA* | 0.392 | 0.032 |
| 1423518_at | c-src tyrosine kinase | *Csk* | -0.387 | 0.033 |
| 1447231_at | NA | *NA* | -0.384 | 0.033 |
| 1428749_at | Dmx-like 2 | *Dmxl2* | -0.455 | 0.033 |
| 1439497_at | ataxin 7-like 1 | *Atxn7l1* | -0.393 | 0.033 |
| 1452178_at | NA | *NA* | 0.378 | 0.033 |
| 1459741_x_at | uncoupling protein 2 (mitochondrial, proton carrier) | *Ucp2* | -0.381 | 0.033 |
| 1441709_at | solute carrier family 11 (proton-coupled divalent metal ion transporters), member 2 | *Slc11a2* | 0.380 | 0.033 |
| 1424500_at | UTP6, small subunit (SSU) processome component, homolog (yeast) | *Utp6* | 0.384 | 0.033 |
| 1422776_at | serine (or cysteine) peptidase inhibitor, clade B, member 8 | *Serpinb8* | 0.404 | 0.033 |
| 1460344_at | pre B cell leukemia transcription factor interacting protein 1 | *Pbxip1* | -0.472 | 0.034 |
| 1438931_s_at | sestrin 1 | *Sesn1* | -0.446 | 0.034 |
| 1417605_s_at | calcium/calmodulin-dependent protein kinase I | *Camk1* | -0.378 | 0.034 |
| 1422264_s_at | Kruppel-like factor 9 | *Klf9* | -0.402 | 0.034 |
| 1417130_s_at | angiopoietin-like 4 | *Angptl4* | -0.512 | 0.034 |
| 1432466_a_at | apolipoprotein E | *Apoe* | -0.632 | 0.034 |
| 1453108_at | arylsulfatase K | *Arsk* | -0.433 | 0.034 |
| 1451285_at | fusion, derived from t(12;16) malignant liposarcoma (human) | *Fus* | 0.397 | 0.034 |
| 1446860_at | NA | *NA* | -0.376 | 0.034 |
| 1419124_at | major facilitator superfamily domain containing 6 | *Mfsd6* | -0.405 | 0.034 |
| 1424826_s_at | metastasis suppressor 1 | *Mtss1* | 0.392 | 0.034 |
| 1439965_at | solute carrier family 43, member 2 | *Slc43a2* | -0.444 | 0.034 |
| 1433766_at | N(alpha)-acetyltransferase 25, NatB auxiliary subunit | *Naa25* | 0.385 | 0.034 |
| 1423975_s_at | nuclear mitotic apparatus protein 1 | *Numa1* | -0.421 | 0.034 |
| 1416811_s_at | NA | *NA* | -0.944 | 0.034 |
| 1438050_x_at | predicted gene 9222 | *Gm9222* | -0.392 | 0.034 |
| 1455940_x_at | WD repeat domain 6 | *Wdr6* | -0.421 | 0.034 |
| 1456893_at | NA | *NA* | -0.417 | 0.034 |
| 1448188_at | uncoupling protein 2 (mitochondrial, proton carrier) | *Ucp2* | -0.377 | 0.034 |
| 1419456_at | dicarbonyl L-xylulose reductase | *Dcxr* | -0.420 | 0.034 |
| 1430353_at | GLIS family zinc finger 3 | *Glis3* | -0.452 | 0.034 |
| 1428340_s_at | ATPase type 13A2 | *Atp13a2* | -0.431 | 0.035 |
| 1448471_a_at | cytotoxic T lymphocyte-associated protein 2 alpha | *Ctla2a* | -1.057 | 0.035 |
| 1452588_at | zinc finger protein 688 | *Zfp688* | -0.381 | 0.035 |
| 1426025_s_at | lysosomal-associated protein transmembrane 5 | *Laptm5* | -0.443 | 0.035 |
| 1429779_at | eukaryotic translation initiation factor 2C, 4 | *Eif2c4* | -0.524 | 0.035 |
| 1415824_at | stearoyl-Coenzyme A desaturase 2 | *Scd2* | 0.526 | 0.035 |
| 1424172_at | hydroxyacyl glutathione hydrolase | *Hagh* | -0.509 | 0.035 |
| 1434278_at | X-linked myotubular myopathy gene 1 | *Mtm1* | -0.375 | 0.035 |
| 1417649_at | cyclin-dependent kinase inhibitor 1C (P57) | *Cdkn1c* | 0.563 | 0.035 |
| 1454858_x_at | methyltransferase like 7A1 | *Mettl7a1* | -0.374 | 0.035 |
| 1416481_s_at | HIG1 domain family, member 1A | *Higd1a* | 0.417 | 0.035 |
| 1415743_at | histone deacetylase 5 | *Hdac5* | -0.397 | 0.035 |
| 1441229_at | RIKEN cDNA D230019N24 gene | *D230019N24Rik* | -0.377 | 0.035 |
| 1416195_at | inositol polyphosphate 5-phosphatase K | *Inpp5k* | -0.418 | 0.036 |
| 1460228_at | upstream transcription factor 2 | *Usf2* | -0.459 | 0.036 |
| 1450403_at | signal transducer and activator of transcription 2 | *Stat2* | 0.386 | 0.036 |
| 1450165_at | schlafen 2 | *Slfn2* | 0.376 | 0.036 |
| 1460302_at | thrombospondin 1 | *Thbs1* | -1.024 | 0.036 |
| 1429123_at | RAB27A, member RAS oncogene family | *Rab27a* | -0.393 | 0.036 |
| 1448468_a_at | potassium voltage-gated channel, shaker-related subfamily, beta member 1 | *Kcnab1* | 0.376 | 0.036 |
| 1450444_a_at | nuclear receptor subfamily 1, group H, member 3 | *Nr1h3* | 0.437 | 0.036 |
| 1434339_at | formin binding protein 1-like | *Fnbp1l* | 0.406 | 0.036 |
| 1450698_at | dual specificity phosphatase 2 | *Dusp2* | 0.371 | 0.036 |
| 1424613_at | G protein-coupled receptor, family C, group 5, member B | *Gprc5b* | 0.389 | 0.036 |
| 1457285_at | zinc finger protein 187 | *Zfp187* | -0.471 | 0.036 |
| 1438058_s_at | prostate tumor over expressed gene 1 | *Ptov1* | -0.398 | 0.036 |
| 1421922_at | SH3-domain binding protein 5 (BTK-associated) | *Sh3bp5* | -0.387 | 0.036 |
| 1457069_at | activating signal cointegrator 1 complex subunit 3 | *Ascc3* | 0.386 | 0.037 |
| 1435058_x_at | syntaxin binding protein 3A | *Stxbp3a* | -0.416 | 0.037 |
| 1448165_at | caspase 2 | *Casp2* | -0.436 | 0.037 |
| 1460006_at | zinc finger homeobox 3 | *Zfhx3* | -0.430 | 0.037 |
| 1428144_at | leucine-rich repeats and WD repeat domain containing 1 | *Lrwd1* | -0.380 | 0.037 |
| 1425193_at | RIKEN cDNA 2010106G01 gene | *2010106G01Rik* | 0.369 | 0.037 |
| 1436124_at | phosphate cytidylyltransferase 1, choline, beta isoform | *Pcyt1b* | -0.375 | 0.037 |
| 1434975_x_at | phosphatidylserine decarboxylase, pseudogene 3 | *Pisd-ps3* | -0.515 | 0.037 |
| 1449300_at | CTTNBP2 N-terminal like | *Cttnbp2nl* | -0.395 | 0.037 |
| 1429239_a_at | StAR-related lipid transfer (START)  domain containing 4 | *Stard4* | 0.428 | 0.037 |
| 1433446_at | 3-hydroxy-3-methylglutaryl-Coenzyme A synthase 1 | *Hmgcs1* | 0.380 | 0.037 |
| 1424634_at | transcription elongation factor A (SII)-like 1 | *Tceal1* | -0.389 | 0.037 |
| 1425426_a_at | myocyte enhancer factor 2A | *Mef2a* | -0.374 | 0.037 |
| 1416881_at | myeloid cell leukemia sequence 1 | *Mcl1* | 0.372 | 0.037 |
| 1421685_at | C-type lectin domain family 4, member b1 | *Clec4b1* | -0.375 | 0.037 |
| 1458718_at | NA | *NA* | -0.368 | 0.037 |
| 1460603_at | sterile alpha motif domain containing 9-like | *Samd9l* | 0.399 | 0.037 |
| 1452639_at | ectonucleotide pyrophosphatase/phosphodiesterase 4 | *Enpp4* | 0.488 | 0.037 |
| 1425407_s_at | NA | *NA* | -0.378 | 0.038 |
| 1457279_at | predicted gene, 17586 | *Gm17586* | -0.412 | 0.038 |
| 1423066_at | DNA methyltransferase 3A | *Dnmt3a* | 0.453 | 0.038 |
| 1424988_at | myosin regulatory light chain interacting protein | *Mylip* | -0.656 | 0.038 |
| 1418993_s_at | coagulation factor X | *F10* | 0.439 | 0.038 |
| 1424289_at | oxidative stress induced growth inhibitor family member 2 | *Osgin2* | 0.379 | 0.038 |
| **1452160_at** | **TCDD-inducible poly(ADP-ribose) polymerase** | ***Tiparp*** | 0.367 | 0.038 |
| 1456778_at | NA | *NA* | 0.371 | 0.038 |
| 1452410_a_at | feline sarcoma oncogene | *Fes* | -0.425 | 0.038 |
| 1417888_at | tripartite motif-containing 13 | *Trim13* | 0.382 | 0.038 |
| 1423597_at | ATPase, aminophospholipid transporter (APLT),  class I, type 8A, member 1 | *Atp8a1* | -0.426 | 0.038 |
| 1419026_at | Fas death domain-associated protein | *Daxx* | 0.371 | 0.038 |
| 1437901_a_at | vacuolar protein sorting 41 (yeast) | *Vps41* | -0.423 | 0.039 |
| 1448985_at | dual specificity phosphatase 22 | *Dusp22* | -0.417 | 0.039 |
| 1460409_at | carnitine palmitoyltransferase 1a, liver | *Cpt1a* | -0.408 | 0.039 |
| 1434512_x_at | serine/arginine-rich splicing factor 3 | *Srsf3* | 0.372 | 0.039 |
| 1430220_at | RIKEN cDNA 4833420G17 gene | *4833420G17Rik* | -0.368 | 0.039 |
| 1426603_at | ribonuclease L (2', 5'-oligoisoadenylate  synthetase-dependent) | *Rnasel* | -0.408 | 0.039 |
| 1424911_a_at | lysozyme-like 4 | *Lyzl4* | 0.383 | 0.039 |
| 1456426_at | C-type lectin domain family 2, member i | *Clec2i* | -0.379 | 0.039 |
| 1449839_at | caspase 3 | *Casp3* | -0.409 | 0.039 |
| 1438992_x_at | activating transcription factor 4 | *Atf4* | 0.400 | 0.039 |
| 1439488_at | DOT1-like, histone H3 methyltransferase (S. cerevisiae) | *Dot1l* | 0.379 | 0.039 |
| 1437989_at | phosphodiesterase 8B | *Pde8b* | 0.367 | 0.039 |
| 1423803_s_at | glioma tumor suppressor candidate region gene 2 | *Gltscr2* | -0.418 | 0.039 |
| 1433963_a_at | fermitin family homolog 3 (Drosophila) | *Fermt3* | -0.373 | 0.039 |
| 1444599_at | hect domain and RLD 4 | *Herc4* | -0.487 | 0.039 |
| 1433986_at | cDNA sequence BC024659 | *BC024659* | -0.447 | 0.039 |
| 1452203_at | oligonucleotide/oligosaccharide-binding fold containing 2A | *Obfc2a* | 0.364 | 0.039 |
| AFFXGapdhMur/  M32599_5_at | glyceraldehyde-3-phosphate dehydrogenase | *Gapdh* | 0.453 | 0.039 |
| 1451986_s_at | leucine-rich repeat kinase 1 | *Lrrk1* | -0.376 | 0.040 |
| 1431292_a_at | twinfilin, actin-binding protein, homolog 2 (Drosophila) | *Twf2* | -0.419 | 0.040 |
| 1453721_a_at | solute carrier family 31, member 2 | *Slc31a2* | 0.366 | 0.040 |
| 1449846_at | eosinophil-associated, ribonuclease A family,  member 2 | *Ear2* | -0.575 | 0.040 |
| 1428375_at | RIKEN cDNA 4932415G12 gene | *4932415G12Rik* | -0.404 | 0.040 |
| 1438719_at | mitogen-activated protein kinase kinase kinase 2 | *Map3k2* | -0.362 | 0.040 |
| 1451110_at | EGL nine homolog 1 (C. elegans) | *Egln1* | 0.394 | 0.040 |
| 1422607_at | ets variant gene 1 | *Etv1* | -0.378 | 0.040 |
| 1426587_a_at | signal transducer and activator of transcription 3 | *Stat3* | -0.371 | 0.040 |
| 1431110_at | plexin domain containing 2 | *Plxdc2* | -0.516 | 0.040 |
| 1417190_at | nicotinamide phosphoribosyltransferase | *Nampt* | 0.389 | 0.040 |
| 1417597_at | CD28 antigen | *Cd28* | -0.437 | 0.040 |
| 1433758_at | nischarin | *Nisch* | -0.367 | 0.040 |
| 1438882_at | NA | *NA* | -0.395 | 0.040 |
| 1431592_a_at | SH3-domain kinase binding protein 1 | *Sh3kbp1* | -0.363 | 0.040 |
| 1422818_at | neural precursor cell expressed, developmentally down-regulated gene 9 | *Nedd9* | -0.414 | 0.041 |
| 1435330_at | pyrin and HIN domain family, member 1 | *Pyhin1* | 0.371 | 0.041 |
| 1425899_a_at | intersectin 1 (SH3 domain protein 1A) | *Itsn1* | -0.362 | 0.041 |
| 1432007_s_at | adaptor protein complex AP-2, alpha 2 subunit | *Ap2a2* | -0.536 | 0.041 |
| 1434649_at | cerebral cavernous malformation 2 homolog (human) | *Ccm2* | -0.401 | 0.041 |
| 1435822_at | RIKEN cDNA D830012I24 gene | *D830012I24Rik* | -0.418 | 0.041 |
| 1442453_at | FCH domain only 2 | *Fcho2* | -0.462 | 0.041 |
| 1437194_x_at | leucine-rich repeats and WD repeat domain containing 1 | *Lrwd1* | -0.391 | 0.041 |
| 1448436_a_at | interferon regulatory factor 1 | *Irf1* | 0.376 | 0.041 |
| 1448859_at | chemokine (C-X-C motif) ligand 13 | *Cxcl13* | 0.360 | 0.041 |
| 1420028_s_at | minichromosome maintenance deficient 3  (S. cerevisiae) | *Mcm3* | 0.365 | 0.041 |
| 1451140_s_at | protein kinase, AMP-activated, gamma 2 non-catalytic subunit | *Prkag2* | -0.387 | 0.041 |
| 1428777_at | sprouty protein with EVH-1 domain 1, related sequence | *Spred1* | 0.367 | 0.041 |
| 1450627_at | progressive ankylosis | *Ank* | -0.362 | 0.041 |
| 1417161_at | CDK2-associated protein 2 | *Cdk2ap2* | -0.368 | 0.042 |
| 1440972_at | nuclear receptor-binding SET-domain protein 1 | *Nsd1* | -0.387 | 0.042 |
| 1443088_at | RIKEN cDNA 9930031P18 gene | *9930031P18Rik* | -0.387 | 0.042 |
| 1439747_at | prostaglandin E synthase | *Ptges* | 0.361 | 0.042 |
| 1441727_s_at | zinc finger protein 467 | *Zfp467* | -0.479 | 0.042 |
| 1451750_at | interleukin-1 receptor-associated kinase 4 | *Irak4* | -0.390 | 0.042 |
| 1423071_x_at | NA | *NA* | 0.361 | 0.042 |
| 1434799_x_at | aldolase A, fructose-bisphosphate | *Aldoa* | 0.389 | 0.042 |
| 1459840_s_at | coiled coil domain containing 28B | *Ccdc28b* | -0.444 | 0.043 |
| 1445831_at | NA | *NA* | -0.392 | 0.043 |
| 1428376_at | RIKEN cDNA 4932415G12 gene | *4932415G12Rik* | -0.366 | 0.043 |
| 1455642_a_at | tetraspanin 17 | *Tspan17* | -0.376 | 0.043 |
| 1434139_at | poly (ADP-ribose) polymerase family, member 11 | *Parp11* | 0.357 | 0.043 |
| 1447999_x_at | glyceraldehyde-3-phosphate dehydrogenase | *Gapdh* | 0.506 | 0.043 |
| 1438999_a_at | nuclear factor of activated T cells 5 | *Nfat5* | -0.401 | 0.043 |
| 1426700_a_at | PAN2 polyA specific ribonuclease subunit homolog (S. cerevisiae) | *Pan2* | -0.366 | 0.043 |
| 1420632_a_at | Bernardinelli-Seip congenital lipodystrophy 2 homolog (human) | *Bscl2* | -0.371 | 0.043 |
| 1450626_at | mannosidase, beta A, lysosomal | *Manba* | -0.385 | 0.043 |
| 1435344_at | transcription factor Dp 2 | *Tfdp2* | -0.361 | 0.043 |
| 1433598_at | arginine and glutamate rich 1 | *Arglu1* | -0.391 | 0.043 |
| 1447364_x_at | myosin IB | *Myo1b* | 0.413 | 0.043 |
| **1434129_s_at** | **lipoma HMGIC fusion partner-like 2** | ***Lhfpl2*** | 0.356 | 0.044 |
| 1427469_at | helicase with zinc finger domain | *Helz* | -0.392 | 0.044 |
| 1450020_at | chemokine (C-X3-C) receptor 1 | *Cx3cr1* | -0.379 | 0.044 |
| AFFXGapdhMur/  M32599_3_at | glyceraldehyde-3-phosphate dehydrogenase | *Gapdh* | 0.440 | 0.044 |
| 1421811_at | thrombospondin 1 | *Thbs1* | -0.767 | 0.044 |
| 1451303_at | cDNA sequence BC002230 | *BC002230* | -0.372 | 0.044 |
| 1415825_s_at | NA | *NA* | -0.378 | 0.044 |
| 1449141_at | filamin binding LIM protein 1 | *Fblim1* | 0.430 | 0.044 |
| 1435679_at | optineurin | *Optn* | 0.355 | 0.044 |
| 1440559_at | high mobility group AT-hook 2, pseudogene 1 | *Hmga2-ps1* | -0.466 | 0.044 |
| 1450009_at | lactotransferrin | *Ltf* | -0.370 | 0.044 |
| 1451352_s_at | metastasis associated 3 | *Mta3* | -0.366 | 0.044 |
| 1424163_at | required for meiotic nuclear division 5 homolog B  (S. cerevisiae) | *Rmnd5b* | -0.364 | 0.044 |
| 1429418_at | CDC14 cell division cycle 14 homolog B (S. cerevisiae) | *Cdc14b* | -0.368 | 0.044 |
| 1448664_a_at | SPEG complex locus | *Speg* | 0.356 | 0.044 |
| 1453849_s_at | heterogeneous nuclear ribonucleoprotein A/B | *Hnrnpab* | 0.355 | 0.044 |
| 1418546_a_at | STAM binding protein like 1 | *Stambpl1* | -0.392 | 0.044 |
| 1456885_at | septin 8 | *Sept8* | -0.378 | 0.044 |
| 1436502_at | mitochondrial tumor suppressor 1 | *Mtus1* | -0.460 | 0.044 |
| 1417399_at | growth arrest specific 6 | *Gas6* | -0.560 | 0.044 |
| 1422704_at | glycerol kinase | *Gyk* | 0.364 | 0.044 |
| 1423488_at | monocyte to macrophage differentiation-associated | *Mmd* | -0.383 | 0.044 |
| 1439477_at | NA | *NA* | -0.523 | 0.044 |
| 1460283_at | Mediterranean fever | *Mefv* | 0.386 | 0.045 |
| 1451126_at | MAF1 homolog (S. cerevisiae) | *Maf1* | -0.367 | 0.045 |
| 1450051_at | alpha thalassemia/mental retardation syndrome X-linked homolog (human) | *Atrx* | -0.436 | 0.045 |
| 1424990_at | ORAI calcium release-activated calcium modulator 1 | *Orai1* | -0.362 | 0.045 |
| 1429192_at | ski sarcoma viral oncogene homolog (avian) | *Ski* | -0.353 | 0.045 |
| 1418114_at | recombination signal binding protein for immunoglobulin kappa J region | *Rbpj* | 0.363 | 0.045 |
| 1422786_at | solute carrier family 30 (zinc transporter), member 1 | *Slc30a1* | 0.358 | 0.045 |
| 1455594_at | exocyst complex component 3 | *Exoc3* | -0.421 | 0.045 |
| 1433827_at | ATPase, aminophospholipid transporter (APLT), class I, type 8A, member 1 | *Atp8a1* | -0.362 | 0.046 |
| 1429863_at | LON peptidase N-terminal domain and ring finger 3 | *Lonrf3* | 0.353 | 0.046 |
| **1424915_s_at** | **RIKEN cDNA 2310044G17 gene** | ***2310044G17Rik*** | 0.352 | 0.046 |
| 1436300_at | dual serine/threonine and tyrosine protein kinase | *Dstyk* | -0.430 | 0.046 |
| 1434881_s_at | potassium channel tetramerisation domain containing 12 | *Kctd12* | -0.419 | 0.046 |
| 1418888_a_at | selenoprotein X 1 | *Sepx1* | -0.390 | 0.046 |
| 1417779_at | nucleoside-triphosphatase, cancer-related | *Ntpcr* | -0.480 | 0.046 |
| 1430771_a_at | mutS homolog 5 (E. coli) | *Msh5* | -0.429 | 0.046 |
| 1428573_at | chimerin (chimaerin) 2 | *Chn2* | -0.381 | 0.047 |
| 1451413_at | calpastatin | *Cast* | -0.403 | 0.047 |
| 1418183_a_at | cytohesin 1 | *Cyth1* | -0.493 | 0.047 |
| 1455694_at | neurobeachin-like 2 | *Nbeal2* | -0.359 | 0.047 |
| 1416014_at | ATP-binding cassette, sub-family E (OABP), member 1 | *Abce1* | 0.392 | 0.047 |
| 1456178_at | BMP and activin membrane-bound inhibitor, pseudogene (Xenopus laevis) | *Bambi-ps1* | 0.364 | 0.047 |
| 1428378_at | zinc finger CCCH type, antiviral 1 | *Zc3hav1* | 0.366 | 0.047 |
| 1417494_a_at | ceruloplasmin | *Cp* | 0.372 | 0.047 |
| 1426387_x_at | NA | *NA* | -0.450 | 0.047 |
| 1439032_at | HAUS augmin-like complex, subunit 2 | *Haus2* | -0.402 | 0.048 |
| 1437241_at | Kruppel-like factor 11 | *Klf11* | -0.411 | 0.048 |
| 1456573_x_at | nicotinamide nucleotide transhydrogenase | *Nnt* | -0.481 | 0.048 |
| 1456262_at | RNA binding motif protein 5 | *Rbm5* | -0.401 | 0.048 |
| 1416619_at | RIKEN cDNA 4632428N05 gene | *4632428N05Rik* | -0.410 | 0.048 |
| 1455016_at | PRP38 pre-mRNA processing factor 38 (yeast) domain containing B | *Prpf38b* | -0.359 | 0.048 |
| 1434134_at | DDB1 and CUL4 associated factor 8 | *Dcaf8* | -0.353 | 0.048 |
| 1457264_at | PHD finger protein 20-like 1 | *Phf20l1* | -0.350 | 0.048 |
| 1423569_at | glycine amidinotransferase (L-arginine:glycine amidinotransferase) | *Gatm* | -0.351 | 0.048 |
| 1424226_at | RIKEN cDNA 9030617O03 gene | *9030617O03Rik* | -0.353 | 0.048 |
| 1436456_at | solute carrier family 38, member 9 | *Slc38a9* | -0.363 | 0.048 |
| 1430820_a_at | bobby sox homolog (Drosophila) | *Bbx* | -0.404 | 0.048 |
| 1428666_at | asparaginyl-tRNA synthetase | *Nars* | -0.384 | 0.048 |
| 1415739_at | RNA binding motif protein 42 | *Rbm42* | -0.348 | 0.048 |
| 1417903_at | deafness, autosomal dominant 5 (human) | *Dfna5* | 0.480 | 0.048 |
| 1425263_a_at | myelin basic protein | *Mbp* | -0.396 | 0.048 |
| 1444287_at | expressed sequence AI853106 | *AI853106* | -0.350 | 0.048 |
| 1426478_at | RAS p21 protein activator 1 | *Rasa1* | -0.348 | 0.049 |
| 1418911_s_at | acyl-CoA synthetase long-chain family member 4 | *Acsl4* | 0.347 | 0.049 |
| 1427185_at | myocyte enhancer factor 2A | *Mef2a* | -0.491 | 0.049 |
| 1451418_a_at | splA/ryanodine receptor domain and SOCS box containing 4 | *Spsb4* | 0.421 | 0.049 |
| 1452866_at | asparaginyl-tRNA synthetase | *Nars* | -0.418 | 0.049 |
| 1454728_s_at | ATPase, aminophospholipid transporter (APLT), class I, type 8A, member 1 | *Atp8a1* | -0.368 | 0.049 |
| 1456261_at | SH3-domain kinase binding protein 1 | *Sh3kbp1* | -0.437 | 0.049 |
| 1426832_at | DEAD/H (Asp-Glu-Ala-Asp/His) box polypeptide 26B | *Ddx26b* | -0.403 | 0.049 |
| 1434704_at | myeloid/lymphoid or mixed-lineage leukemia 5 | *Mll5* | -0.401 | 0.049 |
| 1453369_a_at | FUN14 domain containing 1 | *Fundc1* | -0.357 | 0.049 |
| 1418698_a_at | ferrochelatase | *Fech* | -0.399 | 0.049 |
| 1419758_at | ATP-binding cassette, sub-family B (MDR/TAP), member 1A | *Abcb1a* | -0.476 | 0.049 |
| 1450105_at | a disintegrin and metallopeptidase domain 10 | *Adam10* | -0.359 | 0.049 |
| **1429682_at** | **family with sequence similarity 46, member C** | ***Fam46c*** | 0.356 | 0.049 |
| 1416016_at | NA | *NA* | 0.347 | 0.050 |
| 1451457_at | sterol-C5-desaturase (fungal ERG3, delta-5-desaturase) homolog (S. cerevisae) | *Sc5d* | 0.348 | 0.050 |

Bold font = genes significantly regulated in 1 h and 24 h samples.
