# Supplementary material for: Autophagic digestion of Leishmania major by host macrophages is associated with differential expression of BNIP3, CTSE, and the miRNAs miR-101c, miR-129, and miR-210
Source: Parasit Vectors. 2015 Jul 31;8:404. doi: 10.1186/s13071-015-0974-3 (PMC4521392; doi:10.1186/s13071-015-0974-3)
Supplement: Additional file 12: Figure S7. — Analyses of interactions of miRNAs with target genes in L. m.-infected BMDM 24 h p.i.. Methods: (A) Total RNA was harvested from L. m.-infected BMDM 24 h p.i. and uninfected control BMDM. Affymetrix® chips were hybridized with RNA samples from 2 independent experiments. Significantly differentially expressed miRNAs were predicted bioinformatically to target genes of LISA. (B) Apart from the bioinformatical predictions, published evidence has linked several differentially expressed miRNAs to further autophagy-related genes. Results: (A) Affymetrix® chip analyses revealed 26 differentially expressed miRNAs. Thereof, 14 of these 26 were bioinformatically predicted to target gene products in LISA, which resulted in MONA-of-LISA. (B) Interactions between miRNAs and autophagy-related target genes, which have been previously reported. Letters from a – l are linked with the corresponding literature (a = [82], b = [83], c = [84], d = [63], e = [58], f = [85], g = [89], h = [88], i = [86], j = [59], k = [60], l = [87]). miRNA/mRNA names are linked with databases for additional information. Colors indicate direction of regulation. (Red = upregulation; blue = downregulation). * = miRNA entry on (http://www.mirbase.org/) was removed. [file 13071_2015_974_MOESM12_ESM.pdf]

A

LISA (mRNA) \ MONA (miRNA)	mmu-miR-16-1-3p	mmu-miR-155-5p	mmu-miR-466e-3p	mmu-miR-466p-3p	mmu-miR-466q	mmu-miR-711	mmu-miR-1892	mmu-miR-1955-5p	mmu-miR-3068-3p	mmu-miR-3075-5p	mmu-miR-3473b	mmu-miR-5102 *	mmu-miR-5115 *	mmu-miR-5119	Σ interactions
Abce1			x		x										2
Acsl4	x		x	x	x										4
Ahsa1											x				1
Aldoa											x				1
Ap2a2				x	x							x			3
Ddx6				x			x		x		x	x	x	x	7
Eif2ak2															0
Eno2						x						x			2
Fundc1															0
Fus						x									1
Gapdh										x					1
Hells															0
Hsp90aa1															0
Kctd12			x	x	x	x		x		x		x			7
Ldha					x										1
Map1lc3b						x		x	x		x	x			5
Mcm3			x	x											2
Mif											x				1
Nars															0
Ndrg1								x							1
Nfat5		x	x	x	x				x	x				x	7
Pgk1				x											1
Pkm2								x							1
Prkag2			x												1
Sord						x				x		x		x	4
Trim21															0
Txnrd1						x					x	x	x		4
Zcchc11											x				1
Σ interactions	1	1	6	7	6	6	1	4	3	4	7	7	2	3	

* = removed entry

B

mRNA \ miRNA	let-7f-1 /-2	let-7g	miR-16	miR-101	miR-129-5p	miR-155	miR-210	miR-542-5p	Σ interactions
Atg4a /b /c /d				a, b, c					1
Bcl2			d						1
Bnip3							d, e		1
Egfr	a, b	a, b						f	3
Hif1a							g, h		1
Nfkb1							i		1
Rab5a				a, b, c					1
Rheb						j			1
Sp1					k				1
Stmn1				a, b, c					1
Zcchc11	l	l							2
Σ interactions	2	2	1	3	1	1	3	1	
